# Neural Processing of Speech Sounds in ASD and First-Degree Relatives

**DOI:** 10.1007/s10803-022-05562-7

**Published:** 2022-06-07

**Authors:** Shivani P. Patel, Molly Winston, Janna Guilfoyle, Trent Nicol, Gary E. Martin, Kritika Nayar, Nina Kraus, Molly Losh

**Affiliations:** 1 Roxelyn and Richard Pepper Department of Communication Sciences and Disorders, Northwestern University, 2240 N Campus Dr, Evanston, IL 60208 USA; 2 Department of Communication Sciences and Disorders, St. John’s University, Staten Island, NY USA

**Keywords:** Autism spectrum disorder, Broad autism phenotype, Frequency following response, Pragmatic language, Prosody, Sound processing

## Abstract

Efficient neural encoding of sound plays a critical role in speech and language, and when impaired, may have reverberating effects on communication skills. This study investigated disruptions to neural processing of temporal and spectral properties of speech in individuals with ASD and their parents and found evidence of inefficient temporal encoding of speech sounds in both groups. The ASD group further demonstrated less robust neural representation of spectral properties of speech sounds. Associations between neural processing of speech sounds and language-related abilities were evident in both groups. Parent–child associations were also detected in neural pitch processing. Together, results suggest that atypical neural processing of speech sounds is a heritable ingredient contributing to the ASD language phenotype.

**Supplementary Information:**

The online version contains supplementary material available at 10.1007/s10803-022-05562-7.

## Introduction

Language impairment is a hallmark of autism spectrum disorder (ASD), with pragmatic (or social) language difficulties universally observed, with broad impacts on social functioning (Baltaxe & Simmons, 1977; Landa, 2000; Losh et al., 2012; Peppé et al., 2006; Tager-Flusberg, Edelson, & Luyster, 2011). Contributing to such impairments are differences in prosody, which includes atypical intonation, volume modulation, and speech rate (Diehl & Paul, 2012; McCann & Peppé, 2003; Paul, Augustyn, et al., 2005; Paul, Shriberg, et al., 2005; Shriberg et al., 2001). Atypical prosody is among the first characteristics to differentiate an individual with ASD from peers (Mesibov, 1992; Van Bourgondien & Woods, 1992) and can significantly impact social-communicative success. Differences in auditory or speech processing have also been reported in ASD and may relate to prosodic impairments (Patel et al., 2019; Russo, 2008; Russo et al., 2009; Russo, Larson, et al., 2008; Russo, Trommer, et al., 2008). Importantly, differences in pragmatics, prosody, and auditory processing have also been identified among first-degree relatives of individuals with ASD who are at increased genetic liability to ASD (Landa et al., 1992; Losh et al., 2008; Patel et al., 2019, 2020; Paul et al., 2009; Piven et al., 1997).

The study of first-degree relatives is a powerful approach for probing fundamental, heritable features of ASD in their subclinical expression to inform the underlying biology of the complex ASD phenotype. Prior studies have shown differences among first-degree relatives of individuals with ASD in the domain of social cognition, implicating key brain regions involved in social information processing (Adolphs et al., 2008; Baron-Cohen et al., 2006; Billeci et al., 2016; Losh & Piven, 2007; Losh et al., 2009; Palmen et al., 2005; Sasson et al., 2013; Yucel et al., 2015), and in a number of language-related skills. For instance, relatives of individuals with ASD show less efficient eye-voice coordination, atypical visual attention patterns during language production tasks, atypical audio-vocal integration, and broader differences in pragmatic skills, suggesting diminished fluency in aspects of language processing, which is similar to patterns documented in ASD (Hogan-Brown et al., 2014; Landa et al., 1992; Losh et al., 2008; Nayar et al., 2018; Patel et al., 2019).

Prior work identifying relationships between prosody, auditory and speech processing, and pragmatic language in ASD and typical development demonstrates that neural auditory processing impacts vocal production (Chen et al., 2007; Liu et al., 2010; Patel et al., 2019; Russo, Larson, et al., 2008; Russo, Trommer, et al., 2008). In typical development, evidence suggests that processing of auditory feedback impacts language production, including key aspects of speech like suprasegmentals, which are an area of speech atypicality associated with ASD (Chen et al., 2007). Chen et al. (2007) further concluded that this may be a reflection of linguistic ability and specific neural mechanisms which are similarly impacted in ASD. In a study implementing a pitch-perturbed auditory feedback task among individuals with ASD, during which participants heard their voice in real-time as they vocalized, individuals with ASD and their parents overcorrected for pitch perturbations and produced an even lower or higher pitch than expected relative to controls (Patel et al., 2019). The increased response magnitudes indicated less efficient audio-vocal integration and were closely related to listener-ratings of prosodic abilities. Furthermore, the audio-vocal integration differences were reflected in differences in event-related potential analyses as well (Patel et al., 2019). Close associations between these language domains and neural atypicalities in individuals with ASD and their first-degree relatives suggest heritable mechanisms underlying ASD-related language impairments. Building on this work, the present study investigated neural processing of speech sounds in individuals with ASD and their biological parents, by studying the frequency following response (FFR).

The FFR is a robust neural response to sound that is highly related to speech processing and broader communication skills across the lifespan (Rosenhall et al., 1985; Skoe et al., 2015). While based predominantly in the auditory midbrain, the FFR reflects integrated processing from the auditory periphery and central nervous system, thereby providing valuable information about neural sound processing and downstream influences on communication (Chandrasekaran & Kraus, 2010; Malmierca, 2015; Malmierca & Ryugo, 2011; Sohmer et al., 1977). Prior work using FFR in children with ASD (ages 7–13 years) has reported atypical timing and frequency encoding of speech sounds, and reduced response consistency (Otto-Meyer et al., 2018; Russo et al., 2009). Interestingly, atypicalities in speech sound processing have been documented using FFR in the presence of typical click-evoked sounds and normal hearing status (Klin, 1993; Russo et al., 2009), suggesting that differences observed in ASD are specific to speech. A study examining neural pitch tracking of speech in ASD using FFR found decreased neural tracking of the voice pitch and reduced phase locking (Russo, Larson, et al., 2008; Russo, Trommer, et al., 2008) – differences that could affect higher-level language abilities impacted in ASD, including prosody and pragmatic language (Losh et al., 2012; Patel et al., 2020; Paul, Augustyn, et al., 2005; Paul, Shriberg, et al., 2005; Tager-Flusberg et al., 2011).

Robust associations between FFR and language skills have been demonstrated in the general population across the lifespan (Benasich & Tallal, 2002; Krishnan et al., 2005; Krizman et al., 2012), and in language-related disorders (Banai et al., 2007; Hornickel et al., 2009, 2012; Thomson & Goswami, 2008), though little is known about how atypical FFR might relate to the profile of ASD. Russo and colleagues did not detect associations between FFRs and intellectual functioning or global measures of receptive and expressive language ability in children ages 7–13 (Russo, Larson, et al., 2008; Russo, Trommer, et al., 2008), but pragmatics and prosody were not examined. Given the limited work examining such key language-related correlates of FFR in ASD, it is important to examine FFR in relationship to the prosodic and pragmatic skills impacted in ASD. Examining potential links between FFR and the subclinical expression of ASD-related phenotypes in parents holds additional potential for revealing heritable, neurobiological mechanisms that can help inform the underlying etiology of ASD and its component traits.

**Fig. 1:**
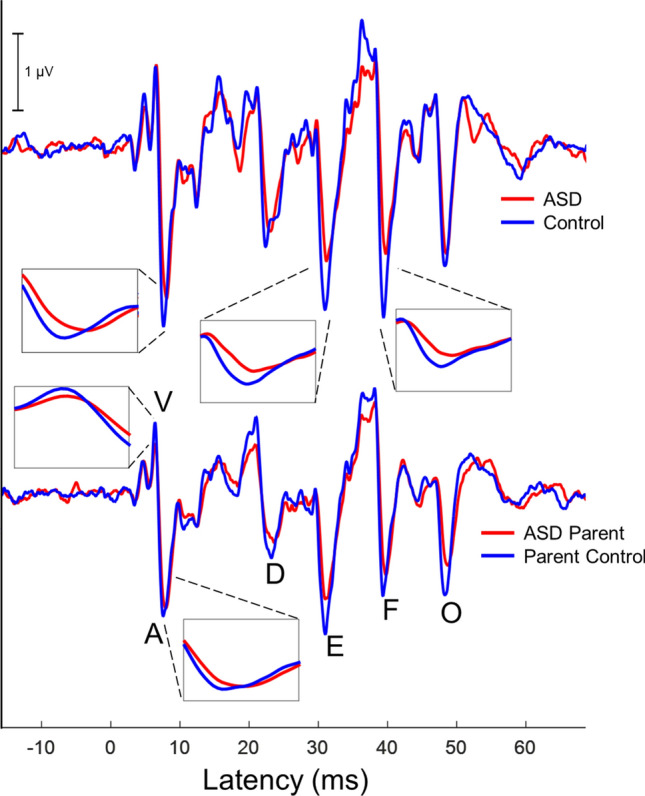
Grand average waveforms for the ASD and Control groups (top) and the ASD Parent and Parent Control groups (bottom). Closeups of peaks with significant group timing differences are shown in insets

This study examined the hypothesis that differences in neural processing of speech sounds contribute to pragmatic and prosodic impairments in ASD, and subclinical differences among first-degree relatives. We collected FFRs to two speech stimuli in individuals with ASD, their parents, and respective control groups. Stimuli included a short speech-evoked /dɑ/ and a longer /jɑ/ with ascending pitch contour to assess neural processing of speech sounds (Bonacina et al., 2019; Russo et al., 2009; Russo, Larson, et al., 2008; Russo, Trommer, et al., 2008). These stimuli were chosen based on availability of extensive normative data and past findings in the ASD population suggesting these stimuli could be most fruitful to examine further in relatives, and in relationship to clinical-behavioral correlates. Responses to the /dɑ/ stimulus index the timing and synchrony of neural responses. Specifically, onset of the neural response is indicated by wave V and its negative trough wave A, while offset of the response is indicated by wave O. Phase locking to the fundamental frequency of the stimulus is reflected by latencies for waves D, E, and F (Chandrasekaran & Kraus, 2010; Krizman et al., 2019, see Fig. 1). The /jɑ/ stimulus provides information regarding the fidelity of neural pitch tracking. Both reflect critical components of complex speech sound processing, strongly implicated in variety of language-related disabilities, including ASD (Anderson & Kraus, 2013; Benasich & Tallal, 2002; Chandrasekaran et al., 2009; Hornickel & Kraus, 2013; Hornickel et al., 2012; Otto-Meyer et al., 2018; Russo et al., 2009; Russo, Larson, et al., 2008; Russo, Trommer, et al., 2008).

Given repeated observations that parents of individuals with ASD may display subclinical pragmatic language differences (Landa et al., 1992; Losh et al., 2008; Piven et al., 1997), and specifically in neural mechanisms contributing to audio-vocal integration impacting prosody (Patel et al., 2019), we predicted that the ASD parent group would display increased neural response latencies and reduced fidelity of neural pitch tracking compared to parent controls. We predicted that atypicalities in FFR would relate to pragmatic and prosodic abilities in individuals with ASD and their parents, and that parent–child associations would emerge in the fidelity of neural response to speech sounds, which would support FFR as a potential heritable neural marker of language-related impairments in ASD.

## Methods

### Participants

Participants were recruited through the Northwestern University Communication Research Registry (P30DC012035), the Northwestern Child Studies Group, existing studies, and by study advertisement. Participants included 34 individuals with autism spectrum disorder (ASD group), 24 typically developing controls (ASD Control group), 49 parents of individuals with ASD (ASD Parent group), and 32 parents of typically developing individuals (Parent Control group). A subset of participants did not complete the full FFR protocol because of sensory aversions or time constraints (ASD group n = 9 (3 females); ASD Control group n = 1 (0 females); ASD Parent group n = 6 (3 females); Parent Control group n = 4 (3 females)). All participants were native English speakers with no history of hearing loss, brain injury, or presence of a known genetic condition other than ASD (e.g., fragile X syndrome). Control participants were screened for family history of ASD and excluded if they had first- or second-degree relatives with ASD or a history of language related impairments. Similarly, participants in the ASD Parent group were screened for personal history of an ASD diagnosis and if indicated, were excluded from the study. All individuals with ASD had a formal diagnosis of autism or autism spectrum disorder. Diagnoses were confirmed using the Autism Diagnostic Observation Schedule-2nd Edition (ADOS-2; Lord et al., 2012) for all participants, as well as the Autism Diagnostic Interview-Revised (ADI-R; Rutter et al., 2003). Nine individuals did not receive the ADI-R because of time limitations for testing. All parents in the ASD Parent group had at least one child with ASD, and every effort was made to include intact parent–child dyads. In some cases, however, a variety of factors, including but not limited to sensory aversions to the electrodes, discomfort during the task, and overactivity, precluded assessment of the child’s FFR.

Intellectual functioning was assessed using the Wechsler Abbreviated Scale of Intelligence (WASI;Wechsler, 1999) for individuals 16 years of age or older and the Wechsler Intelligence Scale for Children-Fourth Edition (WISC-IV; Wechsler, 2003) for individuals younger than 16 years of age. See Table 1 for group comparisons of chronological age and full-scale IQ. All analyses controlled for chronological age given known differences in the frequency following response with changes in age (Bonacina et al., 2019; Skoe et al., 2015).

**Table 1:** Group characteristics

	ASD (*n* = 34)	ASD control (*n* = 24)	Group comparison (ASD vs. ASD Control)	ASD parent (*n* = 49)	Parent control (*n* = 32)	Group comparison (ASD Parent vs. Parent Control)
Sex *Males:Females*	21:13	13:11		17:32	4:28	
Chronological Age *M* (*SD*)	17.12 (6.04)	15.17 (6.34)	*t*(48) = 1.18, *p* = .25	47.14 (8.50)	43.50 (7.25)	*t*(73) = 2.06, *p* = .04
Full scale IQ *M* (*SD*)	100.39 (18.73)	121.09 (11.32)	*t*(53) = -5.10, *p* < .01	116.11 (11.06)	118.37 (9.82)	*t*(74) = -0.91, *p* = .37

### Hearing Status

Click-evoked wave V latencies were reviewed to determine normal hearing status across child and parent groups. Latencies were required to be within two standard deviations of normative click-evoked latencies (Skoe et al., 2015) for inclusion in this study (Table 2).

**Table 2:** Responses to the /da/ and /ja/ stimuli

		ASD Group Mean *(SD)*	ASD Control Group Mean *(SD)*	ASD Parent Group Mean *(SD)*	Parent Control Group Mean *(SD)*
**/**dɑ/	*Wave V*	6.714 (0.313)	6.565 (0.237)	6.683 (0.367)	6.530 (0.222)
	*Wave A*	7.800 (0.386)	7.520 (0.267)	7.800 (0.474)	7.505 (0.354)
	*Wave D*	22.788 (0.537)	22.617 (0.698)	22.865 (0.859)	22.826 (0.665)
	*Wave E*	31.415 (0.629)	30.963 (0.393)	31.232 (0.558)	31.066 (0.541)
	*Wave F*	39.806 (0.673)	39.456 (0.421)	39.726 (0.518)	39.566 (0.631)
	*Wave O*	48.244 (0.493)	48.158 (0.383)	48.318 (1.064)	48.220 (0.386)
	*Prestimulus noise*	0.038 (0.011)	0.031 (0.006)	0.0362 (0.011)	0.036 (0.011)
	*Spectral amplitude (fundamental frequency)*	0.056 (0.019)	0.067 (0.023)	0.048 (0.014)	0.054 (0.018)
	*Spectral amplitude (first formant)*	0.016 (0.004)	0.017 (0.006)	0.015 (0.005)	0.016 (0.005)
	*Response consistency*	0.907 (0.309)	1.352 (0.372)	0.906 (0.420)	1.077 (0.392)
**/**jɑ/	*Pitch strength*	0.374 (0.137)	0.484 (0.168)	0.350 (0.208)	0.388 (0.161)
	*Pitch error*	13.872 (9.500)	10.080 (6.536)	15.591 (9.719)	13.197 (8.397)
	*Correlation coefficient*	0.590 (0.383)	0.712 (0.301)	0.518 (0.348)	0.622 (0.356)

### Electrophysiological Recording

#### Stimuli and Presentation

The stimuli included one 40 ms synthesized speech syllable /dɑ/ and one 230 ms naturally voiced /jɑ/ syllable with an ascending pitch contour (130–220 Hz) applied in Praat (Boersma, 2001). The /dɑ/ and /jɑ/ stimuli were chosen based on the availability of extensive normative data and past findings in individuals with ASD, suggesting that further investigation of these stimuli may be most fruitful for the research questions investigated here. The /dɑ/ stimulus was developed to allow for evaluation of neural response latency, representation of key frequency components in the stimulus (specifically the fundamental frequency and first formant, referred to here as “low” and “mid” frequencies), and response consistency, whereas the /jɑ/ stimulus was developed solely to test neural pitch tracking ability. Stimuli are available upon request.

Each stimulus was presented monaurally to the right ear at 80 dB SPL through insert earphones (ER-3A, Etymotic Research) while participants sat in a comfortable chair in a quiet room. Participants watched a movie of their choice to maintain relaxation for the duration of the FFR collection. Stimuli were presented with alternating polarity in order to minimize stimulus artifacts and to maximize the temporal envelope processing element of the response (Krizman & Kraus, 2019). Stimuli were presented in the following order: /dɑ/ followed by /jɑ/. The /dɑ/ was presented at a rate of 10.9 / sec in two trials of 3000 presentations each. The /jɑ/ was presented at a rate of 3.56 / sec in two trials of 2400 presentations each. Together, the collection of the two stimuli took about 35 min.

#### Recording Parameters

Differential responses were collected using the Bio-logic Navigator Pro (Natus, Inc) AEP system. FFRs using a vertical montage (active Cz, forehead ground, ipsilateral earlobe references) using Ag–AgCl electrodes with impedances less than 5 kOhms. /dɑ/ responses were bandpass filtered from 0.1 to 2 kHz, 12 dB/Octave, sampled at 12 kHz, and averaged online with a time window of −15.8 to 69.45 ms, re stimulus onset. /jɑ/ responses were bandpass filtered from 50 to 1600 Hz, 12 dB/Octave, sampled at 3.2 kHz, and averaged online with a time window of −50 to 269.49 ms. For both stimuli, trials with activity greater than ± 23.8 μV were automatically rejected as artifacts during testing.

### Electrophysiological Response Processing

Averaged waveforms were exported from the Bio-logic Navigator Pro system using AEP2ASCII (Natus, Inc.). Subsequent processing was performed in MATLAB using analysis routines published in the Brainstem Toolbox (https://brainvolts.northwestern.edu/freeware/). The two stimuli were originally developed for different purposes, thereby yielding different measurement variables, described below.

#### /dɑ/ Measurement and Analysis

Prior to analysis, recordings were evaluated for validity using both objective and subjective measurements, including presence of excess prestimulus noise and recording quality. Prestimulus baseline RMS amplitude provides a measurement of subject internal noises prior to stimulus onset. Because there should not be time-locked activity during this period, recordings containing greater than 0.06 μV RMS amplitude during this time region were removed. In addition, a trained rater (author: TN) blind to diagnosis reviewed remaining recordings to provide a rating of recording quality on a 4-point scale (1: worst; 4: best). If a recording received a rating of 1 or 2, recordings from the associated participant were removed prior to analyses. The sample sizes reported in the manuscript reflect the final sample following application of these validation procedures. Remaining subjects’ responses underwent the following analysis procedures: (1) Latencies of stereotypical peaks V, A, D, E, F, and O were determined by visual inspection from the examiner, who was unblinded to diagnostic status. These peaks are labeled in the bottom panel of Fig. 1. (2) Representations of key frequencies in the response were derived by obtaining frequency-specific amplitudes. Specifically, a segment of the response from 19.5 to 44.2 ms, encompassing the response to the voiced portion of the /dɑ/, was windowed with a 2-ms on/2-ms off Hanning ramp, de-meaned, and converted to the frequency domain with a 4096-point Fourier transform. Then, sample frequencies were summed between 75 and 175 Hz (corresponding to fundamental frequency) and 175 and 750 Hz (corresponding to first formant). 3) Response consistency was estimated from the two 3000-trial blocks that were recorded. A response segment from 19.5 to 44.2 ms was extracted from each block and a Pearson’s correlation was computed between them. To obtain a normal distribution, resulting r values were Fisher-transformed (inverse hyperbolic tangent) to z values for subsequent analyses.

#### /jɑ/ Measurement and Analysis

**Fig. 2:**
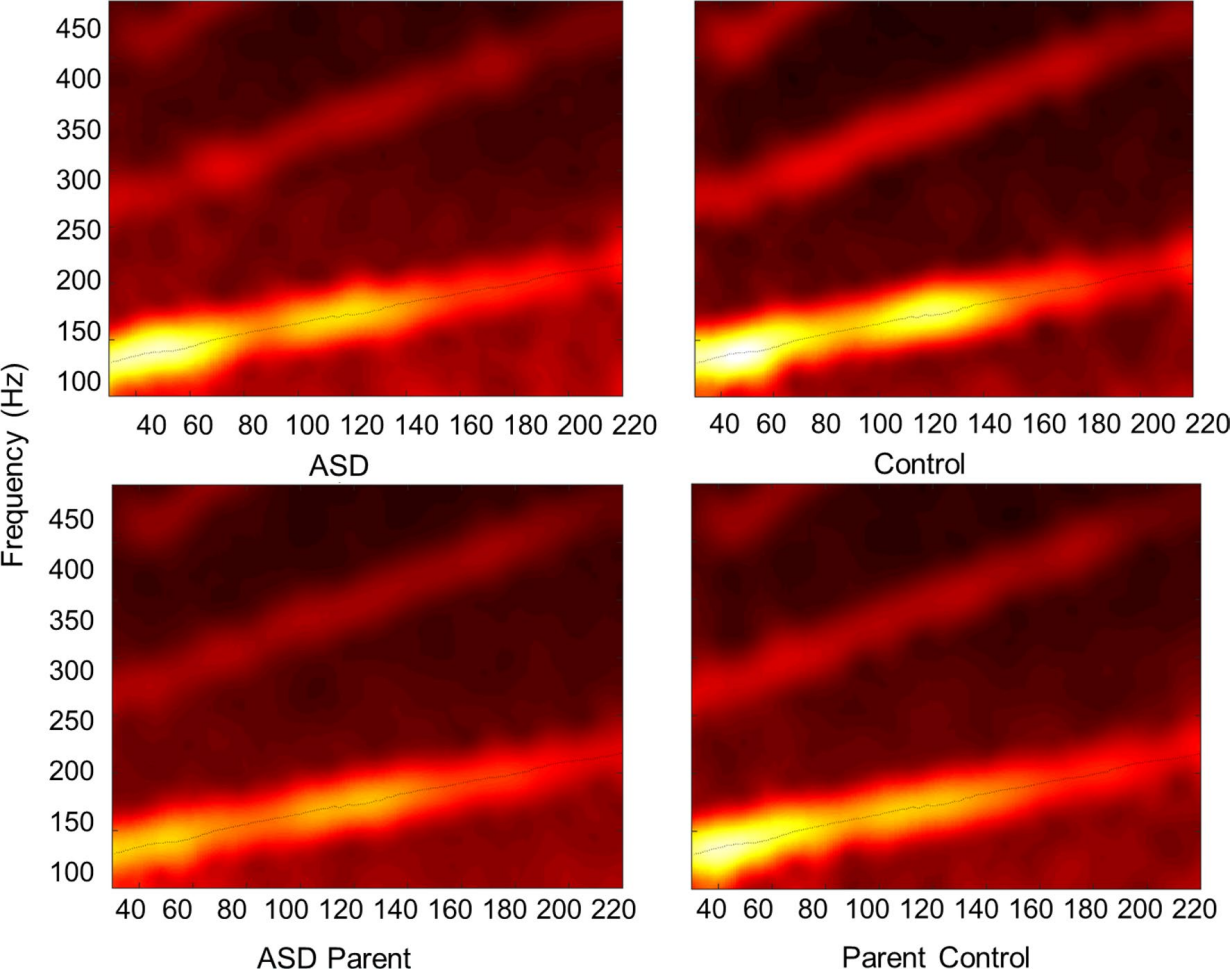
Neural representation of the /jɑ/ speech-stimulus. Lighter and brighter colors represent increased fidelity in neural representation of the pitch features of the stimulus. The black dotted line signifies the pitch of the stimulus

**Fig. 3:**
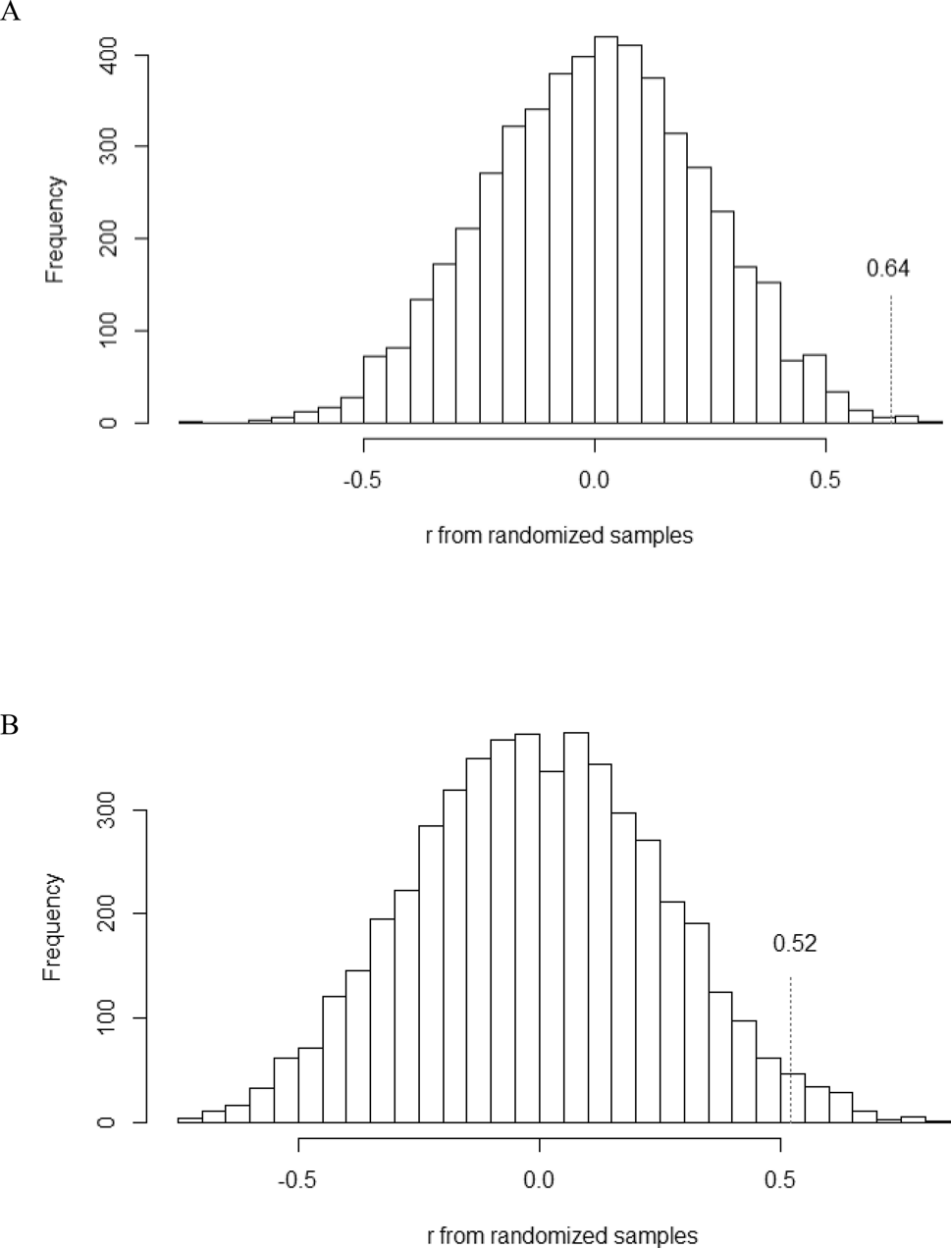
Example of distributions produced from randomization test to examine familiality of FFR in mother and child dyads. Frequency distribution reflects the frequency of obtaining a correlation coefficient value for random mother–child pairings; arrow signifies true correlation coefficient between mother and child dyad. **A** latencies for wave V in control mother–child random pairings, and **B** latencies for wave D in ASD mother–child random pairings

Pitch tracking of the /jɑ/ responses was assessed using short-time autocorrelation and short-time Fourier transform techniques. Both techniques assessed overlapping 40-ms Hanning-windowed time bins, sliding in 1 ms increments. For the STFT technique, the point of maximal spectral energy was determined between 120 and 230 Hz, frequencies that encompassed the rising fundamental frequency of the syllable. Examples of the resulting spectrograms produced by this procedure are shown in Fig. 2. For the short-time autocorrelation, the maximum correlation between a 4.38 ms (14 samples) and 8.44 ms (27 samples) lag, corresponding to 118.5–228.6 Hz, was determined. Identical techniques were used to extract the pitch of the evoking stimulus so that direct comparisons between stimulus and response were possible. Pitch tracking ability is quantified by three measures. (1) Pitch Strength, computed via autocorrelation, indicates the robustness of neural phase locking to voice pitch and is reported as the maximal r-value of the in 4.38–8.44 ms lag range, averaged across all time bins. (2) Pitch Error, computed by STFT, refers to the absolute-value difference in frequency between the maximal spectral energy of the stimulus and the response, and is reported in Hz averaged across all time bins. (3) Correlation Coefficient is a direct Pearson correlation of the instantaneous pitches across the time bins of the stimulus and the participants’ neural response, as determined by STFT (Fig. 3).

### Speech and Language Correlates

#### Pragmatic Language Skills

The Pragmatic Rating Scale-School Age (PRS-SA; Landa, 2011) was used to assess pragmatic language skills in the ASD and ASD Control groups. The PRS-SA is rated from video recordings of semi-structured play and conversation from the ADOS-2 (Lord et al., 2012). In the ASD Parent and Parent Control groups, the Pragmatic Rating Scale (PRS; Landa, 2013) was used to assess pragmatic language skills. The PRS is coded based on a semi-structured conversational interview in which an examiner asks the parent a series of questions about their childhood, schooling, social relationships, and occupation. Both the PRS-SA and PRS comprise different subscales that index similar skills. The PRS-SA subscales include: presupposition (e.g., redundant conversation, inadequate clarification, failure to provide background information); discourse management (e.g., acknowledgement, reciprocal conversation, response elaboration); speech/language behaviors that affect pragmatic language (e.g., overly formal language, scripted language, and language that is difficult to understand); suprasegmentals (e.g., intonation of voice, rate of speech, volume modulation); and nonverbal communication (e.g., use of gestures, eye-contact, and facial expressions). The PRS subscales include: dominant conversation style (e.g., too detailed, tangential); listener expectation (e.g., unable to clarify, failure to reciprocate); and suprasegmentals (e.g., intonation of voice, rate of speech, volume modulation). For both the PRS-SA and the PRS, two coders blind to group independently rated the interactions for pragmatic language features on a three-point scale, with 0 indicating absent, 1 indicating mild, and 2 indicating present. The coders resolved coding discrepancies through discussion in order to reach a consensus.

#### Prosodic Ability

The Profiling Elements of Prosody in Speech-Communication (PEPS-C; Peppé & McCann, 2015) assessed prosodic ability in all participants. The PEPS-C measures receptive and expressive prosody across seven specific skill areas, including the ability to understand and use prosody in a way that communicates a specific function, such as lexical stress or affect, as well as the ability to discriminate and imitate intonation patterns. Each domain of the PEPS-C contained 16 items and participants received one point per correct response.

### Statistical Analysis

Responses were examined using a series of multivariate analyses of covariance (MANCOVA) for the /dɑ/ and /jɑ/ stimuli to assess differences between the ASD and ASD Control groups, as well as the ASD Parent and Parent Control groups, controlling for chronological age. The initial MANCOVA for the /dɑ/ stimulus included all latencies for waves A-O, which reflect the onset and offset of the neural response as well as phase locking to the fundamental frequency. A secondary MANCOVA for the /dɑ/ stimulus was conducted to assess spectral properties of the response and included measures of spectral amplitude (low and mid frequencies that correspond to the fundamental frequency (F0) and the first formant (F1), respectively). Additional univariate analyses of covariance were conducted to assess for group differences in response consistency and prestimulus noise. For the /jɑ/ stimulus, a MANCOVA assessing differences in pitch strength, pitch error, and correlation coefficient was conducted. Planned comparisons were investigated following each MANCOVA (even when the overall model was nonsignificant) to guard against Type 2 error and directly address the study hypothesis that speech/language differences in individuals with ASD, as well as subtle differences among parents of individuals with ASD, arise from inefficient neural processing of speech, As such, we predicted that the ASD group would demonstrate increased neural response latencies and reduced neural pitch tracking abilities compared to controls. We predicted similar patterns would emerge within the parent groups. Effect sizes are provided to support interpretations of results.

Pearson correlations were conducted to explore relationships between FFR variables and pragmatic language and prosodic abilities on the PRS-SA and PRS, and PEPS-C, respectively. Of note, correlations did not withstand a Bonferroni correction but are reported to guide future research in this developing area of study. For the PRS-SA and PRS, specific domain scores were investigated when associations between the FFR variable and total pragmatic language violations were detected. Each of the seven domains of the PEPS-C was examined separately in correlations. Correlations were conducted in the ASD and ASD Control groups combined and the ASD Parent and Parent Control groups combined to investigate associations across the full range of performance for each measure. Familiality of /dɑ/ and /jɑ/ responses were assessed using exploratory mother–child correlations (ASD dyads n = 16; Control dyads n = 19). Father-child correlations were not explored because of a limited number of father-child dyads (ASD dyads n = 4; Control dyads n = 2). To investigate whether mother–child correlations were not a by-product of similar patterns observed at the group level, *Pearson*’s correlations were conducted between unrelated mother–child dyads by applying a randomization test (Katz et al., 1990) to evaluate the specificity of familial relationships. Based on this randomization test, because each parent value was randomly paired with a child’s value per waveform, the expected correlation coefficient was zero. Random pairings were repeated for all possible permutations within each diagnostic group, to generate a sampling distribution of the correlation coefficients. The strength of the true mother–child correlation coefficient was compared against the distribution of all possible permutations of unrelated dyads within diagnostic groups (e.g., true ASD parent–child dyads versus all unrelated ASD pairings and true control parent–child dyads versus all unrelated control pairings), to obtain a probability statistic indicating the likelihood of any random pairing producing a stronger correlation relative to the reported true parent–child correlation coefficient.

## Results

### Group Differences in FFR

#### /dɑ/ Stimulus

*ASD and ASD controls* The overall model assessing latencies between the ASD and ASD Control groups was statistically significant (F = 2.32, p = 0.05, d = 0.41; see Fig. 1), with the ASD group showing significantly greater response latencies for waves A (F = 8.09, p = 0.006, d = 0.76), E (F = 7.76, p = 0.007, d = 0.74), and F (F = 4.56, p = 0.04, d = 0.57), and a marginally greater response latency for wave V (F = 3.63, p = 0.06, d = 0.51). ASD and ASD Control groups did not differ in response latency for wave D (F = 1.29, p = 0.26, d = 0.30) or wave O (F = 0.61, p = 0.44, d = 0.21). The model assessing spectral amplitude between the ASD and ASD Control groups was not significant (F = 2.70, p = 0.08, d = 0.44). The ASD group exhibited significantly greater prestimulus noise (F = 7.77, p = 0.007, d = 0.74) and reduced response consistency (F = 29.62, p < 0.001, d = 1.45) compared to controls.

*ASD Parents and ASD Parent Controls* The overall model comparing latencies between the ASD Parent and Parent Control groups approached statistical significance (F = 2.11, p = 0.06, d = 0.33). The ASD Parent group exhibited significantly greater response latencies for waves V (F = 4.77, p = 0.03, d = 0.50) and A (F = 10.06, p = 0.002, d = 0.73; see Fig. 1). ASD Parent and Parent Control groups did not differ in response latencies for waves D (F = 0.05, p = 0.83, d = 0.05), E (F = 2.14, p = 0.15, d = 0.34), or F (F = 1.52, p = 0.22, d = 0.28), and O (F = 0.27, p = 0.61, d = 0.12). Comparison of spectral amplitude between the ASD Parent and Parent Control groups was not statistically significant (F = 1.58, p = 0.21, d = 0.29), and there were no differences in prestimulus noise (F = 0.01, p = 0.94, d = 0.02). Results revealed marginally poorer response consistency (F = 3.45, p = 0.07, d = 0.43) in the ASD Parent group.

#### /jɑ/ Stimulus

*ASD and ASD Controls* The model assessing pitch tracking in the ASD and ASD Control groups was not statistically significant (F = 2.14, p = 0.11, d = 0.39). Planned comparisons indicated that the ASD group exhibited reduced pitch strength compared to controls (F = 6.16, p = 0.02, d = 0.66). Groups did not differ in pitch error (F = 2.51, p = 0.12, d = 0.42) or correlation coefficient (F = 1.47, p = 0.23, d = 0.32).

*ASD Parents and Parent Controls* The overall model assessing pitch tracking in the ASD Parent and Parent Control groups did not reach statistical significance (F = 0.55, p = 0.65, d = 0.17).

### Speech and Language Correlates of FFR

#### Pragmatic Language in ASD and ASD Control groups

In ASD and ASD Control groups combined, increased pragmatic language violations were associated with increased prestimulus noise (*r* = 0.35, *p* = 0.02), decreased response consistency (*r* = -0.53, *p* < 0.001), increased pitch error (*r* = 0.35, *p* = 0.05), and reduced pitch strength (*r* = -0.45, *p* < 0.01). Each of these FFR variables, except latency for wave E, were associated with increased difficulty with discourse management (e.g., topic initiation, interrupting; *|r|s* > 0.34*, ps* ≤ 0.05). Prestimulus noise, response consistency, and pitch strength were also related to impairments in nonverbal communication (e.g., atypical eye contact, gestures; |*r|s* > 0.36*, ps* < 0.05). Pitch strength was additionally related to increased violations in the speech/language behaviors domain (e.g., overly formal speech; stereotyped utterances; *r* = -0.41*, p* = 0.02). Longer latencies for wave E, as well as reduced response consistency and decreased pitch strength were associated with increased suprasegmental difficulties (e.g., intonation modulation, speech rate; |*r|s* > 0.36, *ps* < 0.05).

#### Pragmatic Language in ASD Parent and Parent Control groups

In the parent groups collapsed, increased pragmatic language violations were associated with decreased spectral amplitude for the fundamental frequency (*r* = − 0.26, *p* = 0.04) and less response consistency (*r* = − 0.26, *p* = 0.04). Associations with spectral amplitude for the fundamental frequency were detected with dominant conversational style (e.g., tangential comments, topic preoccupation; *r* = *− *0.30*, p* = 0.02) and pragmatic language violations related to listener expectations (e.g., fails to reciprocate, vague; *r* = *-*0.27*, p* = 0.04). The relationship between response consistency and pragmatic language violations appeared to be driven by differences in suprasegmentals (*r* = *− *0.35*, p* < 0.01).

#### Prosodic Ability

In the ASD and ASD control groups, associations with receptive prosody skills emerged, with increased neural response latency and reduced response consistency associated with poorer Contrastive Stress. Sporadic associations were observed between measures of response latency, spectral amplitude, and pitch tracking with Turn-End and Boundary understanding. Poorer expressive prosody, particularly in the domains of Imitation, Turn-End, and Boundary expression, was associated with increased neural response latency and reduced response consistency, as well as poorer pitch tracking. Affect, Lexical Stress, and Phrase Stress domains were not associated with FFR (see Table 3).

**Table 3:** Associations with PEPS-C receptive and expressive subtests in ASD and ASD Control groups

	ASD and ASD Control groups													
	Discrimination/ Imitation		Turn-End		Affect		Lexical Stress		Phrase Stress		Boundary		Contrastive Stress	
	Rec	Exp	Rec	Exp	Rec	Exp	Rec	Exp	Rec	Exp	Rec	Exp	Rec	Exp
*Response Latency Wave V*	−0.047	−.488^**^	−0.150	−0.206	−0.104	0.015	−0.147	−0.060	−0.042	−0.172	−0.225	0.004	−0.257	−0.085
*Response Latency Wave A*	0.127	−.477^**^	−0.075	−0.144	−0.084	0.098	−0.118	−0.206	0.036	−0.110	0.019	−0.003	−.333^^^	−0.158
*Response Latency Wave D*	0.324	−0.151	−0.032	0.012	0.172	0.175	0.121	0.144	0.389^^^	0.275	0.184	0.131	−0.038	−0.184
*Response Latency Wave E*	−0.086	−.468^**^	−0.081	−0.268	−0.127	−0.088	−0.261	−0.036	−0.059	0.036	−0.347	−0.128	−.392^*^	−0.102
*Response Latency Wave F*	0.228	−.403^*^	−0.017	−0.089	−0.049	0.099	−0.047	−0.108	0.337	0.111	0.086	0.074	−0.206	−0.223
*Response Latency Wave O*	0.100	−0.012	.426^*^	0.195	0.219	0.053	−0.053	0.023	0.173	0.022	0.119	−0.449^*^	0.135	0.176
*Prestimulus Noise*	−0.058	−0.183	−0.186	−0.270	−0.040	0.028	−0.036	−0.161	−0.017	−0.115	0.126	0.421^^^	−0.104	−0.030
*Response Consistency*	0.216	.348^*^	0.282^^^	.384^*^	0.071	−0.192	0.063	0.207	0.176	0.225	0.136	−0.056	.410^**^	0.000
*Spectral Amplitude (low)*	0.053	0.287^^^	.338^*^	0.196	−0.082	−0.267	−0.168	−0.041	−0.086	−0.092	0.021	−0.242	0.208	0.222
*Spectral Amplitude (mid)*	0.111	0.075	.317^^^	0.215	−0.099	−0.010	−0.169	−0.091	−0.127	−0.118	0.017	−0.348	0.164	0.064
*/ja/ Pitch Error*	0.161	−0.056	0.277	−.293	0.178	−0.182	0.100	−0.045	0.117	0.101	0.240	0.073	−0.223	−0.011
*/ja/ Pitch Strength*	−0.038	0.087	−.401^*^	.335^^^	−0.261	0.169	−0.221	−0.108	−0.100	0.015	−.549^*^	−0.343	0.107	−0.272
*/ja/ Correlation Coefficient*	−0.391^^^	0.131	−0.279	0.097	−0.296	0.086	−0.178	−0.144	−0.297	−0.310	−0.182	−0.099	0.110	0.197

Significant correlations are indicated in bold-face text

**denotes *p* < .01

*denotes* p* < .05

^ denotes *p* < .10

Similar to patterns identified in ASD and ASD Control groups, in parent groups, poorer receptive prosody skills in the domain of Contrastive Stress were associated with increased neural response latencies and reduced response consistency, as well as reduced spectral amplitude of the fundamental frequency. Poorer expressive prosody skills in the domain of Contrastive Stress were associated with reduced spectral amplitude of the fundamental frequency. Sporadic associations between Phrase Stress and Boundary expression and neural response latencies emerged. Similar to findings in the ASD and ASD Control groups, several domains of prosody were not related to FFR (see Table 4).

**Table 4:** Associations with PEPS-C receptive and expressive subtests in Parent groups

	*Discrimination/ Imitation*		*Turn–End*		*Affect*		*Lexical Stress*		*Phrase Stress*		*Boundary*		*Contrastive Stress*	
	*Rec*	*Exp*	*Rec*	*Exp*	*Rec*	*Exp*	*Rec*	*Exp*	*Rec*	*Exp*	*Rec*	*Exp*	*Rec*	*Exp*
*Response Latency Wave V*	–0.212	–0.207	0.013	–0.113	–0.181	–0.012	–0.255	0.135	–0.150	0.366^^^	0.145	–0.294	–0.186	–0.195
*Response Latency Wave A*	–0.075	–0.239	–0.010	–0.221	–0.033	–0.101	–0.025	0.042	–0.025	0.189	0.168	–.368^*^	–0.187	–0.103
*Response Latency Wave D*	–0.062	–0.036	0.064	–0.014	0.083	–0.026	–0.117	0.114	0.031	.440^*^	0.182	0.135	–.324^*^	0.010
*Response Latency Wave E*	–0.082	–0.247	0.057	–0.113	0.004	0.025	–0.159	0.057	–0.128	0.145	–0.011	–0.021	–.309^*^	–0.135
*Response Latency Wave F*	–0.022	–0.057	0.155	0.054	0.088	–0.043	0.019	0.183	0.120	0.271	0.058	0.160	–0.110	0.076
*Response Latency Wave O*	–0.207	0.127	–0.022	0.120	–0.054	0.021	0.118	–0.032	0.210	0.184	–0.185	.408^*^	–0.090	0.230
*Prestimulus Noise*	0.234	–0.162	0.092	–0.076	0.123	0.210	0.099	0.110	–0.121	–0.030	0.073	–0.052	0.106	0.065
*Response Consistency*	–0.173	0.254	0.021	0.025	0.004	–0.111	0.154	0.002	0.097	–0.085	0.001	0.103	.339^*^	0.120
*Spectral Amplitude (low)*	–0.053	0.179	–0.028	–0.106	0.073	0.191	0.135	–0.029	0.030	–0.153	–0.146	0.104	.310^*^	.348^*^
*Spectral Amplitude (mid)*	–0.093	0.003	0.020	–0.015	0.213	–0.013	0.053	0.083	–0.262	–0.162	–0.209	–0.130	0.166	0.125
*/ja/ Pitch Error*	0.036	–0.147	0.126	0.075	0.042	0.270	–0.093	0.041	–0.116	0.138	–0.075	0.018	–0.256	–0.161
*/ja/ Pitch Strength*	0.014	0.211	–0.318^^^	0.057	0.070	–0.137	0.083	–0.039	0.136	0.008	0.02	0.056	0.296^^^	0.199
*/ja/ Correlation Coefficient*	–0.025	–0.084	–0.063	–0.286^^^	0.063	–0.268	–0.029	–0.067	–0.023	–0.097	–0.041	0.081	0.077	0.169

Significant correlations are indicated in bold-face text

*denotes* p* < .05

^ denotes *p* < .10

#### Familiality of FFR

For mother–child ASD dyads, response latencies for wave D (*r* = 0.52, *p* = 0.04, probability r_true_ > r_random_ = 97.8%; i.e., the likelihood that the correlation coefficient derived from the mother–child dyad correlation (r_true_) is greater than the correlation coefficients derived from all permutations of the unrelated parent–child pairs (r_random_) is 97.8%) and prestimulus noise (*r* = 0.52, *p* = 0.04, probability r_true_ > r_random_ = 97.7%) were positively correlated. Further, negative correlations emerged for pitch error (*r* = −0.72, *p* < 0.01, probability r_true_ < r_random_ = 99.8%; i.e. the likelihood that r_true_ is less than r_random_ suggests that r_true_ is stronger than random pairs 99.8% of the time, in the case of a negative correlation) and pitch strength (*r* = −0.72, *p* < 0.01, probability r_true_ < r_random_ = 99.4%).

In mother–child Control dyads, responses latencies for waves V (*r* = 0.64, *p* < 0.01, probability r_true_ > than r_random_ = 99.8%) and A (*r* = 0.63 *p* < 0.01, probability r_true_ > than r_random_ = 99.8%) were positively associated. Additionally, spectral amplitude for the first formant frequency was positively associated (*r* = 0.60, *p* < 0.01, probability r_true_ > than r_random_ = 99.7%).

## Discussion

This study examined FFR as a potential heritable, neural mechanism contributing to the ASD language phenotype, and its more subtle expression in relatives, who are at increased genetic liability to ASD. Consistent with hypotheses, both the ASD and ASD Parent groups showed diminished FFR to complex speech sounds, with more pervasive differences evident in the ASD group. Indices of poorer FFR along several key variables were related to elevated pragmatic language differences, and poorer expressive prosody skills across groups. Evidence of a unique pattern of familiality of FFR was also detected in ASD families. Together, these findings point towards disruptions in neural processing of speech sounds as a heritable neurobiological mechanism in ASD that may contribute to the complex ASD language profile.

Importantly, findings revealed overlapping FFR differences in prestimulus noise and response latencies among individuals with ASD and their parents, suggesting that altered temporal processing of speech sounds is influenced by genetic predisposition to ASD. Results revealed delayed onset of neural processing of the speech syllable /dɑ/, in both individuals with ASD and their parents. The ASD group also exhibited increased neural response latencies for waves representing the acoustic properties of the stimulus, suggesting decreased phase locking to the stimulus frequency. These delays indicate increased neural conduction time in both individuals with ASD and their parents, which may be a byproduct of increased prestimulus noise, which can influence sensory encoding (Iemi et al., 2019; McNair et al., 2019; Samaha et al., 2017; Samaha & Postle, 2015). Indeed, prior work has shown that children affected by linguistic deprivation exhibit greater neural noise. By contrast, collegiate athletes demonstrate an improved ability to minimize neural noise to more clearly tune into the speech signal, further highlighting neural noise as a potentially key indicator of general auditory neural acuity (Krizman et al., 2020; Skoe et al., 2013). Increased prestimulus noise, as observed here, may be reflective of hyperexcitability in the ASD groups, which has been observed across neural regions, and may be linked to inefficient processing of sensory stimuli (see Takarae & Sweeney, 2017 for review). This may have downstream implications related to receptive, expressive, and pragmatic language as observed in this study. Furthermore, decreased response consistency in the ASD group suggests greater variability in speech sound representations. Such disruptions are believed to play a role in impaired phonological development (e.g., formation and use of speech sounds) in individuals with reading difficulties (Hornickel & Kraus, 2013), and could contribute to inefficient neural processing observed in ASD.

Consistent with prior findings demonstrating reduced pitch tracking in individuals with ASD (Russo, Larson, et al., 2008; Russo, Trommer, et al., 2008), results indicated reduced pitch strength to the /ja/ stimulus, but not increased pitch error, in the ASD group. The lack of differences in pitch processing among ASD parents is not necessarily surprising, given that parents do not show clinical impairments. Rather, the attenuated FFR differences in parents may be notable in implicating a refined constellation of neural processing abilities specifically influenced by ASD genetic liability (namely, temporal and spectral processing) and not encumbered by the influence of comorbidities or multiply impaired symptom domains typical in ASD. Significant parent–child associations in temporal processing of speech sounds emerged and were compared against random permutations for confidence in interpretation. These results provided strong evidence of a familial relationship in the FFR responses between mothers and children across groups, on top of group level differences in neural responses to speech sounds. These results are consistent with prior work showing matrilineal patterns of transmission for language-related impairments in ASD, mother–child associations in gaze-language coordination in language processing tasks (Nayar et al., 2018) and evidence that elevated polygenic risk for ASD is related to increased pragmatic language differences in mothers (Nayar et al., 2020). Findings here highlight specific FFR components that might constitute potent, heritable markers of neural differences related to the language profile in ASD and broader language phenotypes among first degree-relatives.

Evidence implicating atypical FFR as a neural mechanism contributing to the ASD language phenotype is further supported by associations between FFR and pragmatic language and prosodic skills across groups. Specifically, significant, parallel relationships across the ASD, ASD Parent, and control groups were detected between neural response timing and pitch representations and increased pragmatic language violations, particularly in the area of suprasegmentals. Given that pragmatic impairment is a defining feature of ASD (Baltaxe & Simmons, 1977; Landa, 2000; Losh et al., 2012; Peppé et al., 2006; Tager-Flusberg et al., 2011), and subclinical pragmatic language differences have been repeatedly documented in ASD relatives (Landa et al., 1992; Losh et al., 2008; Patel et al., 2019, 2020; Piven et al., 1997), associations between pragmatics and neural processing of speech sounds are significant in implicating FFR as neural mechanism related to a core symptom domain in ASD. Associations between response latencies and key pragmatic language skills in parents are also intriguing in suggesting that even subtle differences in neural processing of sound might have reverberating effects on downstream, more complex language abilities, such as pragmatics, which rely on the integration of many foundational mechanisms and skills. This is consistent with prior evidence of relationships between neural response latencies and magnitudes with overall cognitive and language abilities (Russo, Larson, et al., 2008; Russo, Trommer, et al., 2008), and implicates FFR as a potentially important target for study in understanding the complex brain basis of pragmatic language impairments that characterize ASD and their subclinical manifestation in relatives.

Relationships between FFR and prosodic abilities were also detected, but in less clear or robust patterns than in pragmatics. Poorer contrastive stress understanding was consistently related to increased neural response latencies and reduced response consistency across groups, suggesting that receptive prosody is an important aspect of communication impacted by inefficient neural processing of speech sounds. Furthermore, relationships between poorer temporal processing and pitch tracking, as well as reduced response consistency, and increased expressive prosodic errors in the ASD and ASD Control groups may reflect a greater impact of inefficient neural processing of speech sounds on prosodic production. However, FFR associations with other prosodic skills were not clearly evident. In parents, this is perhaps unsurprising, considering the near-ceiling effects on the PEPS-C demonstrated by parent groups. Inconsistent findings more generally may have related to specific features of the task. For instance, receptive prosody tasks were not timed, so the counterintuitive relationships that emerged (e.g., reduced pitch strength associated with greater turn-end understanding) could reflect more effortful processing, which yielded successful behavioral responses on the PEPS-C despite delayed neural processing of speech sounds detected in the ASD and ASD Control groups. Inconsistent findings could also constitute false discovery resulting from multiple tests conducted in our effort to uncover potential relationships between FFR and prosodic skills. Relationships that did emerge were of medium effect sizes and thus provide some preliminary evidence of a link between temporal and spectral processing at the neural level and clinically meaningful deficits in prosody that will be important to investigate further with larger samples, and a wider array of more tightly constrained prosodic assessments that more sensitively tap prosodic variability across clinically affected and unaffected groups.

## Limitations

Several limitations should be considered in interpreting results. First, the study’s focus on verbally fluent individuals with ASD, while important for reducing comorbidities, may limit generalization of findings to clinically affected individuals with lower levels of language abilities. Therefore, it will be important for future research to investigate whether differences in FFR, and relationships between FFR and pragmatic language and prosody, extend to individuals with more severe language and/or cognitive impairments. Likewise, the age range of individuals with ASD did not span the lifespan, so it is unclear if the FFR may serve as a biomarker early in development as well as later in adulthood. There is cross-sectional evidence to suggest that the FFR changes across the lifespan, so it is possible group differences may be less or more robust at certain times in development. Finally, familiality of FFR was only assessed with mother–child dyads because of the limited number of fathers. Some evidence indicates differences between maternal vs. paternal traits and ASD symptomatology in their children, suggesting potential differences in familiality between mothers and fathers (Klusek et al., 2014; Maxwell et al., 2013; Nayar et al., 2018), making it important to further study patterns of lineality to investigate the inheritance of FFR and provide insights into gene-brain-behavior connections.

## Conclusions

This study identified converging evidence of differences in FFR in ASD and parents, familiality of FFR, and associations between FFR and pragmatic and prosodic abilities. These findings add to the understanding of neurobiological contributions to speech and language deficits characteristic of ASD and implicate the FFR as a potentially heritable neurobiological marker of language-related deficits in ASD. Moreover, parallel findings in parents of individuals with ASD, as well as associations with language abilities, suggest that the FFR is impacted across the spectrum of genetic vulnerability to ASD, including relatives who do not exhibit clinical impairments. Given existing research demonstrating the experience-related malleability of neural responses to speech (Chandrasekaran & Kraus, 2010; Fujioka et al., 2004; Kraus et al., 2014; Song et al., 2008; Wong et al., 2007), the relationships detected between the FFR and pragmatic language and prosody in the present work may also support the study of FFRs to speech sounds as a sensitive, biological index of response to speech and language intervention.

## Supplementary Information

Below is the link to the electronic supplementary material.

Supplementary file1 (M 9 kb)

Supplementary file2 (M 33 kb)

Supplementary file3 (M 16 kb)

Supplementary file4 (AVG 6 kb)

Supplementary file5 (AVG 4 kb)

Supplementary file6 (AVG 141 kb)

Supplementary file7 (M 2 kb)

Supplementary file8 (M 2 kb)

Supplementary file9 (M 4 kb)

Supplementary file10 (M 2 kb)

Supplementary file11 (M 2 kb)

Supplementary file12 (M 2 kb)

Supplementary file13 (M 3 kb)

Supplementary file14 (FIG 12 kb)

Supplementary file15 (FIG 8 kb)

Supplementary file16 (FIG 24 kb)

Supplementary file17 (FIG 12 kb)

Supplementary file18 (FIG 23 kb)

Supplementary file19 (M 3 kb)

Supplementary file20 (M 3 kb)

Supplementary file21 (M 2 kb)

Supplementary file22 (FIG 7 kb)

Supplementary file23 (FIG 23 kb)

Supplementary file24 (FIG 14 kb)

Supplementary file25 (M 1 kb)

Supplementary file26 (M 1 kb)

Supplementary file27 (M 1 kb)

Supplementary file28 (M 9 kb)

Supplementary file29 (M 0 kb)

Supplementary file30 (M 2 kb)

Supplementary file31 (M 2 kb)

Supplementary file32 (M 8 kb)

Supplementary file33 (M 0 kb)

Supplementary file34 (M 0 kb)

Supplementary file35 (AVG 14 kb)

Supplementary file36 (AVG 6 kb)

Supplementary file37 (AVG 4 kb)

Supplementary file38 (M 19 kb)

Supplementary file39 (M 17 kb)

Supplementary file40 (M 1 kb)

Supplementary file41 (M 0 kb)

Supplementary file42 (M 0 kb)

Supplementary file43 (M 3 kb)

Supplementary file44 (AVG 203 kb)

Supplementary file45 (M 0 kb)

Supplementary file46 (M 3 kb)

Supplementary file47 (M 0 kb)

Supplementary file48 (M 0 kb)

Supplementary file49 (M 1 kb)

Supplementary file50 (M 11 kb)

Supplementary file51 (M 4 kb)

Supplementary file52 (M 3 kb)

Supplementary file53 (DLL 20 kb)

Supplementary file54 (DLL 7 kb)

Supplementary file55 (M 0 kb)

Supplementary file56 (M 17 kb)

Supplementary file57 (M 14 kb)

Supplementary file58 (PDF 42 kb)

Supplementary file59 (PDF 364 kb)

Supplementary file60 (PDF 615 kb)

Supplementary file61 (PDF 163 kb)

Supplementary file62 (PDF 2940 kb)

Supplementary file63 (PDF 326 kb)

Supplementary file64 (PDF 366 kb)

Supplementary file65 (PDF 292 kb)

Supplementary file66 (DOCX 15 kb)

Supplementary file67 (PDF 96 kb)

Supplementary file68 (XLSX 8 kb)

Supplementary file69 (TXT 0 kb)

Supplementary file70 (PNG 154 kb)

Supplementary file71 (TXT 5 kb)

Supplementary file72 (M 2 kb)

Supplementary file73 (M 2 kb)

Supplementary file74 (M 7 kb)

Supplementary file75 (M 14 kb)

Supplementary file76 (DOCX 15 kb)

## Abbreviations

ASD: Autism spectrum disorder
FFR: Frequency following response
ADOS-2: Autism Diagnostic Observation Schedule, 2^nd^ Edition
PRS-SA: Pragmatic Rating Scale-School Age
PRS: Pragmatic Rating Scale
PEPS-C: Profiling Elements of Prosody in Speech-Communication
