# Supplementary material for: Neural Processing of Speech Sounds in ASD and First-Degree Relatives
Source: J Autism Dev Disord. 2022 Jun 7;53(8):3257–71. doi: 10.1007/s10803-022-05562-7 (PMC10019095; doi:10.1007/s10803-022-05562-7)
Supplement: Supplementary file 58 — Supplementary file58 (PDF 42 kb) [file 10803_2022_5562_MOESM58_ESM.pdf]

bt_ascii2avg	this function takes an ascii file and converts contents to<br>1) a Neuroscan-format .avg file, and<br>2) a text file containing the marked latencies<br>Both resulting files will retain the same base filename as the ASCII file with extensions of .avg and .mrk, respectively.<br><br>Usage: bt_ascii2avg('filename.txt')
bt_biologic2avg	this function takes a Bio-logic ascii file and converts its contents to:<br>1) a Neuroscan-format .avg file, and<br>2) a text file containing the marked latencies<br>Both resulting files will retain the same base filename as the ASCII file with extensions of .avg and .mrk, respectively.<br><br>Usage: bt_biologic2avg('filename.txt')
bt_fft2	this function computes frequency-domain amplitudes of three user-defined frequency bins of brainstem response. Results are not scaled to peak μV.<br><br>Usage: [F0 F1 HF] = bt_fft2('filename.avg',10,40,100,150,300,350,600,800);<br><br>over the range of 10 to 40 ms, finds average frequency amplitude of 100-150 Hz, 300-350 Hz and 600-800 Hz.<br><br>Three variables are returned the workspace:<br>(1) Freq1: mean amplitude over F0_Lo-F0_Hi Hz<br>(2) Freq2: mean amplitude over F1_Lo-F1_Hi Hz<br>(3) Freq3: mean amplitude over HF_Lo to HF_Hi Hz
bt_fftbiomap	this function computes frequency-domain amplitudes of F0, F1 and high-freqency bins of BioMAP response.<br><br>Usage: [F0 F1 HF] = bt_fftbiomap('filename.avg',start_latency,stop_latency);<br><br>Note, BioMAP default latencies are 11.38, 40.58.<br><br>Three variables are returned the workspace:<br>(1) F0: mean amplitude over F0_Lo-F0_Hi Hz<br>(2) F1: mean amplitude over F1_Lo-F1_Hi Hz<br>(3) HF: mean amplitude over HF_Lo to HF_Hi Hz

bt_fftsc	this function computes frequency-domain amplitudes of three user-defined frequency bins of Brainstem response. Results are scaled to peak μV.<br><br>Usage: [F0 F1 HF] = bt_fftsc('filename.avg',10,40,100,150,300,350,600,800);<br><br>over the range of 10 to 40 ms, finds average frequency amplitude of 100-150 Hz, 300-350 Hz and 600-800 Hz.<br><br>Three variables are returned the workspace:<br>(1) Freq1: mean amplitude over F0_Lo-F0_Hi Hz<br>(2) Freq2: mean amplitude over F1_Lo-F1_Hi Hz<br>(3) Freq3: mean amplitude over HF_Lo to HF_Hi Hz
bt_qncorr	this function compares two files via correlation. Typically this is used to compare a response recorded in background noise with a response to the same stimulus in quiet.<br><br>Usage: [StraightCorr CrossCorr Lag]=bt_qncorr(Qfile, Nfile, start, stop);<br><br>Requires 4 input arguments:<br>Qfile, Nfile: quiet and noise .avg files.<br>start, stop: latency, in ms., over which the correlations are run<br><br>3 variables are returned to the workspace:<br>(1) StraightCorr: Simple Pearson's r over desired latency range<br>(2) CrossCorr: Maximum correlation, allowing noise response to lag quiet by up to 2 ms.<br>(3) Lag: Amount of timeshift (in ms.) to achieve CrossCorr<br><br>In cases where the best correlation occurs with no shift, Lag will be 0 and StraightCorr will equal CrossCorr.<br><br>Dependancies:<br>openavg.m<br>xcorrelation.m

bt_rms	this function computes RMS amplitude of response period (selectable), of prestim period, and the resulting SNR.<br><br>Usage: [RMS RMSprestim SNR] = bt_rms('filename.avg',start_latency,stop_latency);<br><br>Note, if start,stop omitted, BioMARK default latencies (11.38, 40.58) are used.<br><br>Three variables returned to workspace are<br>RMS: RMS amplitude of selected latency range<br>RMSprestim: RMS amplitude of prestimulus activity<br>SNR: The quotient of RMS/RMSprestim
bt_srcorr	Compares brainstem file to the evoking stimulus using cross correlation<br><br>Usage: [PosCorr PosLag NegCorr NegLag] = bt_srcorr('file.avg', start, stop);<br><br>A typical brainstem response, when cross-correlated with the stimulus, exhibits two major correlogram maxima: one positive, one negative. This function returns both and their respective lags.<br><br>INPUT ARGUMENTS:<br>'file.avg': BioMARK data file (in .avg format)<br>start, stop: latency, in ms., over which the correlation is run<br><br>OUTPUT ARGUMENTS:<br>PosCorr, NegCorr: maximum/minimum r-values found at lags corresponding to onset response.<br><br>PosLag, NegLag: lags corresponding to above.<br><br>Dependancies:<br>filtered_stimulus.avg<br>openavg.m<br>xcorrelation.m
bt_xcorrelation	this function calculates the maximum correlation (maxmincor) value and its respective lag (LAG) over an imputted lag range, for a specified portion of a response. The user must specify whether to find the max positive or max negative correlation value
