# Supplementary material for: Neural Processing of Speech Sounds in ASD and First-Degree Relatives
Source: J Autism Dev Disord. 2022 Jun 7;53(8):3257–71. doi: 10.1007/s10803-022-05562-7 (PMC10019095; doi:10.1007/s10803-022-05562-7)
Supplement: Supplementary file 59 — Supplementary file59 (PDF 364 kb) [file 10803_2022_5562_MOESM59_ESM.pdf]

Plasticity in the Adult Human Auditory Brainstem following Short-term Linguistic Training

Judy H. Song, Erika Skoe, Patrick C. M. Wong, and Nina Kraus

Abstract

■ Peripheral and central structures along the auditory pathway contribute to speech processing and learning. However, because speech requires the use of functionally and acoustically complex sounds which necessitates high sensory and cognitive demands, long-term exposure and experience using these sounds is often attributed to the neocortex with little emphasis placed on subcortical structures. The present study examines changes in the auditory brainstem, specifically the frequency following response (FFR), as native English-speaking adults learn to incorporate foreign speech sounds (lexical pitch patterns) in word identification. The FFR presumably originates from the auditory midbrain and can be elicited preattentively. We measured FFRs to the trained pitch patterns before and after training. Measures of pitch tracking were then derived from the FFR signals. We found increased accuracy in pitch tracking after training, including a decrease in the number of pitch-tracking errors and a refinement in the energy devoted to encoding pitch. Most interestingly, this change in pitch-tracking accuracy only occurred in the most acoustically complex pitch contour (dipping contour), which is also the least familiar to our English-speaking subjects. These results not only demonstrate the contribution of the brainstem in language learning and its plasticity in adulthood but also demonstrate the specificity of this contribution (i.e., changes in encoding only occurs in specific, least familiar stimuli, not all stimuli). Our findings complement existing data showing cortical changes after second-language learning, and are consistent with models suggesting that brainstem changes resulting from perceptual learning are most apparent when acuity in encoding is most needed. ■

Uncorrected Proof

INTRODUCTION

Before entering the neocortex, successive neural impulses initiated by sounds entering the cochlea reach subcortical structures, including the brainstem. As acoustic and/or functional complexity of sound increases, the more likely higher-level structures will be involved in processing (Gordon & O'Neill, 2000; Suga, Gao, Zhang, Ma, & Olsen, 2000). Specifically, the processing and plasticity of the encoding of speech sounds is generally attributed to the neocortex (Liebenthal, Binder, Spitzer, Possing, & Medler, 2005; Zatorre, Evans, Meyer, & Gjedde, 1992), although brainstem and thalamic structures contribute to such processing to a certain extent. Evidence of such high-level (including cognitive) involvement and learning-associated plasticity comes from studies of long-term and short-term perceptual learning of speech (Tervaniemi et al., 2006; Näätänen et al., 1997; Tremblay, Kraus, Carrell, & McGee, 1997; Kraus et al., 1995).

There is a growing body of evidence to suggest that subcortical structures contribute actively to auditory processing and are not simply passive relay stations transmitting information from the peripheral sensory organs to the cortex. Recent studies have shown that the auditory brainstem can be modified by short-term and long-term auditory experiences initiated in childhood. For example, Russo, Nicol, Zecker, Hayes, and Kraus (2005) found improved auditory brainstem timing to speech stimuli in background noise in children with language-based learning problems following an 8-week commercially available auditory speech training program. Krishnan, Xu, Gandour, and Cariani (2005) measured the impact of long-term language experience on the frequency following response (FFR). They found that native Mandarin-speaking subjects with at least 20 years of Mandarin language exposure (beginning in childhood) showed more precise linguistic pitch pattern encoding relative to native English-speaking subjects. Mandarin Chinese, a tone language, uses pitch, the psychological correlate of F0, to signal word meaning at the syllable level (e.g., /ma/ spoken with high-level and rising pitch patterns means "mother" and "numb," respectively).[1] This increased neural precision reflects Mandarin-speakers' long-term learning of Mandarin tones (lexically meaningful pitch patterns), and how this experience has changed the response properties of subcortical neurons for enhanced processing of linguistic pitch patterns. Similarly, long-term musical training initiated in childhood has been shown to enhance frequency encoding in the brainstem, such that English-speaking musicians who did not speak Mandarin

Northwestern University, Evanston, IL

© 2008 Massachusetts Institute of Technology

showed more robust and faithful encoding of Mandarin tones, especially the dipping (i.e., falling then rising) contour (Wong, Skoe, Russo, Dees, & Kraus, 2007). Enhancements due to life-long musical training have also been found in the brainstem encoding of acoustically transient events and harmonic content for both speech and music (Musacchia, Sams, Skoe, & Kraus, 2007). It is important to note that all of these studies involve auditory experiences initiated in childhood. It is yet to be demonstrated whether short-term experiences occurring in adulthood can have any measurable impact on the brainstem.

The present study examines changes in the auditory brainstem, specifically the FFR, as native English-speaking adults learn to incorporate foreign speech sounds (lexical pitch patterns) in word identification. The FFR, a far-field potential recorded from surface electrodes, reflects the synchronized activities of axonal and dendritic potentials generated by populations of neurons in the lateral lemniscus and/or inferior colliculus of the brainstem (Hoormann, Falkenstein, Hohnsbein, & Blanke, 1992; Smith, Marsh, & Brown, 1975) and is well suited for examining how speech-specific pitch contours are encoded subcortically. There is a vast literature demonstrating the existence of a temporal code of pitch encoding at the level of the auditory nerve and the brainstem (Moller, 1999; Langner, 1997). This temporal code is observed in discharge patterns of single neurons and in synchronous population-wide neuronal activity. The pattern of neural discharge (phase-locking) is modulated by the temporal structure of the eliciting sound. In the temporal structure of speech sounds, periodic amplitude modulations reflect the rate of the F0 and elicit the perception of pitch. This periodicity is maintained in the neuronal representation, with interspike intervals entraining to the period of the F0 and its harmonics. The acoustic features of the evoking stimulus, both spectral and temporal, are represented with high fidelity in the FFR, making it possible to compare the response frequency composition and timing to the corresponding features of the stimulus (Johnson, Nicol, & Kraus, 2005; Kraus & Nicol, 2005; Galbraith et al., 2000; Hall, 1979).

We trained native English-speaking adults to use three different pitch patterns (or tones): high-level, rising, and dipping pitch patterns in word identification. For example, subjects learned that the pseudoword ''pesh'' spoken with a high-level, rising, and dipping tone meant ''glass,'' ''pencil,'' and ''table,'' respectively. These tonal patterns resemble those used lexically in Mandarin Chinese. High-level and rising tones are acoustically simpler than dipping tones (Figure 1) and are used frequently in English as intonational (nonlexical) markers at the syllable level. The dipping tone, which contains a large downward then upward excursion, can only occur at the phrase level in English (Pierrehumbert, 1979) and is the most difficult tone for second-language learners to master (Gottfried & Suiter, 1997; Kiriloff, 1969).

The FFR was elicited preattentively to the three training tones superimposed onto an untrained syllable

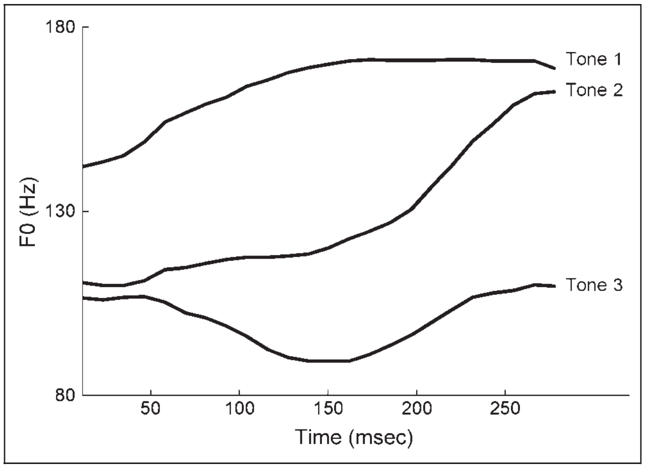

Figure 1. F0 contours of the high-level (Tone 1), rising (Tone 2), and dipping/falling–rising (Tone 3) patterns extracted from the /mi/ stimuli used for physiologic recording (F0 ranges: 140–172 Hz, 110–163 Hz, and 89–110 Hz, respectively).

(/mi/). Tone 3, which was the least familiar to the subjects, served as the experimental stimulus, whereas Tone 1 and Tone 2, which occur in English, served as within-subject control stimuli. Subjects' neural pitch-tracking accuracy to the stimulus F0 was assessed before and after they were trained on our lexical pitch task. If short-term linguistic learning can result in brainstem plasticity even in adulthood, we would expect pitch-tracking accuracy to improve. This improvement would be most evident in the dipping tone which is the most acoustically complex (as its shape involves a falling–rising pattern) and is the least familiar to the subjects.

METHODS

Subjects

Twenty-three (14 women) native English-speaking adults (age = 19–40 years; mean age = 26.3 ± 5 years) participated in this study. All subjects reported no audiologic or neurologic deficits and had normal click-evoked auditory brainstem response latencies with right ear stimuli presentation at 80 dB SPL (Hood, 1998). Hearing thresholds were screened at 20 dB HL for octaves from 500 to 4000 Hz for both ears. All subjects had normal IQ (mean IQ = 119 ± 13.6), as measured by Wechsler's Abbreviated Scale of Intelligence (WASI) (Wechsler, 1999). Informed consent was obtained from all subjects. The Institutional Review Board of Northwestern University approved this research study.

Training

Stimuli

Mandarin is a tonal language that utilizes changes in pitch to indicate word meaning. In this study, a male native speaker of Mandarin Chinese produced the syllable

Uncorrected Proof

/mi/ with three Mandarin tones: high-level (Tone 1), rising (Tone 2), and dipping (Tone 3) (Figure 1). The F0 contours of the training and electrophysiological stimuli were both created based on these three speech tokens. For the training study, the F0 contours from the three syllables were extracted (Figure 1) and superimposed onto six English pseudowords, to form six minimal triads using the Pitch-Synchronous Overlap and Add (PSOLA) resynthesis method implemented and documented in PRAAT (Boersman & Weeknick, 2005). The pseudowords follow English phonotactic rules words but are not part of the English lexicon. The six monosyllabic pseudowords (i.e., ''dree,'' ''fute,'' ''ner,'' ''nuck,'' ''pesh,'' and ''vece''; formal phonetic transcriptions are provided in Table 1) were produced by a male native speaker of American English in a sound-attenuated chamber via a SHURE SM58 microphone onto a Pentium IV PC sampled at 44.1 kHz. English pseudowords were used because unknown words containing native phonological patterns are easier to learn than those with nonnative phonological patterns (Feldman & Healy, 1998). The F0 contour was duration normalized to fit the length of each originally produced pseudoword. In other words, although the pitch trajectory was identical in the spectral domain for all pseudowords using the same tone (i.e., ''pesh1,'' ''dree1,'' ''ner1,'' etc.), the rate of frequency modulation (how frequency change over time) was different depending on the duration of the originally produced pseudoword. All stimuli were amplitude normalized such that, after resynthesis, each originally produced pseudoword had three variants that differed only in F0 with the duration, syllable onset, rhyme, and coda being identical. These 18 (6 × 3) resynthesized stimuli, with pitch contours from a male Mandarin speaker and syllables from an American English speaker, were used in the training program. Six native Mandarin-speaking adults judged the training stimuli to be perceptually natural; each identified the pitch patterns of the training stimuli with at least 95% accuracy.

Procedures

The training program lasted eight sessions. Each session, including the training blocks, practice quizzes, and test, lasted approximately 30 min. All subjects completed training in 14 consecutive days, with no more than 2 days between sessions. These training stimuli and procedures were adapted from our previous study (Wong & Perrachione, 2007; Wong, Perrachione, & Parrish, 2007). Subjects were trained to associate pseudowords with drawings that represented high-frequency English nouns. In order to facilitate learning, the 18 pseudowords were split into minimal contrast triads (six groups of three stimuli). The training session was divided into six blocks, and the subject was trained on one triad per block, similar to our previous study. The order of the blocks was randomized across training sessions. Training involved the simultaneous presentation of the sound of the pseudoword via headphones and corresponding picture. Within each block, subjects were presented each sound–picture pair four times resulting in a total of 72 (6 blocks × 4 times × 3 pitch contours) pseudoword–picture presentations during each daily training session. At the end of each block, subjects were given a practice quiz which required them to match the pseudoword with one of three drawings. Subjects received feedback on their performance—if the correct picture was identified, ''Correct'' was displayed on the computer screen, and ''Incorrect. The correct answer is ...⟨correct picture shown⟩'' was displayed if the wrong picture was chosen. After one block was completed, the next block began immediately and this was repeated until all six blocks were completed.

After completing the six blocks, subjects were tested on the entire set of 18 pseudowords without feedback. This test presented each pseudoword one at a time, randomized and repeated three times (total of 54 trials). Subjects were instructed to identify each word by selecting the corresponding drawing out of 18 possible choices. Subjects were allowed to take as much time as needed to associate word and picture. The word identification score was obtained at the end of each session in order to monitor the subjects' progress.

Physiologic Responses to Pitch Patterns (Tones)

Physiologic Stimuli

The same three tokens of the syllable /mi/ containing the three Mandarin tones [high-level (Tone 1), rising (Tone 2), and dipping (Tone 3)] were also used to generate stimuli for the FFR. These utterances were duration-normalized to 278.5 msec, resulting in stimuli which contained the same pitch trajectories in the spectral

Table 1. Training Stimuli

p^hɛŝ1 ''glass''	dri1 ''arm''	nɛr1 ''boat''	vɛs1 ''hat''	n∧k1 ''brush''	fjut1 ''shoe''
p^hɛŝ2 ''pencil''	dri2 ''phone''	nɛr2 ''potato''	vɛs2 ''tape''	n∧k2 ''tissue''	fjut2 ''book''
p^hɛŝ3 ''table''	dri3 ''cow''	nɛr3 ''dog''	vɛs3 ''piano''	n∧k3 ''bus''	fjut3 ''knife''

Subjects were trained on a vocabulary of 18 pseudowords. Each word, written in the International Phonetic Alphabet, is followed by its corresponding meaning in quotes. Numbers following lexical items designate tone. High-level tone is indicated by 1, rising tone by 2, and dipping tone by 3, according to convention (adapted from Wong & Perrachione, 2007).

Uncorrected Proof

domain as the training stimuli but differed in the rate of frequency modulation. F0 contours from each syllable were then extracted and superimposed onto the original /mi1/ syllable, following resynthesis procedures described above. This resulted in three stimuli which, other than differing in F0, were acoustically identical and were judged to be perceptually natural by four native speakers of Mandarin. In Mandarin, the syllable /mi/ spoken with these tones translates ''to squint,'' ''to bewilder,'' ''rice,'' respectively. The minimum and maximum frequencies of F0 contours of the three stimuli were 140–172 Hz, 110–163 Hz, and 89–110 Hz, respectively (Figure 1). These stimuli were RMS amplitude normalized using the software Level 16 (Trice & Carrell, 1998). To accommodate the capabilities of our stimulus presentation software, the stimuli were resampled to 22.05 kHz. These stimuli are identical to those in our previous study (Wong, Skoe, et al., 2007) and were not used in training.

Physiologic Recording Procedures

During the pre- and posttraining sessions, subjects watched a videotape with the sound level set at less than 40 dB SPL to facilitate a quiet yet wakeful state. Subjects' left ears were unoccluded to allow for the delivery of the video soundtrack, while the stimuli were presented to the right ear at ~70 dB SPL (Neuroscan Stim; Compumedics, El Paso, TX) through insert ear phones (ER-3; Etymotic Research, Elk Grove Village, IL). The order of the three stimuli was randomized across subjects with a variable interstimulus interval between 71.50 and 104.84 msec. Responses were collected using Scan 4.3 (Neuroscan; Compumedics) with three Ag–AgCl scalp electrodes, differentially recorded from Cz (active) to the ipsilateral earlobe (reference), with the forehead as ground. Contact impedance was less than 5 kΩ for all electrodes. Two blocks of 1200 sweeps per block were collected at each polarity with a sampling rate of 20 kHz. Filtering, artifact rejection, and averaging were performed off-line using Scan 4.3. Responses were band-pass filtered from 80 to1000 Hz, 12 dB/octave, and trials with activity greater than ±35 μV were considered artifacts and rejected. Waveforms were averaged with a time window spanning 45 msec prior to the onset and 16.5 msec after the offset of the stimulus. Responses of alternating polarity were then added together to isolate the neural response by minimizing stimulus artifact and cochlear microphonic (Gorga, Abbas, & Worthington, 1985). For the purpose of calculating signal-to-noise ratios (SNRs), a single waveform representing non-stimulus-evoked neural activity was created by averaging the neural activity 40 msec prior to stimulus onset.

Analysis Procedures

In order to assess training-induced physiologic changes, we measured subjects' FFRs elicited by the three trained pitch patterns (tones) embedded in the untrained syllable /mi/ (Figure 1 shows the F0 contours of these stimuli). Physiologic data were collected immediately before the first session of training and immediately after the last session of training. For each subject, three measures of FFR pitch tracking were calculated: *pitch-tracking error*, *spectral dominance of F0*, and *pitch noise ratio*, which were used to assess the subjects' pitch tracking to the stimulus F0 contours of the three stimuli. These measures were derived using a sliding window analysis procedure, in which 40-msec bins of the FFR were analyzed in the frequency domain. The FFR was assumed to encompass the entire response beginning at time 1.1 msec, the transmission delay between the ER-3 transducer and ear insert. The 40-msec sliding window was shifted in 1-msec steps to produce a total of 238 overlapping FFR bins. A narrow-band spectrogram was calculated for each Hanning-windowed FFR bin by applying the Fast Fourier Transform (FFT). To increase spectral resolution, each time bin was zero-padded to 1 sec before performing the FFT. The spectrogram gave an estimate of spectral energy over time and the F0 (pitch) contour was extracted from the spectrogram by finding the spectral peak closest to the expected (stimulus) frequency. F0 frequency and amplitude were recorded for each time bin. The same short-term spectral analysis procedure was applied to the stimulus waveforms. In Figures 4 and 5, the time indicated on the x-axis refers to the midpoint of each 40-msec time bin analyzed. *Pitch-tracking error* was calculated using both linear and logarithmic scales. *Linear pitch-tracking error*, a measure of pitch encoding accuracy over the duration of the stimulus, was calculated by finding the absolute Euclidian distance between the stimulus F0 and response F0 at each time bin and averaging the error across all 238 bins. In computing *logarithmic pitch-tracking error*, the stimulus F0 and the response F0 were transformed to log units before finding the average absolute difference between the stimulus and the response.

Spectral dominance of F0 and *pitch noise ratio* are two measures of spectral amplitude, which consider the extent to which the extracted F0s meet or surpass a specific threshold across the entire stimulus. Specifically, *spectral dominance of F0* describes whether the extracted F0s were at the spectral maximum and reflects the strength of F0 encoding for the duration of the stimulus in the brainstem. This measure of spectral amplitude was calculated by finding the number of time bins in which the extracted F0 fell at the spectral maximum (largest peak in spectrum). For each time bin, an SNR was also calculated. This was done by applying an FFT to the 40-msec waveform representing the non-stimulus-evoked activity (*noise*) and then finding the spectral amplitude corresponding to F0 that was extracted from the respective FFR bin. SNRs were calculated as $\mathrm{F0Amplitude}_{\mathrm{FFR\ BIN}x}/\mathrm{F0Amplitude}_{\mathrm{NOISE}}$, where x is a number from 1 to 238, and F0 is the frequency

Uncorrected Proof

extracted from bin x. Pitch noise ratio describes whether the extracted F0s were above the noise floor, and thus, represents the number of bins for which the SNR was greater than one. It reflects the magnitude of the response in encoding stimulus F0 relative to the ongoing neural response. All pitch-tracking analyses were performed using routines coded in Matlab 7.0.4 (The Mathworks, Natick, MA).

RESULTS

Behavioral Measures (Tone Training Program)

Subjects' word identification performance was evaluated at the end of each training session. We found that immediately after the first training session, subjects' mean word identification was 21.56% (range = 4.17% to 58.33%). At the end of the last training session (henceforth "attainment"), subjects' mean performance was 89.49% (range = 11.11% to 100%), an improvement of 67.93%, which is statistically significant as revealed by a paired t test ($t = -17.06$, $p < .0001$). Figure 2 shows subjects' mean learning trajectory.

Physiologic Responses (Pitch Pattern/Tone Stimuli)

We hypothesized that learning-induced brainstem modifications would only occur for Tone 3 (dipping tone), thus we first report results concerning Tone 3. For Tone 3 linear pitch-tracking error, a one-way repeated measures analysis of variance (ANOVA) revealed a main effect of training [$F(1, 22) = 14.343$, $p = .001$] (Figure 3A). Thus, subjects' brainstem response exhibited more faithful representation of the dipping stimulus F0 contour after being trained to use this tone in a lexical context.

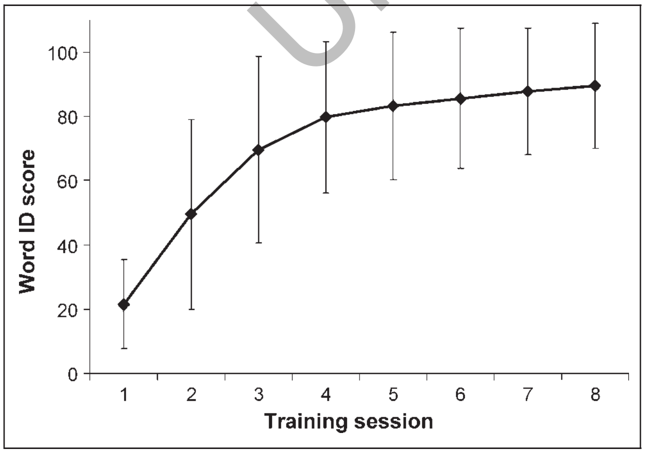

Figure 2. Mean word identification scores. Subjects' mean word identification scores measured at the end of each day of training indicating learning progress. Error bars show one standard deviation from the mean.

Figure 4 shows examples of pitch tracking of the brainstem response to Tone 3 before and after training from three representative subjects (A) as well as word identification scores from their first and last training sessions (B). The same pattern of results was observed for logarithmic pitch-tracking error [$F(1, 22) = 12.897$, $p = .002$]. Moreover, not only was the F0 of Tone 3 encoded more precisely after training, the manner in which it was encoded also changed. After training, F0 was more likely to be encoded by the largest spectral peak as indicated by an increase in spectral dominance in the posttraining data. A one-way repeated measures ANOVA on Tone 3 spectral dominance showed a main effect of training [$F(1, 22) = 4.878$, $p = .038$]. Likewise, pitch noise ratio also increased with training, which resulted in fewer points below the noise floor. A one-way repeated measures ANOVA on Tone 3 pitch noise ratio also showed a main effect of training [$F(1, 22) = 4.454$, $p = .046$]. Figure 5 shows a representative subject's FFR waveforms, FFR spectra, and pitch tracking for the three tones after training, along with the corresponding values for linear pitch-tracking error, spectral dominance, and pitch noise ratio.[2]

As predicted, FFR responses to Tones 1 and 2, the control pitch stimuli did not show measurable changes after training (Figure 3A). One-way repeated measures ANOVAs on Tones 1 and 2 showed no significant changes after training for linear pitch-tracking error [$F(1, 22) = 0.014$, $p = .907$ and $F(1, 22) = 0.477$, $p = .497$, respectively], spectral dominance [$F(1, 22) = 0.034$, $p = .855$ and $F(1, 22) = 0.126$, $p = .726$, respectively], and pitch noise ratio [$F(1, 22) = 0.001$, $p = .980$ and $F(1, 22) = 0.165$, $p = .688$, respectively]. In addition to the absence of mean differences, changes in brainstem responses for Tones 1 and 2 were also more variable compared to Tone 3 (Figure 3B and C). For most of the subjects, the percentage of pitch-tracking errors decreased after training for Tone 3, whereas for Tones 1 and 2, no consistent pattern of change was observed. The fact that brainstem improvement of pitch tracking occurred only with Tone 3 suggests that the impact of short-term linguistic training on subcortical circuitry is highly specific and most evident in the aspect of speech (in this case, tonal pattern) that is least familiar to the learners.

DISCUSSION

Our results demonstrate plasticity in the adult human auditory brainstem following short-term linguistic training. We measured changes in auditory brainstem encoding of variable F0 (pitch) patterns by examining the FFR, a subcortical response presumably originating from the rostral brainstem that encodes the F0 (a physiological correlate of perceived pitch) of the stimulus with high fidelity (Marsh & Worden, 1968). We found that after

Uncorrected Proof

Figure 3. Pre- and posttraining linear pitch-tracking error for Tones 1, 2, and 3. (A) Average pre- and posttraining linear pitch-tracking error for each tone (±1 *SD*). Note that the higher the bar, the larger the deviation from the stimulus contour in Hertz (Hz). *$p < .0001$. (B) Distribution of post- minus pretraining linear pitch-tracking error for individual subjects (pretraining is plotted at zero, posttraining is plotted as the difference between the pre- and posttraining linear pitch-tracking error values). In comparison to Tone 3, Tone 1 and Tone 2 show greater variability. (C) Dot plots of the individual linear pitch-tracking error values before (black squares) and after training (white squares) with a vertical line connecting linear pitch-tracking error values pre- and posttraining for each subject.

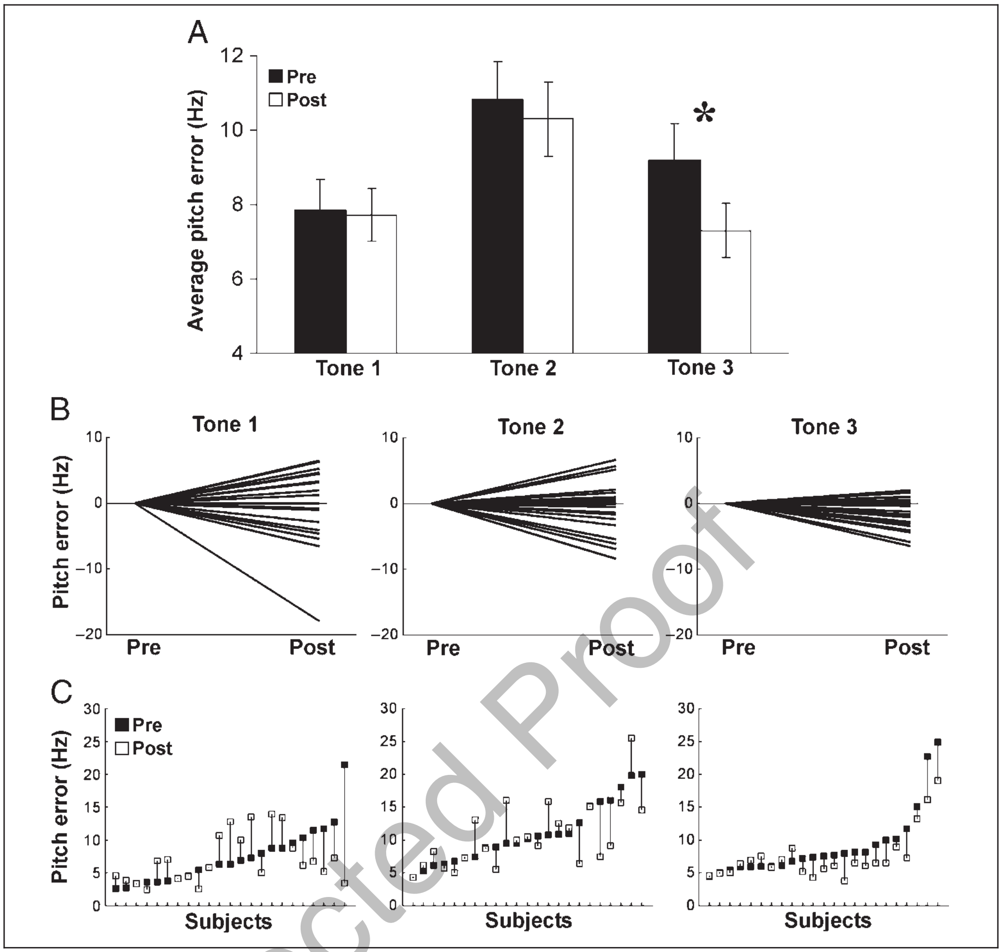

Uncorrected Proof

learning to use three pitch patterns for word identification at the syllable level, native English-speaking adults showed increased accuracy in pitch tracking. It is important to point out that these native English-speaking adults had no experience using pitch lexically prior to our training program. This increase was revealed by a decrease in the number of pitch-tracking errors and a refinement in the energy devoted to encoding pitch, including increased SNRs and increased spectral dominance of the stimulus pitch. Although our subjects had experience using high-level and rising pitch contours at the syllable level for signaling intonation in English, they had less experience with the dipping tone. We found that pitch-tracking improvements occurred only in this least familiar pitch pattern.

Although Krishnan et al. (2005) found that native Mandarin speakers had increased accuracy in pitch tracking compared to native English-speaking adults, and Musacchia et al. (2007) and Wong, Skoe, et al. (2007) found enhanced brainstem encoding of the F0 in musicians, these studies can only speak to the effect of long-term auditory experiences initiated in childhood. Moreover, although short-term training has been shown to improve brainstem timing in children with learning problems (Russo et al., 2005), these findings had not yet been extended to adults. To the best of our knowledge, we are the first to show that experiences acquired in adulthood can modify brainstem auditory responses in a context-specific way.

Our study adds to a growing body of research focusing on the neural encoding of linguistic pitch contours. Taken together, findings from these studies are consistent with a fundamental operating principle of brainstem function, specifically that pitch is represented in a temporal code (phase-locking). This is underscored by the fact that our subjects showed combined improvements in the behavioral and neurophysiological representation of pitch likely reflecting *enhanced* synchronization of neuronal firing to the stimulus F0. This enhancement may be the result of additional neurons firing at the rate of the stimulus F0, the same population of neurons firing more synchronously, or a combination of the two. Moreover, learning may also engender the synchronization or enhancement of populations of neurons which synchronize to the F0 as evidenced by electroencephalogram (Musacchia et al., 2007; Bao, Chang, Woods, & Merzenich, 2004; Shahin, Bosnyak, Trainor, & Roberts, 2003; Tremblay, Kraus, McGee, Ponton, & Otis, 2001) and magnetoencephalogram studies (Shahin, Roberts, Pantev, Aziz, & Picton, 2007; Fujioka, Ross, Kakigi,

Pantev, & Trainor, 2006), both measures reflecting the summation of electrical activity generated by synchronous firing of neurons. In addition to the strengthening of the temporal code, our results may reflect an increase in accuracy of representation via the place code, as the frequencies of our stimuli were low enough to be associated with both temporal and place code.

Our findings can be interpreted within the framework of corticofugal tuning. Although we know that top–down control from the auditory cortex to peripheral auditory receptors can occur in human adults via electrical stimulation of the cortex and through selective attention on perceptual tasks, active training-induced corticofugal modulation has not been shown. In the present study, our subjects received spoken language training through the mapping of new sound structures and lexical–semantic concepts. Their substantial behavioral improvements suggest that they were actively engaged in linguistic learning. The training program involved both high cognitive demands (as it is a case of spoken language learning) and auditory acuity. Thus, it not only engaged the neocortex, but also subcortical structures, respectively. It is conceivable that feedback from the higher-level cortex is initiated so that precise pitch information can be relayed to the neocortex to successfully perform the cognitively demanding task. This comports with models of perceptual learning involving changes in the weighting of perceptual dimensions as a result of feedback (Nosofsky, 1986). Applied to our current study, these models would suggest that the

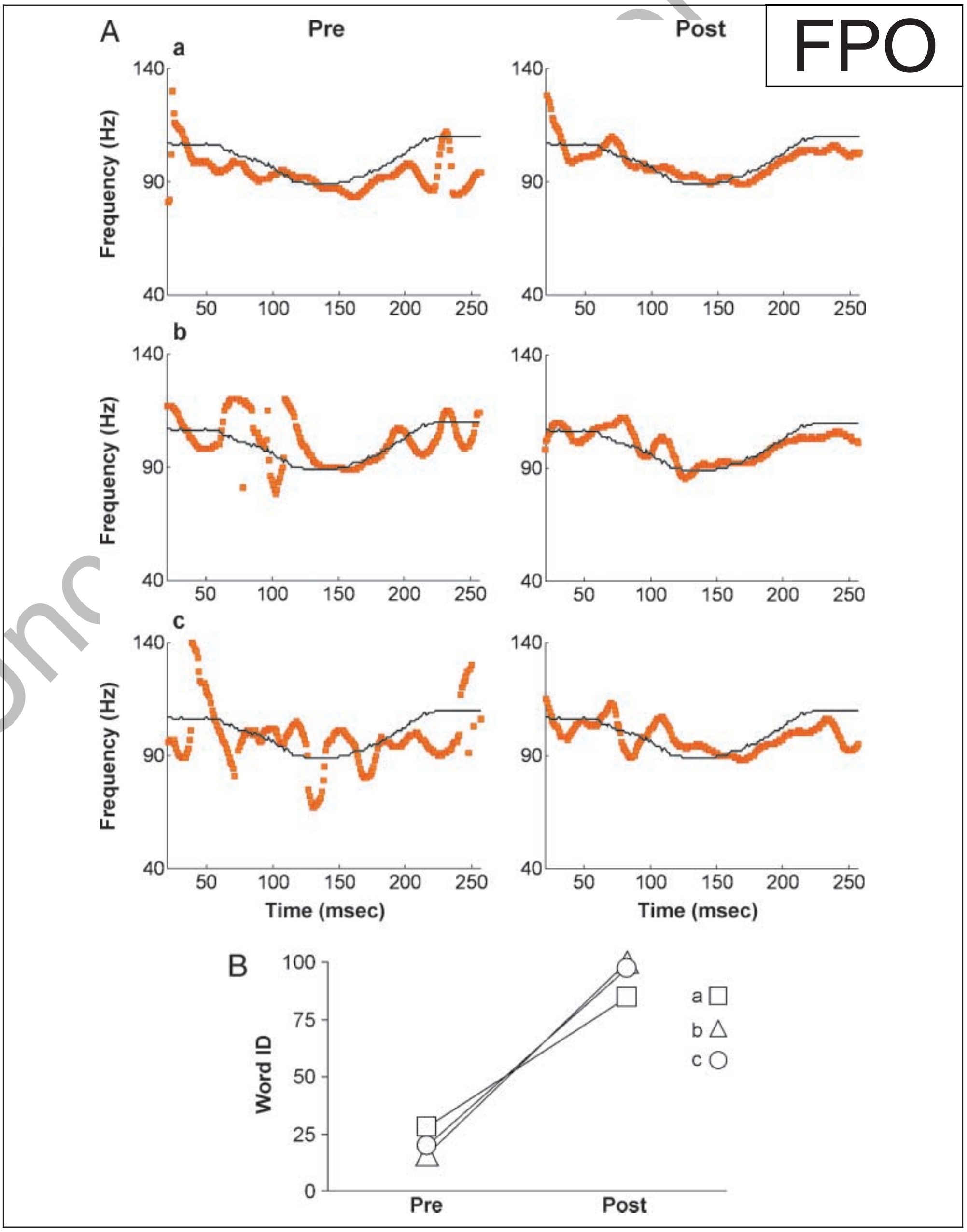

Figure 4. Representative examples of pre- and posttraining pitch-tracking plots. (A) Trajectories (orange line) of brainstem pitch tracking elicited by a dipping pitch contour (Tone 3) for three subjects before and after training. The black line indicates the stimulus (expected) F0 contour. The time indicated on the *x*-axis refers to the midpoint of each 40-msec time bin analyzed. (B) Word identification scores of the first and last training sessions for the subjects plotted in A.

FPO

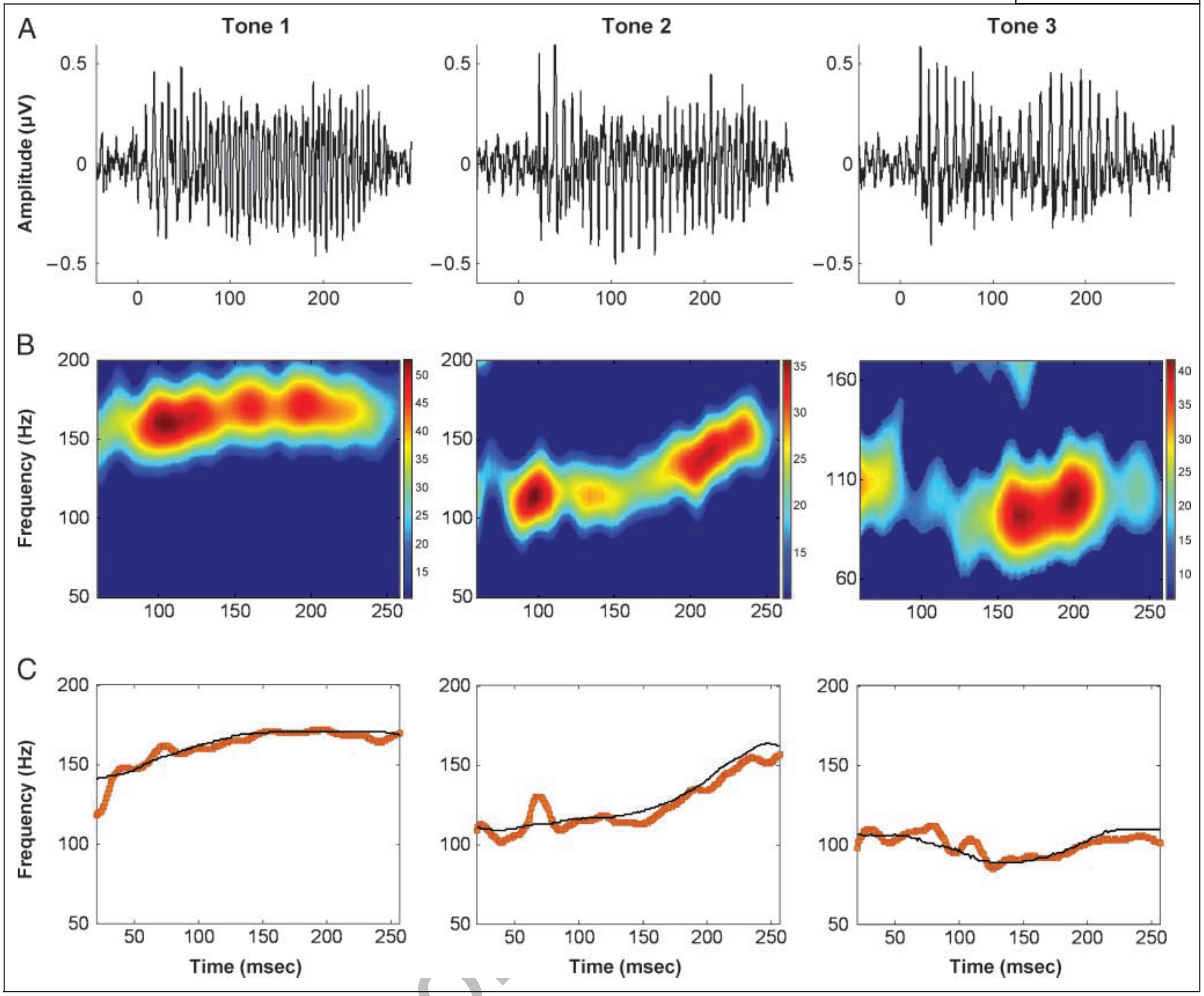

Figure 5. FFR waveforms, spectrograms, and pitch-tracking plots for Tones 1, 2, and 3 for a representative subject. (A) FFR waveforms, (B) FFR spectra, and (C) pitch-tracking plots with stimulus F0 contour (black) and response F0 contour (red) for Tones 1, 2, and 3 after training for a representative subject as a function of time referring to the midpoint of each 40-msec time bin analyzed. In the FFR spectra, color represents FFR spectral amplitude (arbitrary units). The stimulus F0 contours of the high-level (Tone 1), rising (Tone 2), and dipping/falling–rising (Tone 3) range between 140–172 Hz, 110–163 Hz, and 89–110 Hz, respectively. This subject's pre- and posttraining linear pitch-tracking error values are 2.58 and 4.77 (Tone 1), 14.74 and 6.61 (Tone 2), and 8.07 and 4.27 (Tone 3); spectral dominance values are 0 and 4 (Tone 1), 50 and 0 (Tone 2), and 2 and 0 (Tone 3); and pitch noise ratio is 0 for both pre and post responses for all three tones. This subject is plotted in Figure 4 as Subject b.

attentional weighting of pitch-relevant dimensions increased as the result of a training task requiring attention to pitch. Perceptual weighting adjustments were also observed in a recent animal study which showed that visual learning in the presence of irrelevant auditory signals dampened sensitivity to the auditory signals (Delano, Elgueda, Hamame, & Robles, 2007). This line of reasoning is also consistent with the Reverse Hierarchy Theory (RHT) of visual learning suggesting that learning consists of an attention-driven, task-dependent ''backward'' search for increased SNR, especially in highly skilled performers and perceptual experts (Ahissar & Hochstein, 2004).

Relevant to RHT, we found brainstem plasticity to be associated with the most acoustically complex tone, which happened to also be the tone that was least familiar to the subjects. Although our subjects did not have experience using any pitch patterns lexically, as native English speakers, they did have experience using high-level and rising tones at the syllable level for contrasting intonational meaning. As such, they have experience encoding these two pitch patterns. However, the dipping tone is only used at the phrase level in English (e.g., ''You should go home now, MY DEAR FRIEND [the last three words are said in a dipping contour]''). Therefore, our learners had little experience

encoding this tone as rapidly as required at the syllable level. Per RHT, lower-level neural involvements are especially pronounced in more experienced learners and in conditions in which encoding acuity is most needed. Our learners as a group reached above 89% accuracy (most above 90%) and can be argued to be quite experienced on this task. Furthermore, the dipping tone, being most acoustically complex with its falling–rising pattern, arguably most requires the acoustic acuity. Thus, it is not surprising that improvements in brainstem encoding occurred only with this tone. The finding that training effects were only seen for this most complex and previously less familiar tone also suggest a complexity threshold that the cortex needs in order to drive and reinforce subcortical tuning. This complexity threshold relates to both linguistic (Tone 3 being the least familiar tone linguistically) and acoustic (Tone 3 having the most complex pitch contour) complexity. Additional experiments are needed to determine whether both linguistic and acoustic complexities are needed to produce the largest tuning effect in the brainstem.

Interestingly, in our recent study using the same stimulus set, we examined the pitch-tracking accuracy of native English-speaking adult musicians and nonmusicians who had no previous exposure to a tone language. We found that the two groups were best differentiated by their tracking of this dipping tone. More specifically, musicians exhibited better tracking to Tone 3 (but not Tone 1) than nonmusicians (Wong, Skoe, et al., 2007), suggesting that long-term musical experience may give particular advantage to brainstem encoding of complex linguistic pitch patterns. This advantage occurred despite the fact that these particular pitch patterns were not linked specifically to musical training. Note that this seemingly context-general tuning effect (music affecting speech) could be attributed to auditory experience initiated in childhood given that our musician subjects began their musical training early in life. In contrast, it is possible that for experiences initiated in adulthood, such as those in the present study, that brainstem tuning is entirely context-specific and therefore restricted to the (novel) training stimuli. Furthermore, the length of learning can influence the degree of retention. Retention may be impeded following short-term training in adults due to the strong activation of established neural circuitry hampering the acquisition of alternative patterns of circuit connectivity (Knudsen, 2004). Thus, in order to compete with entrenched circuitry and facilitate plasticity, effective attentional shifts and repeated presentations of the content are required (Bahrick & Hall, 2004; Lively, Logan, & Pisoni, 1993).

It is worth noting that we are not ruling out possible passive, bottom–up learning to account for the results. Passive exposure, simple perceptual learning, and sensitivity to stimulus statistical distributions have been found to be associated with behavioral improvements (Seitz & Watanabe, 2003; Maye, Werker, & Gerken, 2002; Watanabe, Nanez, & Sasaki, 2001) and brainstem encoding (Dean, Harper, & McAlpine, 2005; Escabi, Miller, Read, & Schreiner, 2003). Furthermore, neurons in the rostral brainstem have been found to be sensitive to pitch trajectories independent of functional contexts (Gordon & O'Neill, 2000). However, similar to Ahissar and Hochstein, we believe top–down influence to dominate the lower-level (in our case, brainstem) responses given evidence suggesting that practice and active performance, rather than passive learning or generalization, tend to dominate neural changes (Recanzone, Jenkins, Hradek, & Merzenich, 1992). All subjects in the current study were required to actively perform the task and all showed substantial behavioral improvements. It is unlikely that passive exposure to the sounds would result in the same effect we observed (Schoups, Vogels, Qian, & Orban, 2001), especially given that only the least familiar pitch contour showed pitch-tracking improvements. It is worth pointing out that Tone 2 (rising tone) was the tone that showed the most pitch-tracking errors before training. The lack of improvement in this tone, therefore, cannot be attributed to a pretraining ceiling effect. This finding suggests that perceptual learning is an unlikely candidate for explaining the patterns of results we observed.

An important aspect of our results is that although improvement in encoding only occurred with one tone (Tone 3), behavioral improvements demonstrated by word identification were substantial. Our learning paradigm focuses on the matching of 18 auditory stimuli to 18 pictures. The correct use of segmental (consonants and vowels) information alone will likely account for some of the improvement. Furthermore, strengthening the sensitivity of one category in a three-category perceptual space can result in all three categories being more distinguishable as demonstrated by models of perceptual learning (see Fahle & Poggio, 2002 for a review). We believe that, in fact, this is what we observed in the current study. Furthermore, as discussed, the fact that only Tone 3, the tone that is least familiar to the subjects in its linguistic (high-level) usage, resulted in brainstem encoding changes, provides strong evidence for a top–down explanation, although a simple perceptual learning mechanism cannot be ruled out without further experimentation.

We have established that brainstem modifications can occur after short-term training even when it is initiated in adulthood. It is worth noting that although the speech training literature at large has shown that the most efficacious training paradigm (measured by subjects' ability to generalize to new talkers) is one that uses variable stimuli (Clopper & Pisoni, 2004; Lively et al., 1993), a low-variability program (with training stimuli spoken by one talker) was used. As the aim of the current study was to establish, for the first time, a training-induced brainstem effect in adults, we have chosen to adopt a simpler training paradigm. Future work could include high-variability paradigms and examine their

Uncorrected Proof

effect on brainstem plasticity. Future work could also consider brainstem plasticity resulting from different types of complex auditory learning (e.g., music) initiated both in adulthood and childhood to examine any possible quantitative and qualitative differences. This work would inevitably have an impact on education and clinical (re)habilitation (e.g., to inform strategies for improving auditory processing in the rapidly growing population of hearing impaired adults) and would assist in informing social and educational policies.

Acknowledgments

We thank Ann Bradlow, Trent Nicol, and Tyler Perrachione for their assistance in this research. This work was supported by Northwestern University and the National Institutes of Health grants R03HD051827 and R21DC007468 to P. W., and R01DC001510 to N. K., as well as National Science Foundation grant BCS-0544846 to N. K.

Reprint requests should be sent to Patrick C. M. Wong, Communication Sciences & Disorders, Northwestern University, 2240 Campus Drive, Evanston, IL, or via e-mail: pwong@northwestern.edu.

Notes

1. The use of pitch to distinguish word meaning in a tone language is similar to the use of consonants to contrast word meaning in English (e.g., ''pet'' and ''bet'' only differ in the initial consonant and resulted in two different words in English).
2. We also performed correlational analyses between behavioral performance (accuracy in word identification posttraining) and the various physiologic measures. When all subjects were combined, no significant correlations were found. However, seven subjects performed at ceiling behaviorally (100% accuracy in word identification). When those seven subjects were excluded, posttraining behavioral performance was significantly correlated with posttraining Tone 3 linear pitch tracking error (Spearman's rho = .55, p = .014) and log pitch tracking error (Spearman's rho = .61, p = .006).

REFERENCES

Ahissar, M., & Hochstein, S. (2004). The reverse hierarchy theory of visual perceptual learning. *Trends in Cognitive Sciences, 8,* 457–464.

Bahrick, H., & Hall, L. (2004). The important of retrieval failures to long-term retention: A metacognitive explanation of the spacing effect. *Journal of Memory and Language, 52,* 566–577.

Bao, S., Chang, E., Woods, J., & Merzenich, M. (2004). Temporal plasticity in the primary auditory cortex induced by operant perceptual learning. *Nature Neuroscience, 7,* 974–881.

Boersman, P., & Weeknick, D. (2005). *PRAAT: Doing phonetics by computers.* Retrieved from www.fon.hum.uva.nl/praat/, v. 4.3.04.

Clopper, C. G., & Pisoni, D. B. (2004). Effects of talker variability on perceptual learning dialects. *Language and Speech, 47,* 207–239.

Dean, I., Harper, N. S., & McAlpine, D. (2005). Neural population coding of sound level adapts to stimulus statistics. *Nature Neuroscience, 8,* 1684–1689.

Delano, P. H., Elgueda, D., Hamame, C. M., & Robles, L. (2007). Selective attention to visual stimuli reduces cochlear sensitivity in chinchillas. *Journal of Neuroscience, 27,* 4146–4153.

Escabi, M. A., Miller, L. M., Read, H. L., & Schreiner, C. E. (2003). Naturalistic auditory contrast improves spectrotemporal coding in the cat inferior colliculus. *Journal of Neuroscience, 23,* 11489–11504.

Fahle, M., & Poggio, T. (2002). *Perceptual learning*. MIT Press.

Feldman, A., & Healy, A. F. (1998). *Foreign language learning: Psychological studies on training & retention*. Mahwah, NJ: Erlbaum.

Fujioka, T., Ross, B., Kakigi, R., Pantev, C., & Trainor, L. J. (2006). One year of musical training affects development of auditory cortical-evoked fields in young children. *Brain, 129,* 2593–2608.

Galbraith, G. C., Threadgill, M. R., Hemsley, J., Salour, K., Songdej, N., Ton, J., et al. (2000). Putative measure of peripheral and brainstem frequency-following in humans. *Neuroscience Letters, 292,* 123–127.

Gordon, M., & O'Neill, W. E. (2000). An extralemniscal component of the mustached bat inferior colliculus selective for direction and rate of linear frequency modulations. *Journal of Comparative Neurology, 426,* 165–181.

Gorga, M., Abbas, P., & Worthington, D. (1985). Stimulus calibration in ABR measurements. In J. Jacobson (Ed.), *The auditory brainstem response*. San Diego: College-Hill.

Gottfried, T. L., & Suiter, T. L. (1997). Effect of linguistic experience on the identification of Mandarin Chinese vowels and tones. *Journal of Phonetics, 25,* 207–231.

Hall, J. W., III (1979). Auditory brainstem frequency following responses to waveform envelope periodicity. *Science, 205,* 1297–1299.

Hood, L. (1998). *Clinical applications of the auditory brainstem response*. San Diego: Singular Publishing Group.

Hoormann, J., Falkenstein, M., Hohnsbein, J., & Blanke, L. (1992). The human frequency-following response (FFR): Normal variability and relation to the click-evoked brainstem response. *Hearing Research, 59,* 179–188.

Johnson, K. L., Nicol, T., & Kraus, N. (2005). The brainstem response to speech: A biological marker of auditory processing. *Ear and Hearing, 26,* 424–434.

Kiriloff, C. (1969). On the auditory perception of tones in Mandarin. *Phonetica, 20,* 63–67.

Knudsen, E. (2004). Sensitive periods in the development of the brain and behavior. *Journal of Cognitive Neuroscience, 16,* 1412–1425.

Kraus, N., McGee, T., Carrell, T. D., King, C., Tremblay, K., & Nicol, T. (1995). Central auditory system plasticity associated with speech discrimination training. *Journal of Cognitive Neuroscience, 7,* 25–32.

Kraus, N., & Nicol, T. (2005). Brainstem origins for cortical ''what'' and ''where'' pathways in the auditory system. *Trends in Neurosciences, 28,* 176–181.

Krishnan, A., Xu, Y., Gandour, J., & Cariani, P. (2005). Encoding of pitch in the human brainstem is sensitive to language experience. *Cognitive Brain Research, 25,* 161–168.

Langner, G. (1997). Neural processing and representation of periodicity pitch. *Acta Oto-laryngologica Supplementum, 532,* 68–76.

Liebenthal, E., Binder, J. R., Spitzer, S. M., Possing, E. T., & Medler, D. A. (2005). Neural substrates of phonemic perception. *Cerebral Cortex, 15,* 1621–1631.

Lively, S. E., Logan, J. S., & Pisoni, D. B. (1993). Training Japanese listeners to identify English /r/ and /l/: II. The role of phonetic environment and talker variability in learning new perceptual categories. *Journal of the Acoustical Society of America, 93,* 1242–1255.

Uncorrected Proof

Marsh, J. T., & Worden, F. G. (1968). Sound evoked frequency-following responses in the central auditory pathway. *Laryngoscope, 78,* 1149–1163.

Maye, J., Werker, J. F., & Gerken, L. (2002). Infant sensitivity to distributional information can affect phonetic discrimination. *Cognition, 82,* B101–B111.

Moller, A. R. (1999). Review of the roles of temporal and place coding of frequency in speech discrimination. *Acta Otolaryngologica, 119,* 424–430.

Musacchia, G., Sams, M., Skoe, E., & Kraus, N. (2007). Musicians have enhanced subcortical auditory and audiovisual processing of speech and music. *Proceedings of the National Academy of Sciences, U.S.A., 104,* 15894–15898.

Näätänen, R., Lehtokoski, A., Lennes, M., Cheour, M., Huotilainen, M., Iivonen, A., et al. (1997). Language-specific phoneme representations revealed by electric and magnetic brain responses. *Nature, 385,* 432–434.

Nosofsky, R. M. (1986). Attention, similarity, and the identification–categorization relationship. *Journal of Experimental Psychology: General, 115,* 39–61.

Pierrehumbert, J. (1979). The perception of fundamental frequency declination. *Journal of the Acoustical Society of America, 66,* 363–369.

Recanzone, G. H., Jenkins, W. M., Hradek, G. T., & Merzenich, M. M. (1992). Progressive improvement in discriminative abilities in adult owl monkeys performing a tactile frequency discrimination task. *Journal of Neurophysiology, 67,* 1015–1030.

Russo, N. M., Nicol, T. G., Zecker, S. G., Hayes, E. A., & Kraus, N. (2005). Auditory training improves neural timing in the human brainstem. *Behavioural Brain Research, 156,* 95–103.

Schoups, A., Vogels, R., Qian, N., & Orban, G. (2001). Practising orientation identification improves orientation coding in V1 neurons. *Nature, 412,* 549–553.

Seitz, A. R., & Watanabe, T. (2003). Psychophysics: Is subliminal learning really passive. *Nature, 422,* 36.

Shahin, A., Bosnyak, D. J., Trainor, L. J., & Roberts, L. E. (2003). Enhancement of neuroplastic P2 and N1c auditory evoked potentials in musicians. *Journal of Neuroscience, 23,* 5545–5552.

Shahin, A. J., Roberts, L. E., Pantev, C., Aziz, M., & Picton, T. W. (2007). Enhanced anterior-temporal processing for complex tones in musicians. *Clinical Neurophysiology, 118,* 209–220.

Smith, J. C., Marsh, J. T., & Brown, W. S. (1975). Far-field recorded frequency-following responses: Evidence for the locus of brainstem sources. *Electroencephalography and Clinical Neurophysiology, 39,* 465–472.

Suga, N., Gao, E., Zhang, Y., Ma, X., & Olsen, J. F. (2000). The corticofugal system for hearing: Recent progress. *Proceedings of the National Academy of Sciences, U.S.A., 97,* 11807–11814.

Tervaniemi, M., Jacobsen, T., Rottger, S., Kujala, T., Widmann, A., Vainio, M., et al. (2006). Selective tuning of cortical sound-feature processing by language experience. *European Journal of Neuroscience, 23,* 2538–2541.

Tremblay, K., Kraus, N., Carrell, T. D., & McGee, T. (1997). Central auditory system plasticity: Generalization to novel stimuli following listening training. *Journal of the Acoustical Society of America, 102,* 3762–3773.

Tremblay, K., Kraus, N., McGee, T., Ponton, C., & Otis, B. (2001). Central auditory plasticity: Changes in the N1–P2 complex after speech–sound training. *Ear and Hearing, 22,* 79–90.

Trice, R., & Carrell, T. (1998). *Level 16*.

Watanabe, T., Nanez, J. E., & Sasaki, Y. (2001). Perceptual learning without perception. *Nature, 413,* 844–848.

Wechsler, D. (1999). *Wechsler Abbreviated Scale of Intelligence*. San Antonio, TX: The Psychological Corporation.

Wong, P. C. M., & Perrachione, T. K. (2007). Learning pitch patterns in lexical identification by native English-speaking adults. *Applied Psycholinguistics, 28,* 565–585.

Wong, P. C. M., Perrachione, T. K., & Parrish, T. B. (2007). Neural characteristics of successful and less successful speech and word learning in adults. *Human Brain Mapping, 28,* 995–1006.

Wong, P. C. M., Skoe, E., Russo, N. M., Dees, T., & Kraus, N. (2007). Musical experience shapes human brainstem encoding of linguistic pitch patterns. *Nature Neuroscience, 10,* 420–422.

Zatorre, R. J., Evans, A. C., Meyer, E., & Gjedde, A. (1992). Lateralization of phonetic and pitch discrimination in speech processing. *Science, 256,* 846–849.

Uncorrected Proof
