# Supplementary material for: Neural Processing of Speech Sounds in ASD and First-Degree Relatives
Source: J Autism Dev Disord. 2022 Jun 7;53(8):3257–71. doi: 10.1007/s10803-022-05562-7 (PMC10019095; doi:10.1007/s10803-022-05562-7)
Supplement: Supplementary file 60 — Supplementary file60 (PDF 615 kb) [file 10803_2022_5562_MOESM60_ESM.pdf]

Figure S1. f_0 contours of the three stimuli (f_0 ranges: 140-172 Hz, 110-163 Hz, and 89-110 Hz, respectively).

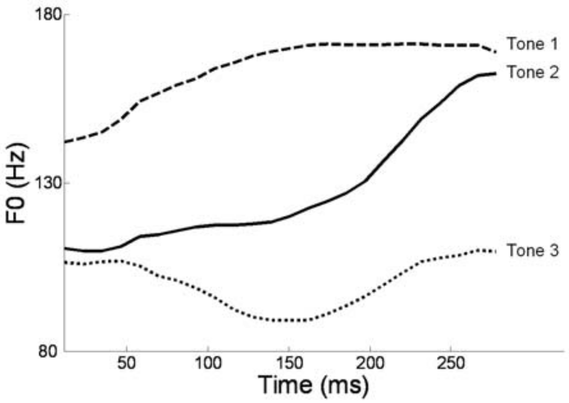

Table S1. Subjects' musical history. Second and third columns indicate years of musical training and age at which musical training began (age onset), respectively. Mean age onset for nonmusicians was based on six subjects only.

**Musician**	**Years of Training**	**Age Onset (Years)**	**Instrument (Lesson Type)**
#1	6	6	Clarinet (Group)
#2	11	11	Piano (Private)
#3	12	12	Piano (Private)
#4	7	7	Piano (Private)
#5	8	11	Trumpet (Private)
#6	12	7	Piano (Private)
#7	12	7	Piano (Private)
#8	8	10	Piano (Private)
#9	12	7	Piano (Private)
#10	19	5	Piano (Private)
**Mean**	**10.7**	**8.3**	
**Nonmusician**			
#11	2	7	Keyboard (Private)
#12	2	10	Clarinet (Group)
#13	0	N/A	N/A
#14	1	15	Trombone (Private)
#15	3	15	Piano (Private)
#16	0	N/A	N/A
#17	1	10	Violin (Group)
#18	0	N/A	N/A
#19	0	N/A	N/A
#20	3	14	Piano (Private)
**Mean**	**1.2**	**13.33**	

SUPPLEMENTARY METHODS

Subjects

Twenty subjects (11 females) participated in this study. None of the subjects had previous exposure to a tone language. Subjects were divided into two groups based on musical training. Amateur musicians were defined as instrumentalists having at least six years of continuous musical training (mean = 10.7 years) starting at or before the age of 12, in addition to currently playing their instrument. Nonmusicians were defined as having no more than three years of musical training (mean = 1.2 years) at any time in their life. Subjects' musical history information is summarized in **Table S1**. All subjects were right handed and reported no audiologic or neurologic deficits. All subjects had normal click-evoked auditory brainstem response latencies and normal hearing thresholds at or below 20 dB HL for octaves from 125 to 4000 Hz. The two subject groups did not differ in age or handedness scores.

Stimuli

A native speaker of Mandarin Chinese was asked to produce /mi/ with three Mandarin tones: /mi1/ 'to squint,' /mi2/ 'bewilder,' and /mi3/ 'rice' (by convention, the number indicates tone or lexically meaningful pitch contour: Tone 1 = level tone, Tone 2 = rising tone, and Tone 3 = dipping tone). Recording took place in a sound attenuated chamber using a SHURE SM58 microphone recorded at 44.1 kHz onto a Pentium IV PC. These original productions were then duration-normalized to 278.5 milliseconds (ms) using Praat[1]. Using Praat, the pitch (f_0) contours of each of the original production were extracted and then superimposed onto the original Tone 1 (/mi1/) production using the

Pitch-Synchronous Overlap and Add (PSOLA) method, which resulted in perceptually natural stimuli as judged by four native speakers of Mandarin. The stimuli, therefore, consisted of three instances of /mi/ (in three Mandarin tones) differing only in f_0. These stimuli were RMS amplitude normalized using the software Level 16[2]. To accommodate the capabilities of our stimulus presentation software, the stimuli were resampled to 22.05 kHz. **Fig. S1** shows the f_0 contours of the three stimuli (f_0 ranges: 140-172 Hz, 110-163 Hz, and 89-110 Hz, respectively). It is worth pointing out that we use the term "linguistic pitch" to describe these f_0 contours because they were embedded in speech, not music. We realize that none of our subjects spoke a tone language and thus these f_0 contours were not lexicalized. It is, therefore, likely that these f_0 contours were interpreted as intonational tones, which also carry linguistic functions[3].

Physiologic (ERP) Recording Procedures

Physiologic recording procedures were similar to our published studies (e.g., Russo et al.[4]). During testing, subjects watched a videotape with the sound level set at < 40 dB SPL to facilitate a quiet yet wakeful state. Subjects listened to the video soundtrack (presented in free field) with the left ear unoccluded, while the stimuli were presented to the right ear through ER-3 ear inserts (Etymotic Research, Elk Grove Village, IL) at about 70 dB SPL (Stim, AUDCPT, Compumedics, El Paso, TX). The order of the three stimuli was randomized across subjects with a variable inter-stimulus interval between 71.50 and 104.84 ms. Responses were collected using Scan 4.3 (Compumedics, El Paso, TX) with Ag–AgCl scalp electrodes, differentially recorded from Cz (active) to ipsilateral earlobe (reference), with the forehead as ground. Two blocks of 1200 sweeps per block

were collected at each polarity with a sampling rate of 20 kHz. Filtering, artifact rejection and averaging were performed offline using Scan 4.3. Responses were bandpass filtered from 80-1000 Hz, 12 dB/octave, and trials with artifacts greater than 35 μV were rejected. Waveforms were averaged with a time window spanning 45 ms prior to the onset and 16.5 ms after the offset of the stimulus. Responses of alternating polarity were then added together to isolate the neural response by minimizing stimulus artifact and cochlear microphonic[5]. For the purpose of calculating signal-to-noise ratios, a single waveform representing non-stimulus-evoked neural activity was created by averaging the neural activity 45 ms prior to stimulus onset.

Analysis Procedures

For each subject, we calculated two primary measures of FFR pitch-tracking: *stimulus-to-response correlation* and *autocorrelation*. These measures were derived using a sliding-window analysis procedure in which 40-ms bins of the FFR were analyzed in the frequency and lag (autocorrelation) domains. The FFR was assumed to encompass the entire response beginning at time 1.1 ms, the transmission delay between the ER-3 transducer and ear insert. The 40-ms sliding window was shifted in 1 ms steps, to produce a total of 238 overlapping bins. A narrow-band spectrogram was calculated for each FFR bin by applying the Fast Fourier Transform (FFT) to windowed bins (Hanning window) of the signal. To increase spectral resolution, each time bin was zero-padded to 1 second before performing the FFT. The spectrogram gave an estimate of spectral energy over time and the f_0 (pitch) contour was extracted from the spectrogram by finding the frequency with the largest spectral magnitude for each time bin. Spectral peaks that

did not fall above the noise-floor were excluded as possible f_0 candidates. Both f_0 frequency and magnitude were recorded for each time bin, and the f_0 *amplitude* measure was calculated as the average magnitude across bins. The same short-term spectral analysis procedure was applied to the stimulus waveforms to calculate the degree of similarity (*stimulus-to-response correlation*) between the stimulus and response f_0 contours, defined as the Pearson's correlation coefficient (r) between the stimulus and response f_0 contours. This measure represents both the strength and direction of the linear relationship between to two signals.

The second measure of pitch-tracking, *autocorrelation*, was derived using a pitch-detection short-term autocorrelation method[6]. Each of the 238 time bins was cross-correlated with itself to determine how well the bin matched a time-shifted version of itself. The maximum (peak) autocorrelation value (expressed as a value between 0 and 1) was recorded for each bin, with higher values indicating more periodic time frames. The *autocorrelation* pitch tracking measure was calculated by averaging the autocorrelation peaks (r-values) from the 238 bins for each tone for each subject. Running-autocorrelograms (lag versus time) (see Krishnan et al.[7]) were calculated as a means of visualizing and quantifying periodicity and pitch strength variation over the course of the response. In the pitch-tracking and autocorrelation plots (**Fig. 1**, middle and bottom panels), the time indicated on the x-axis refers to the midpoint of each 40-ms time bin analyzed. For example, the f_0 extracted from the first FFR time bin (1.1 ms - 40.1 ms) is plotted at time 21.1 ms.

We also measured the RMS (Root-Mean-Square) amplitude of the FFR waveform, which is the magnitude of neural activation over the entire FFR period (1.1 –

295 ms). This measure takes both negative and positive peaks into consideration. This *FFR RMS amplitude* is driven largely by the amplitude of the f_0 (a description of the f_0 *amplitude* calculation is provided above*)*. If a subject has robust pitch-tracking, the largest peaks in the response waveform will fall at the period of the f_0. In addition, to quantitatively consider the proportion of the f_0 amplitude relative to the overall *FFR RMS amplitude*, we calculated f_0-*FFR proportion*, which is the average f_0 amplitude divided by the total RMS amplitude.

The use of multiple pitch-tracking measures allows us to more comprehensively observe and quantify pitch encoding differences between the two groups. All pitch-tracking analyses were performed using routines coded in Matlab 7.4.1 (Mathworks, Natick, MA , 2005).

Behavioral Testing (Tone Identification and Discrimination)

Subjects also participated in two behavioral experiments designed to test their ability to identify and discriminate Mandarin tones. The stimuli and procedures, summarized briefly here, were essentially identical to Alexander, Wong, and Bradlow[8]. Stimuli consisted of twenty monosyllabic Mandarin Chinese words. The five syllables /bu/, /di/, /lu/, /ma/, /mi/ were each produced in citation form with the four tones (level, rising, dipping, and falling) of Mandarin. Talkers consisted of two male and two female native speakers of Mandarin Chinese. Subjects participated in these two experiments after task familiarization. In tone identification, subjects matched the auditory stimulus with visually presented arrows depicting the pitch trajectory. In tone discrimination, subjects made a same-different judgment on the pitch patterns of stimulus pairs.

REFERENCES

1. Boersma, P. & Weenink, D. (2004)
2. Tice, R. & Carrell, T. D. (1998).
3. Pierrehumbert, J. B. (MIT, Cambridge, 1980)
4. Russo, N., Musacchia, G., Nicol, T., Zecker, S. & Kraus, N. Brainstem responses to speech syllables. *Clinical Neurophysiology* **115**, 2021-2030 (2004).
5. Gorga, M. P., Abbas, P. J. & Worthington, D. W. in *The Auditory Brainstem Response* (ed. Jacobson, J. T.) 49-62 (College-Hill Press, San Diego, 1985).
6. Boersma, P. in *Proceedings of Institute of Phonetic Sciences* 97-110 (Amsterdam, 1993).
7. Krishnan, A., Xu, Y., Gandour, J. & Cariani, P. Encoding of pitch in the human brainstem is sensitive to language experience. *Cogn Brain Res* **25**, 161-8 (2005).
8. Alexander, J., Wong, P. C. M. & Bradlow, A. in *Proceedings of Interspeech 2005 - Eurospeech - 9th European Conference on Speech Communication and Technology* (2005).

SUPPLEMENTARY RESULTS

Behavioral Measures

Independent samples *t*-tests showed musicians to have significantly higher tone identification [t (18) = 3.664, $P < 0.005$] and tone discrimination [t (18) = 3.224, $P < 0.005$] scores than nonmusicians. The mean (and standard error) tone identification scores for musicians and nonmusicians are 0.9090 (0.0223) and 0.7450 (0.0388), respectively. The mean (and standard error) tone discrimination scores for musicians and nonmusicians are 0.9000 (0.0132) and 0.7480 (0.0450), respectively.

Pitch-Tracking Measures

Because Pearson's correlation coefficients do not comprise a normal distribution, *stimulus-to-response correlation* and *autocorrelation* measures were converted to Fisher's z' scores before subsequent parametric statistical analyses.

In terms of *stimulus-to-response correlation*, a 3 (tone) x 2 (group) repeated measures ANOVA revealed main effects of tone [F (2, 36) = 17.985, $P < 0.001$] and group [F (1, 18) = 8.039, $P < 0.015$] but no significant interaction. Thus, musicians showed more precise pitch-tracking compared to nonmusicians. To examine in detail any possible tone differences between groups, we performed one independent samples *post hoc t*-tests and found no pitch-tracking difference in Tone 1 but marginally significant group differences in Tone 2 [t (18) = 2.237, $P = 0.019$], and significant group difference in Tone 3 [t (18) = 2.355, $P = 0.015$] (per Bonferroni procedures, $P < 0.016$ is required for establishing statistical significance for the three tests performed: 0.05/3 = 0.01667),

suggesting that the main effect of group was largely driven by Tone 3 and less so by Tone 1 and Tone 2.

Likewise, *autocorrelation* values were entered into a 3 x 2 repeated measures ANOVA, which showed a main effect of tone [F (2, 36) = 14.135, $P < 0.001$], but no main effect of group [F (1, 18) = 1.819, $P = 0.194$]). A marginally significant group x tone interaction was found [F (2, 36) = 2.741, $P = 0.078$]. Based on the visual inspection of the autocorrelation plots showing musicians to have more robust responses on Tone 3 but not the other tones, a one independent samples t-test was performed on Tone 3, which confirmed musicians' higher *autocorrelation* values [t (18) = 2.059, $P = 0.027$]. Overall, these pitch-tracking measures showed musicians to have more robust neural phase-locking and more faithful pitch-tracking. This group difference was particularly pronounced in Tone 3, the most acoustically complex tone.

In addition to the above pitch-tracking measures, we also compared musicians' and nonmusicians' f_0 *amplitude* for each tone to examine whether the pitch-tracking differences discussed above could also be attributed to musicians' stronger FFR. A repeated measures ANOVA revealed a main effect of group (stronger in musicians) [F (1, 18) = 4.385, $P = 0.051$], a main effect of tone [F (2, 36) = 3.315, $P < 0.05$] but no significant interaction. *RMS amplitude* of the FFR waveform was likewise compared. We found a main effect of group (stronger in musicians) [F (1, 18) = 4.329, $P = 0.052$], a main effect of tone [F (2, 36) = 6.693, $P < 0.005$], but no significant interaction. Moreover, to examine whether f_0-*FFR proportion* differed between the two subject groups, a series of Mann-Whitney U-tests (on each tone) were performed and no

significant group difference was found. Non-parametric tests were used because of the non-normal distribution of the data derived from this measure.

Correlations between Pitch-Tracking Measures and Music Background

To investigate the relationship between pitch-tracking measures and musical experience, a series of Pearson's correlations were calculated. We calculated correlations between each pitch-tracking measure and years of musical training, as well as age at which musical training began (age onset). Four of the subjects had no formal musical training, and thus were not included in the correlation analyses involving age onset. Given that Tone 3 is the only tone shown to differ consistently between musicians and nonmusicians, only measures derived from this tone were considered. The correlations between years of musical training and *stimulus-to-response correlation* for Tone 3 and *autocorrelation* for Tone 3 are 0.456 ($P = 0.022$) and 0.549 ($P = 0.006$), respectively ($P < 0.025$ is required to establish statistical significance for the two tests performed). The correlations between age onset and stimulus-to-response correlation for Tone 3 and autocorrelation for Tone 3 are –0.502 ($P = 0.024$) and –0.332 ($P = 0.105$), respectively.

SUPPLEMENTARY DISCUSSION

As discussed above, measures of FFR pitch-tracking were calculated and compared between the two subject groups, musicians and nonmusicians. FFR is presumed to originate from the inferior colliculus (based on data from human[1] and animal lesion[2]); however, it is also possible that even lower brainstem nuclei contribute to this response[3]. These brainstem nuclei (inferior colliculus and subcollicular auditory nuclei) receive efferent inputs from deep layers of the auditory cortex (see Suga et al.[4] for a review). Therefore, the top-down mechanism we advocate could be made feasible via this anatomical pathway from cortex to the brainstem (collicular and subcollicular nuclei).

It is worth emphasizing that the subjects in the current study were not asked to perform a pitch perception task during physiologic recording, nor were they asked to selectively attend to the auditory stimuli. In fact, subjects were asked to watch the video while ignoring the background auditory stimuli. With the congruent presentation of visual images and soundtrack, we have strong reasons to believe that our subjects were indeed attending to the more engaging audiovisual video rather than the monotonous and irrelevant background stimuli (which were essentially noise to the subjects). Our procedures to elicit "pre-attentive" electrophysiologic responses have been used repeatedly by our group[5-8] and others[9] and in different subject populations[10, 11] (e.g., subjects with peripheral hearing impairment, learning impaired children). One interpretation of our results is that musicians were better able to use their online top-down mechanism to either ignore the video soundtrack or to attend to the Mandarin stimuli, actively or not. This interpretation would suggest a more general top-down attentional mechanism rather than a mechanism that is auditory or pitch specific. Although we

found this interpretation to be plausible, we would like to point out that attentional effects influencing brainstem potentials have been found to be highly task-specific, and in all known cases, required subjects to explicitly perform a task (e.g., when subjects were asked to selectively attend to either visual or auditory stimuli, or when a target tone is compared to a proceeding reference tone[12,13]. Also of note, Hoormann et al.[13] did not find attentional effects on brainstem potentials in a dichotic listening paradigm. Importantly, this attention-driven interpretation and the interpretation we advocate both involve corticofugal modulations of brainstem circuitry.

It is reasonable to expect online corticofugal influence to play a role in the present results. That is, over the course of the recording, stimulus encoding is fined-tuned by the repeated exposure to the same set of stimuli, and this within-session tuning is enhanced by corticofugal mechanisms. However, it is important to point out that we believe a critical portion of this corticofugal effect is driven by long-term experience with music, resulting in the intrinsic properties of the inferior colliculus being enhanced to better able encode pitch, in general. Although the corticofugal mechanisms we advocate here are still speculative at this stage, and unsupervised bottom-up tuning mechanisms are not being completely ruled out, we believe top-down mechanisms are the most plausible in that they account for most of the results, especially given converging data from Krishnan's group. Our argument is consistent with the argument put forth by Krishnan et al.[9], who attributed their results to native Mandarin speakers' long-term exposure to Mandarin pitch. The importance of the present study lies in the suggestion that our results demonstrate context-general effects on the brainstem responses to speech. However, note that although musicians possess better pitch encoding of Mandarin tones,

their encoding is not as robust as the native-Mandarin participants' analyzed by Krishnan and colleagues[9] using comparable techniques. This suggests that contextualized exposure (e.g., speech exposure effects on speech performance) in contrast to non-contextualized exposure, still shapes the best response.

REFERENCES

1. Greenberg, S., Marsh, J. T., Brown, W. S. & Smith, J. C. Neural temporal coding of low pitch. I. Human frequency-following responses to complex tones. *Hear Res* **25**, 91-114 (1987).
2. Davis, R. L. & Britt, R. H. Analysis of the frequency following response in the cat. *Hear Res* **15**, 29-37 (1984).
3. Hoormann, J., Falkenstein, M., Hohnsbein, J. & Blanke, L. The human frequency-following response (FFR): normal variability and relation to the click-evoked brainstem response. *Hear Res* **59**, 179-88 (1992).
4. Suga, N., Gao, E., Zhang, Y., Ma, X. & Olsen, J. F. The corticofugal system for hearing: recent progress. *Proc Natl Acad Sci U S A* **97**, 11807-14 (2000).
5. Banai, K., Nicol, T., Zecker, S. G. & Kraus, N. Brainstem timing: implications for cortical processing and literacy. *J Neurosci* **25**, 9850-7 (2005).
6. Abrams, D. A., Nicol, T., Zecker, S. G. & Kraus, N. Auditory brainstem timing predicts cerebral asymmetry for speech. *J Neurosci* **26**, 11131-7 (2006).
7. Johnson, K., Nicol, T. & Kraus, N. The Brainstem Response to Speech: A Biological Marker of Auditory Processing. *Ear Hear* **26**, 424-34 (2005).
8. Kraus, N. & Nicol, T. Brainstem origins for cortical 'what' and 'where' pathways in the auditory system. *Trends Neurosci* **28**, 176-81 (2005).
9. Krishnan, A., Xu, Y., Gandour, J. & Cariani, P. Encoding of pitch in the human brainstem is sensitive to language experience. *Cogn Brain Res* **25**, 161-8 (2005).
10. Hood, L. J. *Clinical Applications of the Auditory Brainstem Response* (Singular Publishing Group, San Diego, 1998).
11. Jacobson, J. T. *The Auditory Brainstem Response* (College-Hill Press, San Diego, 1985).
12. Galbraith, G. C., Olfman, D. M. & Huffman, T. M. Selective attention affects human brain stem frequency-following response. *Neuroreport* **14**, 735-8 (2003).
13. Hoormann, J., Falkenstein, M. & Hohnsbein, J. Early attention effects in human auditory-evoked potentials. *Psychophysiology* **37**, 29-42 (2000).
