# Supplementary material for: Neural Processing of Speech Sounds in ASD and First-Degree Relatives
Source: J Autism Dev Disord. 2022 Jun 7;53(8):3257–71. doi: 10.1007/s10803-022-05562-7 (PMC10019095; doi:10.1007/s10803-022-05562-7)
Supplement: Supplementary file 61 — Supplementary file61 (PDF 163 kb) [file 10803_2022_5562_MOESM61_ESM.pdf]

nature neuroscience

© 2007 Nature Publishing Group http://www.nature.com/natureneuroscience

Musical experience shapes human brainstem encoding of linguistic pitch patterns

Patrick C M Wong[1,2], Erika Skoe[1,5], Nicole M Russo[1,2,5], Tasha Dees[1] & Nina Kraus[1–4]

Music and speech are very cognitively demanding auditory phenomena generally attributed to cortical rather than subcortical circuitry. We examined brainstem encoding of linguistic pitch and found that musicians show more robust and faithful encoding compared with nonmusicians. These results not only implicate a common subcortical manifestation for two presumed cortical functions, but also a possible reciprocity of corticofugal speech and music tuning, providing neurophysiological explanations for musicians' higher language-learning ability.

Both music and spoken language involve the use of functionally and acoustically complex sound and are generally attributed to the neocortex[1–4]. Less is known about how long-term experience using these complex sounds shapes subcortical circuitry and the context specificity and reciprocity of this tuning[5]. By measuring the frequency following response (FFR), which presumably originates from the auditory brainstem (inferior colliculus) and encodes the energy of the stimulus fundamental frequency (f_0) with high fidelity[6], previous work[7] has found increased linguistic pitch pattern encoding in Mandarin-speaking subjects relative to English-speaking subjects. These results reflect Mandarin-speaking subjects' long-term exposure to linguistic pitch patterns, as Mandarin Chinese, a tone language, uses pitch to signal word meaning (for example, /ma/ spoken with high or rising pitch patterns means 'mother' or 'numb', respectively). Moreover, similar to research on short-term perceptual learning[8], these results can be viewed as context specific (that is, linguistic experiences, subserved by the cortex, enhance the encoding of linguistic information at the brainstem). The nonspecificity of this long-term usage effect, though largely unknown, is both theoretically interesting and clinically and educationally relevant. Nonspecificity would suggest that either speech- or music-related experience can tune sensory encoding in the auditory brainstem via the corticofugal pathway. Notably, this tuning, whether speech- or music-induced, would enhance all relevant auditory functions (both speech and music) subserved by the rostral brainstem.

We measured FFR responses to linguistic pitch patterns at the rostral brainstem in ten amateur musicians and ten nonmusicians who had no previous exposure to a tone language (see **Supplementary Table 1** online). Musicians (instrumentalists) had at least 6 years of continuous musical training (mean = 10.7 years) starting at or before the age of 12. Nonmusicians had no more than 3 years (mean = 1.2 years) at any time in their life. Informed written consent was obtained from all subjects. While watching a video, subjects listened to three randomly presented Mandarin stimuli resynthesized to differ only in f_0: /mi1/ 'to squint', /mi2/ 'bewilder' and /mi3/ 'rice' (by convention, the number indicates tone or lexically meaningful pitch contour: Tone 1 = level tone, Tone 2 = rising tone and Tone 3 = dipping tone; see **Supplementary Methods** and **Supplementary Fig. 1** online for details). Brainstem

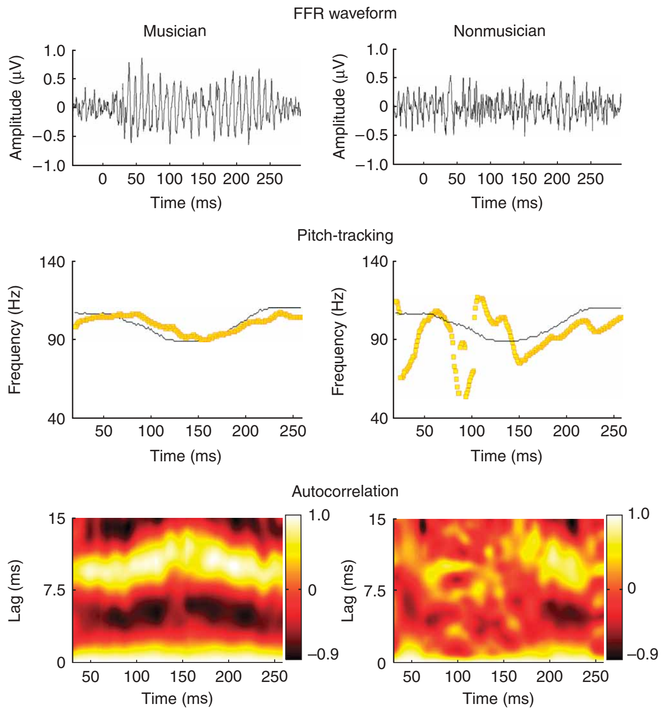

Figure 1 Frequency following responses from selected subjects. Top, FFR waveforms from a musician (left) and nonmusician (right) elicited by a dipping pitch contour (Tone 3). Middle, trajectories (yellow line) of brainstem pitch tracking elicited by the same tone from the same subjects. The black line indicates the stimulus (expected) f_0 contour. Bottom, autocorrelograms of the FFR waveforms. Color indicates the degree of correlation, with lighter colors indicating higher correlations. For the musician (left panel), the light band of color closely follows the inverse of the pitch contour of Tone 3 (frequency = 1/lag). In contrast, the nonmusician's autocorrelogram (right panel) is more diffuse and the highly correlated regions are not localized to the period of the f_0 of the stimulus.

[1]The Roxelyn and Richard Pepper Department of Communication Sciences and Disorders. [2]Northwestern University Interdepartmental Neuroscience Program, [3]Department of Neurobiology and Physiology, [4]Department of Otolaryngology, Northwestern University, 2240 Campus Drive, Evanston, Illinois 60208-3540, USA. [5]These authors contributed equally to this work. Correspondence should be addressed to P.C.M.W. (pwong@northwestern.edu).

Received 8 December 2006; accepted 16 February 2007; published online 11 March 2007; doi:10.1038/nn1872

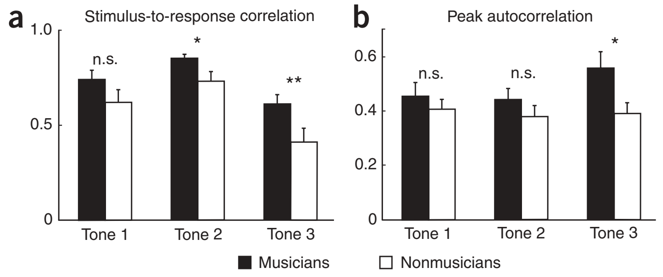

Figure 2 Pitch tracking group results. (**a**,**b**) Mean stimulus-to-response correlation (**a**) and autocorrelation (**b**); black and white bars show averaged results from musicians and nonmusicians, respectively (error bars indicate one standard error; $*P < 0.03$ and $**P < 0.016$ based on independent samples *t*-tests).

responses were collected using Scan 4.3 (Compumedics) with Ag–AgCl scalp electrodes. After f_0 extraction (**Supplementary Methods**), we derived two primary measures of pitch tracking for each subject for each tone. First is the stimulus-to-response correlation (Pearson's *r* between the f_0 contour of the stimulus and the subject's response contour), which indicates faithfulness of pitch tracking. Second is peak autocorrelation averaged over the entire response, which indicates robustness of neural phase-locking without making reference to the stimulus. In addition to these two primary pitch-tracking measures, we also considered the f_0 amplitude of the FFR (which represents the average amount of spectral energy devoted to encoding the changing f_0), the root-mean-square (RMS) amplitude of the FFR waveform, correlations between musical experience and pitch tracking, and subjects' tone perception (behavioral) performances (see **Supplementary Methods**).

Each of the primary measures was entered into a 3 (tone) × 2 (group) repeated measures ANOVA (for stimulus-to-response correlation, there was a main effect of group, $P < 0.015$, and tone, $P < 0.001$, but no significant interaction; for autocorrelation, there was a significant effect of tone, $P < 0.001$, but not of group, and a marginally significant interaction, $P < 0.08$) followed by independent samples *t*-tests comparing group differences for each tone. The significance level was corrected for multiple comparisons following Bonferroni procedures. Overall, musicians showed more faithful representation of the stimulus f_0 contours (**Fig. 1**, middle panels; **Fig. 2a**) and more robust neural phase-locking (**Fig. 1**, bottom panels; **Fig. 2b**; see **Supplementary Results** online for details), particularly for the most complex contour (Tone 3). Musicians also showed stronger overall f_0 amplitude and FFR RMS amplitude than nonmusicians (**Fig. 1**, top panels). Moreover, there was a significant positive correlation between the pitch tracking of the most complex contour and music experience (**Fig. 3**). Subjects also participated in tone identification and discrimination tasks, in which musicians showed significantly better identification ($t(18) = 3.664$, $P < 0.005$) and discrimination ($t(18) = 3.224$, $P < 0.005$). Subjects' performance on the discrimination task was significantly correlated with Tone 3 tracking (Pearson's $r = 0.434$, $P = 0.028$).

Musical ability predicts the ability to produce and perceive the sound structures, but not grammatical or semantic structures, of a second language[9]. More specifically, musicians have an enhanced ability to learn lexical tones[4]. Here, we found a plausible neurophysiological (subcortical) correlate of the effect of long-term musical training on speech (prosodic) encoding. Musicians have extensive experience using pitch information in the context of music, which requires both high cognitive demands and auditory acuity. This functional interplay is possibly mediated via feedback from the higher-level cortex to the inferior colliculus (made possible anatomically by the corticofugal pathway[10]), such that accurate pitch information is relayed from subcortical structures to the neocortex to facilitate successful performance of cognitively demanding tasks. Cortical electrophysiology shows musical training to facilitate language processing in adults[11], and we are the first to show this effect in brainstem responses. Our line of reasoning is consistent with models of supervised perceptual learning involving changes in the weighting of perceptual dimensions as a result of feedback[12] and is also consistent with the reverse hierarchy theory of visual learning, which suggests that learning consists of an attention-driven, task-dependent 'backward' search for increased signal-to-noise ratio, especially for perceptual experts[13]. An important aspect of our results is that the musicians showed more robust and faithful neural encoding elicited by nonmusic stimuli, suggesting that corticofugal modulation is not entirely context specific. However, whether context-specific exposure still shapes the best response (for example, speech exposure effects on speech performance) requires further experimentation.

Although the current study provides evidence for the positive effect of long-term music exposure on speech (linguistic pitch) encoding at the brainstem, especially given the significant correlation between brainstem pitch tracking and music experience (in terms of both age of onset and years of musical training), we acknowledge that genetic differences between our musician and nonmusician groups could potentially account for the results. Moreover, our conclusion is limited by the small set of stimuli (Mandarin tones) used. However, because we have now established a robust effect and observed the pervasive impact of musical training on our nervous system, we believe a new line of research has been opened up, which would naturally involve more comprehensive and systematic investigations of musicians' and nonmusicians' responses to different simple and complex sounds.

In sum, we found more robust and faithful encoding of linguistic pitch information by musicians. Such encoding, arguably associated with increased musical pitch usage, may reflect a positive side effect of context-general corticofugal tuning of the afferent system, implying that long-term music-making may shape basic sensory circuitry. These results complement our existing

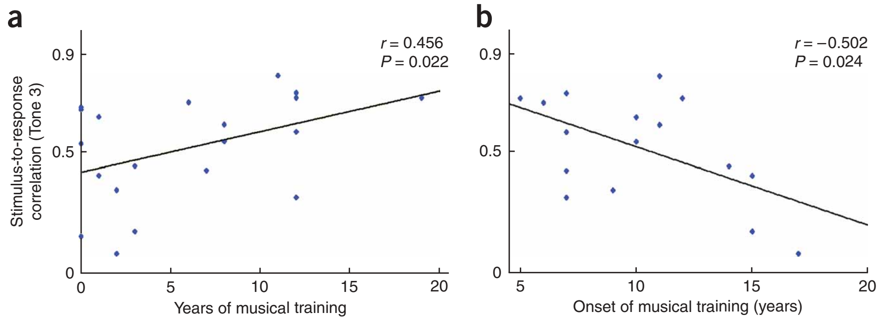

Figure 3 Association between musical training and pitch tracking. (**a**,**b**) Correlations between Tone 3 tracking (stimulus-to-response correlation) and years of musicial training (**a**) and age at which musical training began (age onset; **b**).

© 2007 Nature Publishing Group http://www.nature.com/natureneuroscience

knowledge of the brainstem's role in encoding speech[14] and frequency modulation[15] by demonstrating the interplay between music and speech, subcortical and cortical structures, and the impact of long-term auditory experiences. Our findings have implications not only for biomedical sciences, but also for pedagogical principles and general social and educational policies (see **Supplementary Discussion** online for further discussion).

Note: Supplementary information is available on the Nature Neuroscience website.

ACKNOWLEDGMENTS

The authors thank J. Song, B. Williams, J. Alexander, A. Bradlow, T. Nicol, and T. Perrachione for their assistance in this research. This work is supported by Northwestern University and the US National Institutes of Health grant R03HD051827 and R21DC007468 to P.W. and R01DC001510 to N.K, and National Science Foundation grant BCS-0544846 to N.K.

COMPETING INTERESTS STATEMENT

The authors declare no competing financial interests.

Published online at http://www.nature.com/natureneuroscience
Reprints and permissions information is available online at http://npg.nature.com/reprintsandpermissions

1. Rauschecker, J.P. *Curr. Opin. Neurobiol.* **8**, 516–521 (1998).
2. Patel, A.D. *Nat. Neurosci.* **6**, 674–681 (2003).
3. Wong, P.C., Parsons, L.M., Martinez, M. & Diehl, R.L. *J. Neurosci.* **24**, 9153–9160 (2004).
4. Wong, P.C.M., Perrachione, T.K. & Parrish, T.B. *Hum. Brain Mapp.* (in the press).
5. Russo, N.M., Nicol, T.G., Zecker, S.G., Hayes, E.A. & Kraus, N. *Behav. Brain Res.* **156**, 95–103 (2005).
6. Greenberg, S., Marsh, J.T., Brown, W.S. & Smith, J.C. *Hear. Res.* **25**, 91–114 (1987).
7. Krishnan, A., Xu, Y., Gandour, J. & Cariani, P. *Cogn. Brain Res.* **25**, 161–168 (2005).
8. Li, W., Piech, V. & Gilbert, C.D. *Nat. Neurosci.* **7**, 651–657 (2004).
9. Slevc, L.R. & Miyake, A. *Psychol. Sci.* **17**, 675–681 (2006).
10. Suga, N., Gao, E., Zhang, Y., Ma, X. & Olsen, J.F. *Proc. Natl. Acad. Sci. USA* **97**, 11807–11814 (2000).
11. Schon, D., Magne, C. & Besson, M. *Psychophysiology* **41**, 341–349 (2004).
12. Nosofsky, R.M. *J. Exp. Psychol. Gen.* **115**, 39–61 (1986).
13. Ahissar, M. & Hochstein, S. *Trends Cogn. Sci.* **8**, 457–464 (2004).
14. Johnson, K.L., Nicol, T.G. & Kraus, N. *Ear Hear.* **26**, 424–434 (2005).
15. Gordon, M. & O'Neill, W.E. *J. Comp. Neurol.* **426**, 165–181 (2000).

© 2007 Nature Publishing Group http://www.nature.com/natureneuroscience
