# Supplementary material for: Neural Processing of Speech Sounds in ASD and First-Degree Relatives
Source: J Autism Dev Disord. 2022 Jun 7;53(8):3257–71. doi: 10.1007/s10803-022-05562-7 (PMC10019095; doi:10.1007/s10803-022-05562-7)
Supplement: Supplementary file 62 — Supplementary file62 (PDF 2940 kb) [file 10803_2022_5562_MOESM62_ESM.pdf]

Auditory Brain Stem Response to Complex Sounds: A Tutorial

Erika Skoe[1] and Nina Kraus[1,2]

This tutorial provides a comprehensive overview of the methodological approach to collecting and analyzing auditory brain stem responses to complex sounds (cABRs). cABRs provide a window into how behaviorally relevant sounds such as speech and music are processed in the brain. Because temporal and spectral characteristics of sounds are preserved in this subcortical response, cABRs can be used to assess specific impairments and enhancements in auditory processing. Notably, subcortical auditory function is neither passive nor hardwired but dynamically interacts with higher-level cognitive processes to refine how sounds are transcribed into neural code. This experience-dependent plasticity, which can occur on a number of time scales (e.g., life-long experience with speech or music, short-term auditory training, on-line auditory processing), helps shape sensory perception. Thus, by being an objective and noninvasive means for examining cognitive function and experience-dependent processes in sensory activity, cABRs have considerable utility in the study of populations where auditory function is of interest (e.g., auditory experts such as musicians, and persons with hearing loss, auditory processing, and language disorders). This tutorial is intended for clinicians and researchers seeking to integrate cABRs into their clinical or research programs.

(Ear & Hearing 2010;31;1–●)

INTRODUCTION

The human soundscape is characterized by complex sounds with rich harmonic structures, dynamic amplitude modulations, and rapid spectrotemporal fluctuations. This complexity is represented by an exceptionally precise temporal and spectral neural code within the auditory brain stem, an ensemble of nuclei belonging to the efferent and afferent auditory systems. Within the brain stem, two broad classes of time-locked responses can be defined, namely, transient and sustained. As the names suggest, brief, nonperiodic stimulus features evoke transient responses, whereas periodic features elicit sustained phase-locked responses (Fig. 1). Discovered nearly 40 years ago (Jewett et al. 1970; Jewett & Williston 1971; Moushegian et al. 1973), auditory brain stem responses (ABRs) can be measured using scalp electrodes that pick up electrical potentials generated by the synchronous activity of populations of neurons in the brain stem. Because these aggregate neural responses can be recorded objectively and passively, they offer an excellent means to assess auditory function in a clinical setting. It is for this reason that the click-evoked ABR has enjoyed wide-scale clinical use as a metric for determining auditory thresholds and detecting neuropathologies (Sininger 1993; Starr et al. 1996; Hood 1998; Hall 2006).

Departments of [1]Communication Sciences and [2]Neurobiology and Physiology, Otolaryngology; Northwestern University, Evanston, Illinois. Auditory Neuroscience Laboratory www.brainvolts.northwestern.edu.

Supplemental digital content is available for this article. Direct URL citations appear in the printed text, and links to the digital files are provided in the HTML text of this article on the journal's Web site (www.ear-hearing.com).

Early studies of the ABR used simple stimuli such as clicks and sinusoidal tones to tap into and maximize these transient and sustained ABRs. Although clicks and tones have been instrumental in defining these basic response patterns, they are poor approximations of the behaviorally relevant sounds that we encounter outside the laboratory (e.g., speech and music, nonspeech vocal sounds, and environmental sounds). Moreover, although complex sounds include both sustained and transient features, the response to a complex sound is not necessarily predictable from the response to click and tones (Palmer & Shamma 2004; Song et al. 2006; Johnson et al. 2008a). For these reasons, auditory neuroscience has gradually transitioned to using sounds that are more complex.

Greenberg (1980) was one of the first to adopt complex stimuli for recording ABRs. After the seminal work by Young and Sachs (1979) showing that speech formants are preserved in the discharge pattern of the auditory nerve, Greenberg (1980) observed that speech-specific information (i.e., vowel formants) is also faithfully encoded in the ABR. This stimulus fidelity was further supported by Galbraith et al. (1995), who demonstrated that cABRs to words can be heard as intelligible speech when converted from a neural signal into an audio signal (refer to supplemental audio files). Importantly, because cABRs occur several milliseconds after the stimulus onset, they reflect a response of neural origin and not the cochlear microphonic (CM) (Galbraith et al. 1995), which occurs nearly simultaneous with the stimulus onset (see cABR Collection section for more details on the CM).

A plethora of complex stimuli has now been used to examine how the temporal and spectral features of sounds are preserved in the ABR (Table 1). The two most extensively studied are the consonant-vowel (CV) syllable /dɑ/ (Cunningham et al. 2001; Plyler & Ananthanarayan 2001; King et al. 2002; Russo et al. 2004, 2005; Wible et al. 2004, 2005; Kraus & Nicol 2005; Musacchia et al. 2007; Johnson et al. 2008a; Banai et al. 2009; Burns et al. 2009; Parbery-Clark et al. 2009a; Hornickel et al. 2009b; Chandrasekaran et al. 2009) and Mandarin syllables with differing pitch contours (i.e., lexical tones) (Krishnan et al. 2004, 2005, 2009b; Xu et al. 2006; Wong et al. 2007; Song et al. 2008; reveiwed by Krishnan & Gandour 2009). The ABR to /dɑ/ has been investigated under different recording conditions: monaural (Cunningham et al. 2001; Banai et al. 2009) and binaural (Musacchia et al. 2008; Parbery-Clark et al. 2009a) stimulation; left ear and right ear stimulation (Hornickel et al. 2009a); audiovisual and auditory-only stimulation (Musacchia et al. 2006, 2007); and in the presence of background noise (Cunningham et al. 2001; Russo et al. 2004, 2005, 2008; Parbery-Clark et al. 2009a). Moreover, in addition to manipulating stimulus parameters (e.g., duration of the stimulus, duration of the formant transition, and formant frequency settings), cABRs to /dɑ/ have been evaluated before and after auditory training (Russo et al. 2008b; Song et al.

0196/0202/10/3103-0001/0 • Ear & Hearing • Copyright © 2010 by Lippincott Williams & Wilkins • Printed in the U.S.A.

Copyright © Lippincott Williams & Wilkins. Unauthorized reproduction of this article is prohibited.

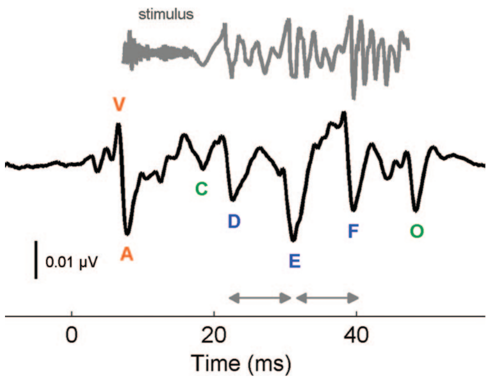

Fig. 1. Transient and sustained features in response to /dɑ/. Time domain representation of a 40 msec stimulus /dɑ/ (gray) and response (black). The cABR to /dɑ/ includes both transient and sustained response features. This stimulus evokes seven characteristic response peaks that we have termed V, A, C, D, E, F, and O. As can be seen in this figure, these peaks relate to major acoustic landmarks in the stimulus. Peaks occur ~7 to 8 msec after the corresponding stimulus landmark, which is consistent with neural transmission time between the cochlea and rostral brain stem. In this figure, the stimulus waveform is shifted in time to account for this transmission time and to maximize the visual coherence between the two signals. The V-A complex, often referred to as the onset response, is analogous to the click-evoked wave V-Vn complex. This sharp onset response arises from the broadband stop burst associated with /d/. Along with V and A, C and O are considered transient responses because they correspond to transient stimulus features, the beginning and end of voicing, respectively. The region bounded by D and F forms the frequency following response. Peaks D, E, and F and the small voltage fluctuations between them correspond to sustained stimulus features, namely the fundamental frequency (F_0) and its harmonics within the consonant-vowel formant transition. The D-E and E-F interpeak intervals (~8 to 9 msec duration, arrows) occurs at the period of the F_0 of the stimulus, which ramps from 103 to 125 Hz. We have developed a systematic approach for identifying these peaks and have established normative data for 3- to 4-yr olds, 5- to 12-yr olds, and young adults (Johnson et al. 2008b; Dhar et al. 2009). Here, and in all figures showing a stimulus waveform, the stimulus plot is scaled to match the size of the response. Hence, the microvolt bar refers only to the response.

2008); across the lifespan (Johnson et al. 2008b; Burns et al. 2009; Anderson et al. 2010); and in a number of different populations including musicians (Musacchia et al. 2007, 2008; Parbery-Clark et al. 2009a) and children with dyslexia, specific language impairment, and autism spectrum disorders (Cunningham et al. 2001; Banai et al. 2005; Banai & Kraus 2008; Banai et al. 2009; Hornickel et al. 2009b; Chandrasekaran et al. 2009; Russo et al. 2009). Similarly, ABRs to syllables with Mandarin pitch contours have been studied from numerous perspectives, including in native and nonnative Mandarin speakers (Krishnan et al. 2005; Xu et al. 2006); in musicians and nonmusicians (Bidelman et al. 2009; Wong et al. 2007); before and after auditory training (Song et al. 2008); under speech and nonspeech conditions (i.e., Mandarin and musical pitch contours embedded in iterated rippled noise) (Bidelman et al. 2009; Swaminathan et al. 2008; Krishnan et al. 2009a); and using native (curvilinear; Krishnan et al. 2005) and non-native (linear; Xu et al. 2006) Mandrin pitch contours (Table 1).

The list of stimuli used to evoke cABRs extends beyond speech syllables and includes words and phrases (e.g., "car," "minute," "chair," "rose" [Galbraith et al. 1995], "dani" (Wang et al. 2010), and "chicken pot pie" [Galbraith et al. 2004]). Investigators have also started to explore the use of environmental sounds, affective nonspeech vocal sounds (e.g., a baby's cry; Strait et al. 2009b) and musical sounds as viable stimuli for brain stem-evoked recordings. Work on music-evoked ABRs has included a bowed cello note (Musacchia et al. 2007, 2008), a five-note musical melody (Skoe & Kraus 2009), as well as consonant and dissonant two-note intervals synthesized from an electric piano (Lee et al. 2009) and tone complexes (Greenberg et al. 1997; Bidelman & Krishnan 2009). Despite being a relatively new endeavor, we anticipate that this arena of research will experience a surge in the upcoming years.

The study of cABRs is a young science. There are many more stimuli, populations, and experimental paradigms yet to be explored. Although interest in this topic is growing, most of the existing cABR research has come from a handful of laboratories around the world. Each laboratory has taken a slightly different approach to collecting and analyzing cABRs, and although the exact methodologies may differ, this work has led to a complete rethinking of what the human ABR represents and how it can be used to study auditory function. Taken as a whole, this work demonstrates that cABRs provide an objective and noninvasive means for studying auditory function in expert (e.g., musicians, native speakers), typically developing, and impaired populations (e.g., persons with hearing loss, auditory processing disorders, and language impairments). Perhaps most crucially, this work has revealed that subcortical processing of sound is not hardwired. It is instead malleable with experience and inextricably linked to cognitive functions involving language and music. This retuning of subcortical function likely involves the corticofugal pathway, an expansive tract of efferent connections that are even more abundant than afferent connections (Galbraith and Doan 1995; Krishnan et al. 2005; Banai & Kraus 2008; see Banai et al. 2009 and Kraus et al. 2009 for treatments of language and music work, respectively, and Tzounopoulos and Kraus 2009 for a review of experience-dependent processes).

It should be pointed out that we use the term "auditory brain stem response" to describe both transient and sustained responses originating in the auditory brain stem (see Chandrasekaran & Kraus 2009 for an in depth discussion of the origins of the cABR). Because the acronym ABR has been widely adopted to refer to click-evoked ABRs, ABRs to complex sounds are often differentiated by other names including speech-ABR or music-ABR. However, because this tutorial focuses on complex sounds in a more general sense that includes nonspeech vocal sounds and environmental sounds, we adopt the phrase complex-ABR or cABR when referring to subcortical responses evoked by complex sounds of any variety.

This tutorial, which represents nearly a decade of accumulated knowledge, was written to encourage researchers and clinicians to adopt cABRs into their clinical or research programs. To help answer frequently-asked questions and to prevent often-encountered stumbling blocks, we provide a comprehensive overview of our systematic approach to stimulus selection, data collection, and analyses. Although we primarily focus on the methodologies used in our laboratory, we also discuss alternative approaches that others have found successful. Because there are a number of systems on the market that can be used to collect evoked potentials (EPs), we frame this tutorial generally and do not provide instructions that are specific to a particular system. More advanced topics are covered in footnotes and figure captions.

Copyright © Lippincott Williams & Wilkins. Unauthorized reproduction of this article is prohibited.

TABLE 1. Survey of speech stimuli used to evoke cABRs

Speech Syllable	Examples	Publications
Vowels		
Synthetic	/ɑ/, /u/	Krishnan 2002
Natural	/ɛ/, /ɪ/, / i/, /ɑ/, /æ/, /ʌ/, /u/	Greenburg et al. 1980; Dajani et al. 2005, Aiken & Picton 2006, 2008
Consonant-vowel syllables		
Synthetic	/dɑ/	Cunningham et al. 2001; Plyler & Ananthanarayan 2001; King et al. 2002; Wible et al. 2004, 2005; Russo et al. 2004, 2005; Kraus & Nicol 2005; Johnson et al. 2007, 2008; Banai et al. 2005, 2009; Burns et al. 2009; Chandrasekaran et al. 2009; Parbery-Clark et al. 2009a
	/bɑ/	Akhoun et al. 2008a,b
	bɑ-dɑ-gɑ continuum	Plyer & Ananthanarayan 2001; Johnson et al. 2008; Hornickel et al. 2009b
Natural	Mandarin pitch contours	
	/yi/	Krishnan et al. 2005; Xu et al. 2006
	/mi/	Wong et al. 2008; Song et al. 2008
Hybrid	/yɑ/ with linearly rising and falling pitch contours	Russo et al. 2008

Recommended background reading • This tutorial serves as a companion to recent review articles produced by our laboratory (Kraus & Nicol 2005; Banai & Kraus 2008; Chandrasekaran & Kraus, Reference Note 1; Kraus et al. 2009; Tzounopoulos & Kraus 2009). If you are not trained in electrophysiology, we suggest that you review the literature on the electrophysiological responses to clicks and tones, because this work formed the foundation for cABR research and still guides our interpretation and analysis today. Also, because ABRs to complex sounds are recorded using essentially the same data acquisition procedures as ABRs to click and tones, and because many of the experimental considerations are also shared, we abbreviate our treatment of these topics and refer the reader to the following resources where these subjects are explored in great depth: Hood (1998), Hall (2006), Burkard et al. (2007), and Krishnan (2007).

ROADMAP

Clinical Considerations
Stimulus Selection and Creation
Stimulus Presentation
- Intensity
- Monaural and binaural stimulation
- Left and right ear stimulation
- Stimulus polarity
- Presentation rate
- Transducer
- Detecting stimulus jitter
- Multiple stimulus conditions

cABR Collection
- Electrodes and electrode montage
- Filters
- Sampling rate
- Signal averaging
- Simultaneous cABR-cortical EP recordings
- Avoiding, detecting and eliminating artifact
- Active versus passive test conditions

Data Analysis
- Analyzing transient responses
  - peak latency and amplitude
  - differences in latency over time
- Analyzing sustained responses
  - static and sliding window analyses
  - RMS amplitude
  - cross-correlation
  - autocorrelation
  - Fourier analysis

Summary
Conclusion

CLINICAL CONSIDERATIONS

Brain stem responses to complex sounds are well suited for clinical applications. In addition to being sensitive biological markers of maturation (Anderson et al. 2010; Johnson et al. 2008a; Burns et al. 2009) and auditory training (Russo et al. 2005; Song et al. 2008), cABRs are highly replicable across test sessions and reliably measured under passive conditions using a small number of electrodes (Russo et al. 2004, 2005). By providing information about the biological basis of hearing and language disorders, cABRs can also help to identify those individuals who are most likely to benefit from auditory training (Hayes et al. 2003; Russo et al. 2005). Thus, in the assessment of hearing and language function, cABRs complement existing technologies, such as click-ABRs, distortion product otoacoustic emissions (Elsisy & Krishnan 2008; Dhar et al. 2009), and behavioral tests of language and auditory processing, including tests of speech in noise (SIN) perception.

Our work with clinical populations has revealed a link between cABRs and higher-level language processes such as reading and SIN perception (Banai et al. 2009; Hornickel et al. 2009b; Chandrasekaran et al. 2009; Parbery-Clark et al. 2009a). For children with language-based learning and reading impairments, brain stem deficits are specific to the fast spectrotemporal elements of speech (Cunningham et al. 2001; King et al. 2002; Wible et al. 2004, 2005; Banai et al. 2005, 2009; Johnson et al. 2007; Hornickel et al. 2009b). This is in contrast to the more pervasive encoding deficits seen in children with autism, which also include abnormal subcortical pitch tracking (Russo et al. 2008b, 2009). Subcortical responses to speech also show a longer developmental trajectory than click-ABRs (Johnson et al. 2008a), suggesting that cABRs could provide an objective index in the early identification of auditory processing deficits that

Copyright © Lippincott Williams & Wilkins. Unauthorized reproduction of this article is prohibited.

lead to learning or literacy disorders. This work with clinical populations led to the development of BioMARK (Biological Marker of Auditory Processing; Natus Medical http://www.natus.com/; see also http://www.brainvolts.northwestern.edu under "Clinical Technologies"), a clinical measure of speech-sound encoding.

In clinical practice, poor speech perception in noise is a commonly encountered complaint. Although everyone experiences reduced speech perception in noise, children with auditory processing disorders and language-based learning impairments, older adults, and individuals with sensorineural hearing loss often experience excessive difficulty in suboptimal listening situations (Dubno et al. 1984; Pichora-Fuller et al. 1995; Kraus et al. 1996; Bradlow et al. 2003; Ziegler et al. 2009). These perceptual findings are reflected in the electrophysiological response when noise (e.g., white noise or multitalker babble) masks the stimulus. In addition to showing that acoustic noise disrupts cABRs in normal populations (Russo et al. 2004), our work has revealed that cABR abnormalities, which can often emerge in responses recorded with masking noise (Cunningham et al. 2001), are linked to poorer SIN perception (Hornickel et al. 2009b; Chandrasekaran et al. 2009). This relationship between subcortical function and speech perception in noise is also evident in musicians who demonstrate better performance on clinical tests of SIN perception relative to nonmusician controls, as well as more robust cABR representation of stimulus features in background noise. Thus, by counteracting the deleterious effects of masking noise (Parbery-Clark et al. 2009a,b; Strait et al, 2009a), musical training may provide a potential remediation strategy for individuals with SIN impairments.

Although cABRs can be used to assess a possible disorder, they do not provide the specificity needed to pinpoint the site of the disorder. This is because an abnormal outcome on a single measure may reflect more than one underlying cause or disorder. The same can be said for click-ABRs (Hood 1998). Thus, no single cABR measure should be considered in isolation when forming a clinical interpretation of the results (see Data Analysis section). However, when interpreted collectively, cABR measures provide both an objective means for delineating the nature of the suspected disorder and an index of training outcome.

STIMULUS SELECTION AND CREATION

Stimulus selection

Stimulus selection should factor in (1) the population being studied, (2) the specific research questions at hand, (3) the electrophysiological properties of the auditory brain stem, and (4) the acoustic features that maximize transient and sustained responses (Table 2).

As a practical guide to stimulus selection, we start with our rationale for selecting /dɑ/ as our primary stimulus. We then describe the transient and sustained aspects of complex stimuli and how they manifest in the cABR. Because there is such a clear transparency between the acoustic features of the stimulus and their subcortical transcription, a basic knowledge of acoustics is necessary for both selecting the stimulus and analyzing the response. Although it is beyond the scope of this tutorial to provide a comprehensive overview, we include in this section brief descriptions of the complex nature of speech and music.

If you are new to cABRs, you are strongly advised to start with stimuli that have been thoroughly characterized (e.g., /dɑ/, vowels, Mandarin pitch contours) to ensure that your collection system is functioning properly (see Stimulus Presentation section). When using novel stimuli, pilot studies are mandatory. During the piloting phase, several different stimulus tokens should be used to determine whether robust and reliable cABRs can be obtained. (For a general overview of the techniques used for detecting and assessing EPs, refer to

TABLE 2. Recommended stimulus and presentation parameters for cABRs

Parameter	Recommendation	Rationale/Comments
Stimulus		
Type	Speech, music, nonspeech vocal sounds, environmental sounds, etc.	Examine how behaviorally relevant sounds are turned into neural code
Characteristics		
Transient	Well-defined temporal features such as strong attacks and amplitude bursts	Maximize transient responses
Sustained	F_0 <300 Hz	Maximize sustained responses
Creation	Natural, synthetic, or hybrid	cABR stimuli can be created with many different software packages
Duration	Short: 40–100 msec<br>Long: >100 msec	Minimizes recording time<br>Maximizes naturalness
Stimulus Presentation		
Intensity	Well above hearing threshold: 60–80 dB SPL	Stimuli should be precisely calibrated before each test session using a sound level meter
Monaural Stimulation	Separate norms should be collected for each ear	Monaural is preferred for children
Binaural Stimulation	Maximizes response characteristics	Binaural is more realistic than monaural
Transducer	Magnetically shielded ear inserts	Minimizes stimulus artifact
Rate and ISI	Rate: dependent on stimulus duration<br>ISI: ≥30% of stimulus duration	See Table 3 for recording-based issues that impact rate and ISI decisions
Presentation Software	Perform thorough testing to ensure precise, nonjittered stimulus presentation	Because of the temporal sensitivity of the cABR, a small amount of jitter will spoil the response

cABRs, auditory brain stem responses to complex sounds; ISI, interstimulus interval.

Copyright © Lippincott Williams & Wilkins. Unauthorized reproduction of this article is prohibited.

Elberling & Don 2007). Tips for maximizing cABRs are provided throughout this section and summarized in Table 2.

Why /dɑ/? • Although we currently use a large repertoire of sounds, our early cABR work focused on the syllable /dɑ/ (Cunningham et al. 2001; Russo et al. 2004), an acoustically complex sound, which begins with a stop burst, characterized by an aharmonic and broadband frication, followed by a harmonically rich and spectrally dynamic formant transition. This CV syllable was chosen for a number of reasons. First, /dɑ/ is a relatively universal syllable that is included in the phonetic inventories of most European languages (Maddieson 1984). Second, the syllable consists of a transient segment followed by a sustained periodic segment. It is, in a sense, much like a click followed by a tone—two acoustic signals whose brain stem response properties have been extensively characterized. Because of these acoustic similarities, the transient onset response to the stop burst is similar to the click-ABR, and the sustained response to the vowel is similar to tone-evoked frequency following response (FFR). Third, stop consonants pose great perceptual challenges to clinical populations such as the hearing and learning impaired (Tallal & Stark 1981; Turner et al. 1992; Kraus et al. 1996). However, because stop bursts are rapid and low in amplitude compared to vowels, even normal-hearing adults and children can find it difficult to discriminate between contrastive stop consonants (e.g., "*d*are" versus "*b*are") in noisy environments. Finally, we continue to use this syllable as our primary stimulus because it elicits clear and replicable ABRs.

Transient features • Transient responses, which are characterized by fast response peaks lasting fractions of milliseconds, are evoked by brief, nonsustained stimulus features such as the onset and offset of sounds. In the case of speech syllables, transient features also include the onset of vocal chord vibration (i.e., voicing) (Fig. 1). For a simple musical stimulus, such as a bowed note of a cello, transient features include the initial burst of sound created by the bow contacting the string and the offset of sound. The morphology of the cABR onset is dictated by the attack characteristics (i.e., how quickly the sound reaches full volume) of the specific acoustic token. Stimuli with sharper rise times (i.e., abrupt onset/amplitude bursts) are more broadband (i.e., less frequency specific) and cause broader and more simultaneous activation of the cochlea, which enlists a larger population of neurons to fire synchronously and leads to more robust (i.e., larger and earlier) transient responses.

For both speech and music, attack characteristics are important for imparting timbre (sound quality), and they contribute to the identification of specific speech sounds (Rosen 1992) and instruments (Grey 1977; Howard & Angus 2001). Within the classes of speech sounds, obstruent stop consonants (e.g., /d/, /p/, /k/) have, by definition, sharper stimulus onsets than nasals and glides (e.g., /m/ and /y/, respectively) and produce more robust onset responses. Although fricatives and affricates have not been used to elicit cABR (to the best of our knowledge), on the basis of stimulus characteristics, we hypothesize the following continuum: earlier and larger onsets for obstruent stops, with affricates (e.g., /tʃ/ pronounced "ch"), fricatives (e.g., /z/), and sonorants (a class of sounds comprising nasals, glides, and liquids; e.g., /r/ and /l/) having increasingly smaller and later onsets. Similarly, when choosing a musical stimulus for eliciting cABRs, the attack properties of the instrument should be taken into consideration. For example, percussive instruments, such as the piano, have fast, steep attacks, and bowed string instruments have comparatively smoother attacks (Fig. 2). Likewise, the mode of playing an instrument affects the attack (e.g., a plucked string has a shorter rise time than a bowed string). In addition, abrupt changes in the amplitude envelope of the sound also trigger onset-like transient responses. For example, Strait et al. (2009b) recorded cABRs to the sound of a baby crying—this particular token included multiple amplitude bursts that produced a series of sharp transient responses (Fig. 2).

Sustained features • Sounds containing continuous acoustic features such as sinusoidal tones, harmonically complex vowels, and musical notes elicit sustained brain stem responses reflecting synchronous population-wide neural phase locking. Moushegian et al. (1973) were the first to describe the sustained response in human scalp-recorded brain stem potentials. Using tones (sinusoids ranging from 250 to 2000 kHz) they demonstrated that each frequency evokes a unique response in which the pattern of neural discharge is time locked to the temporal structure of the eliciting sound. For example, the brain stem response to a 250 Hz tone follows the periodicity of the tone such that response peaks occur at 4 msec intervals (period = 1/frequency; 4 msec = 1/250 Hz). For this reason, sustained brain stem responses are often called frequency following responses. Scalp-recorded FFRs can be recorded to frequencies as high as 1.5 kHz (Moushegian et al. 1973; Krishnan 2002; Aiken & Picton 2008), although phase locking becomes weaker with increasing frequency (Greenberg 1980; Greenberg et al. 1987; Krishnan 2007), reflecting the low-pass nature of brain stem phase locking. Thus, subcortical phase locking provides a mechanism for representing low frequencies contributing to pitch and timbre (Greenberg 1980; Wile & Balaban 2007; Kraus et al. 2009; Bidelman & Krishnan 2009) (Fig. 3), whereas a place code likely underlies the neural encoding of frequencies that are too high to elicit an FFR (Langner & Schreiner 1988; Chandrasekaran & Kraus 2009).

To obtain sustained responses, the cABR stimulus should have a low pitch with a fundamental frequency (F_0) in the range of 80 to 300 Hz. In speech, the F_0 ranges from ~80 Hz for a deep male voice to ~400 Hz for a child. Although speech can contain spectral information up to 10 kHz, the spectral information necessary for distinguishing different consonants and vowels is largely <3 kHz. When selecting a speech phoneme, keep in mind that some speech formants, including the second formant of many vowels, are above the range of brain stem phase locking (Moushegian et al. 1973; Greenberg 1980) and may not be observable in the phase-locked response but may be observable in the timing and the spectral phase of the response (Johnson et al., 2008b; Hornickel et al., 2009b; Skoe et al., 2009; Nicol et al., 2009; Fig. 9).

A wide range of frequencies is also encountered in music. For example, the lowest note on a standard 88-key piano occurs at 32.70 Hz and the highest at 4186 Hz (see Everest 2001 for the frequency ranges of various instruments). Because the F_0s of instruments are often higher than those of speech, they often fall outside the limits of strong brain stem phase locking. Therefore, it may be preferable to use an instrument within this cABR target range, such as the trombone.

Copyright © Lippincott Williams & Wilkins. Unauthorized reproduction of this article is prohibited.

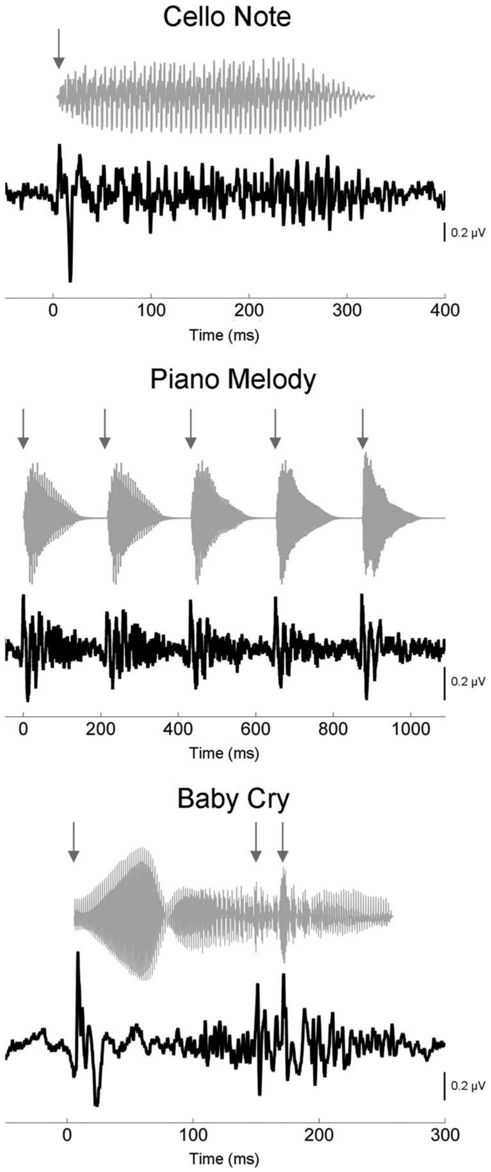

Fig. 2. Transient responses. To maximize the visual coherence between the stimulus (gray) and response (black), stimulus waveforms are shifted in time to align the stimulus with the response onset. Arrows indicate major transient features. In the response, these transient features are represented as large peaks. (Top) The brainstem response to a cello note with a low pitch (G2, 100 Hz). The sound onset occurs when the bow contacts the string and causes a brief transience before the string starts to vibrate in a periodic manner. This leads to a strong onset, followed by a more sustained response. Because of the gradual decay of this sound, a strong offset response is not apparent. For more information see Musacchia et al. 2007. (To listen to the stimulus, go to Audio file, Supplemental Digital Content 1, http://links.lww.com/EANDH/A1; to listen to the response, go to Audio file, Supplemental Digital Content 2, http://links.lww.com/EANDH/A2.) (Middle) Percussive instruments, such as the piano, have fast attacks and rapid decays. These features are evident in this five-note piano melody. Large response peaks coincide with the onset of each piano note. The stimulus amplitude envelope is also preserved in the response. (To listen to the stimulus, go to Audio file, Supplemental Digital Content 3, http://links.lww.com/EANDH/A3; to listen to the response, go to Audio file, Supplemental Digital Content 4, http://links.lww.com/EANDH/A4.) (Bottom) Sounds with abrupt changes in the amplitude envelope also trigger multiple onset-like transient responses. This is illustrated here using the sound of a crying baby. For more information see Strait et al. 2009b. In the top and bottom plots, the stimulus was presented binaurally, and in the middle plot, it was presented monaurally (see Stimulus Presentation section).

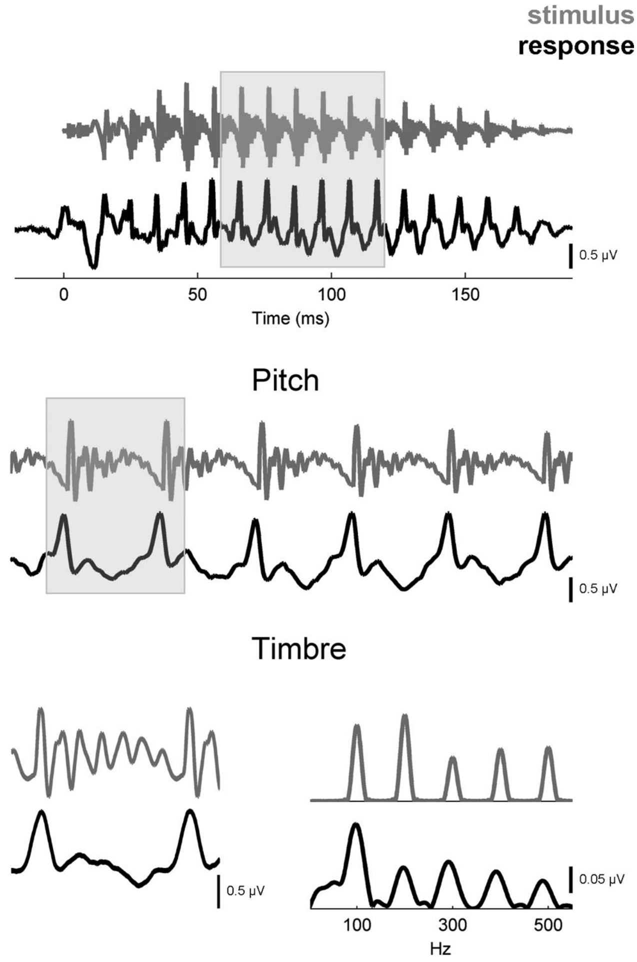

Fig. 3. Sustained phase-locked responses. Low frequencies, including those associated with pitch and timbre perception, are preserved in the auditory brain stem responses to complex sounds (cABR). For complex sounds, the pitch corresponds (in large part) to the lowest resonant frequency, also known as the fundamental frequency (F_0). Timbre enables two sounds with the same pitch to be differentiated. Timbre is a multidimensional property resulting from timing cues of attack and decay, and the interaction of spectral and temporal properties associated with the harmonics of the F_0. These timbral features together give rise to the characteristic sound quality associated with a given instrument or voice. (Top) The full view of the 200 msec time-domain stimulus /dɑ/ (gray) and its cABR (black). The spectro-temporal features of the stimulus, including the F_0 and harmonics, are evident in the response. The gray box demarcates six cycles of the F_0. This section is magnified in the middle panel. (Middle) The smallest repeating unit of the stimulus has a duration of 10 msec (i.e., the periodicity of the 100Hz F_0). (Bottom) The left panel shows a close-up of a single F_0 cycle. The harmonics of the F_0 (frequencies at multiples of 100 Hz) are represented as small fluctuations between the major F_0 peaks in both the stimulus and response. In the right panel, the stimulus and cABR are plotted in the frequency domain.

Time varying and harmonically complex sounds • Real-life sounds, unlike sine waves, have nonstable F_0s and complex harmonic structures. For time-varying stimuli, such as diphthongs, CV formant trajectories, musical glissandos, and linguistic pitch contours, cABRs follow the frequency contour of the stimulus with interpeak intervals systematically increasing or decreasing with changing frequency (changes as small as 1

Copyright © Lippincott Williams & Wilkins. Unauthorized reproduction of this article is prohibited.

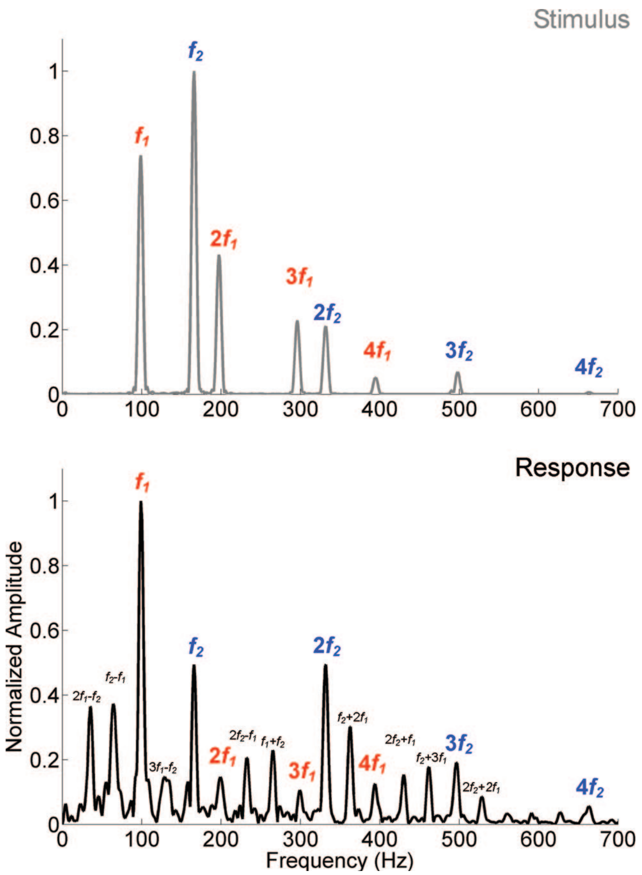

Fig. 4. Distortion products (DPs) in the cABR. Stimulus (top) and response (bottom) spectra for a consonant musical interval (major 6th). This musical stimulus was created from G2 and E3 notes produced by an electric piano. When two harmonically complex notes are played simultaneously, the F_0s and harmonics interact via nonlinear auditory processes to create DPs that are measurable in the response but not present in the stimulus. In this figure, *italics* denote the DPs, f_1 denotes the lower tone (G2, red), and f_2 denotes the upper tone (E3, blue). For more information see Lee et al. 2009 (To listen to the stimulus, go to Audio file, Supplemental Digital Content 5, http://links.lww.com/EANDH/A5; to listen to the response, go to Audio file, Supplemental Digital Content 6, http://links.lww.com/EANDH/A6).

Hz) (Figs. 11 and 13). For harmonically complex sounds, phase locking is observed to the frequencies physically present in the stimulus, and to the frequencies introduced via nonlinear processes within the auditory pathway. Examples include phase locking to the amplitude envelope (Hall 1979; Aiken & Picton 2006, 2008) and distortion products (Elsisy & Krishnan 2008; Abel & Kossl 2009; Lee et al. 2009) (Fig. 4).

Speech • During speech production, sound is produced when air leaving the lungs causes the vocal chords to vibrate. For speech, the F_0 is determined by the rate of this vibration. Because the vocal chords close more slowly than they open, the sound that is produced is not a sinusoid. It is instead a complex waveform resembling a triangle or saw-tooth wave, containing harmonic energy at integer multiples of the F_0. This harmonically-rich sound is then filtered (i.e., shaped) by the speech articulators (i.e., teeth, tongue, lips) to form different speech sounds. Different articulator configurations change the resonance properties of the vocal tract causing certain harmonics to be amplified and others to be attenuated. Formants, which correspond to peaks in the speech spectrum, arise from this filtering. Each speech sound can be uniquely identified by its characteristic formant pattern, with the first two or three formants being sufficient for identifying most speech sounds (Liberman 1954). The cABR, which synchronizes to the F_0 and harmonics of the speech waveform, contains greater energy for harmonics coinciding with formants (Krishnan 2002; Aiken & Picton 2008), because there is more energy in the signal at these frequencies. This has been described as a type of “formant capture” (Young & Sachs 1979; Krishnan 2002), whereby harmonics adjacent to the formant regions are emphasized. Also, note that in the speech spectrum, the F_0 has less energy than the speech formants (Fig. 5). However, because the opening and closing of the vocal folds produces a signal that is naturally amplitude modulated, the F_0 and other modulating frequencies are amplified or introduced into the neural system during nonlinear cochlear processing (Brugge et al. 1969; Regan & Regan 1988; Lins & Picton 1995; Aiken & Picton 2008).

Music • In contrast to speech, which is dominated by fast spectrotemporal transitions, music has more sustained temporal and spectral elements, slower transitions, and finer frequency spacing (Zatorre et al. 2002; Shannon 2005). In music, the mechanism of the F_0 generation depends on the instrument. For example, the reed is the source of the F_0 vibration for the oboe and clarinet, whereas the string is the source for the violin and guitar. In the same way that speech sounds are characterized by unique formant configurations, instruments also have characteristic harmonic structures that impart timbre. Specifically, the timbre of a musical sound is determined by the rise time of the attack (discussed above), the spectral flux (i.e., change in harmonics over time), and the spectral centroid (i.e., the distribution of the harmonics) (Grey 1977). The clarinet, for example, has a harmonic structure dominated by lower frequency odd harmonics (the even harmonics have been attenuated). The flute, saxophone, trombone, and tuba, which are all characterized by strong odd and even harmonics, can be differentiated by the distribution of the harmonics (e.g., the energy of the tuba is concentrated in the lower harmonics).

As can be seen in Figure 5, the harmonic structure of musical sounds is partially preserved in the response. Generally speaking, phase locking is more robust when there is less spectral flux (i.e., brass and woodwind families; Grey 1977). The timbre of a musical instrument also depends on how quickly the sound decays (e.g., a piano has both a fast onset and quick decay, whereas an electric piano has a slower onset and decay). For the purposes of eliciting an FFR, sounds with longer decays elicit responses that are more sustained (Fig. 2). For more information on the rather complex and multifaceted topic of musical timbre refer to Fletcher and Rossing (1991) and Howard and Angus (2001).

Stimulus duration • Within the speech-ABR literature, the length has varied between 60 msec and 2 sec for vowels (Krishnan 2002; Dajani et al. 2005; Aiken & Picton 2006, 2008) and for CVs from 40 to 500 msec (Musacchia et al. 2007; Banai et al. 2009). In experiments using musical stimuli, the duration has ranged from 170 msec for a musical interval (Lee et al. 2009; Fig. 4) to 1.1 sec for a five-note musical melody (Skoe & Kraus 2009; Fig. 2).

Copyright © Lippincott Williams & Wilkins. Unauthorized reproduction of this article is prohibited.

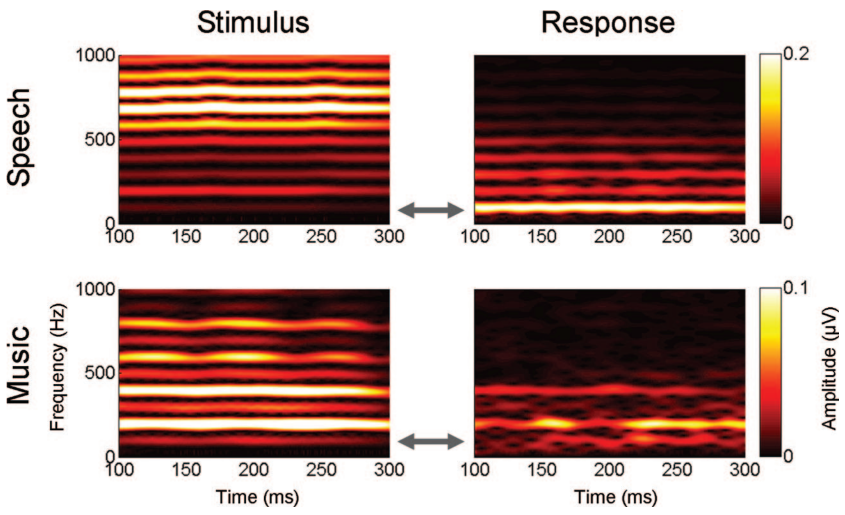

Fig. 5. cABRs to harmonically complex signals. The sustained aspects of cABRs (right) and their evoking stimuli (left) can be visualized using spectrograms (see Data analysis section and Fig. 13). These graphs represent a 200-msec steady-state (unchanging) segment of the vowel /ɑ/ (top) and the cello note (bottom, see also Fig. 2) used in Musacchia et al. 2007. In this example, the speech (top) and musical stimulus (bottom) have the same pitch (F_0 = 100 Hz; arrows), yet have different harmonic structures and consequently different timbres. These acoustic differences account for the different response patterns. For the cello (bottom), the dominant frequency bands occur at 200 and 400 Hz in both the stimulus and response. For the speech signal (top), the harmonics around the first formant (700 Hz) have more energy than the F_0. Yet, lower frequencies dominate the response. This reflects the low-pass nature of brain stem phase locking and the nonlinear processes that amplify the energy of the F_0 and the lower harmonics. For more information see Musacchia et al. 2007.

Because of the sheer number of stimulus presentations required to obtain a robust response, there is an obvious trade-off between stimulus duration and the length of the recording session. For example, to record 6000 trials to a synthesized 40 msec /dɑ/ takes ~9 min, assuming an interstimulus interval (ISI) of 50 msec. Yet, natural sounds generally occur on the order of seconds and not fractions of seconds, which necessarily requires longer recording sessions. In our experience, the only factor limiting the stimulus duration is the feasibility of having a subject remain still for a long time. Thus, stimulus duration may need to be restricted to present the desired number of stimuli in a reasonable amount of time. For speech syllables, one tactic is to record ABRs to a stimulus containing the consonant and CV transition without a steady-state vowel (Russo et al. 2004; Johnson et al. 2007, 2008a; Banai et al. 2009; Dhar et al. 2009; Hornickel et al. 2009a) (Fig. 1 versus Fig. 10). Because each CV has a unique formant transition, the steady state vowel can be removed with little impact on the percept. In fact, musical timbre and vowel identity can be accurately determined from one to four cycles of the F_0 (Gray 1942; Robinson 1995; Robinson & Patterson 1995) but pitch identification requires at least four cycles (Robinson 1995; Robinson & Patterson 1995). Stimulus duration greatly affects pitch because lower frequencies have longer periods than higher frequencies (e.g., a 20 msec stimulus can have no meaningful frequency representation below 50 Hz).

Stimulus creation

With modern computers, recording natural sounds is relatively simple. The process (ideally) requires a sound-attenuated chamber, a microphone, a high-resolution sound card, and software for recording (e.g., Adobe Audition [Adobe Systems, San Jose, CA], Praat [Boersma & Weenink 2009]) (see Aiken & Picton 2008 and Wong et al. 2007 [supplement] for more details). To ensure that a viable token can be extracted, multiple recordings and, when possible, multiple speakers/instruments should be used. Both natural and synthetic sounds should be created with a high digitization rate (>20 kHz). However, because some stimulus presentation systems require a specific sampling rate, the recordings may need to be resampled. Likewise, when comparing the stimulus and the response via cross-correlation (see Data Analysis section), the two signals must have the same sampling rate. For these reasons, it is best to sample the stimulus recordings at a high rate so that upsampling is not necessary.

Speech • Although natural speech and music tokens are ideal in the sense that they represent real-world sounds, they are inherently more complex, variable, and aperiodic. Consequently, with natural tokens, it is difficult to study how specific physical characteristics are represented at a subcortical level. Having precise control over the stimulus parameters is especially important when multiple stimuli are compared across a single dimension. For example, /bɑ/, /dɑ/, and /gɑ/ can be distinguished based on their differing second formant trajectories (F2) (Liberman 1954; Fig. 9). However, natural utterances of /bɑ/, /dɑ/, and /gɑ/ vary on more parameters than simply F2 (as discussed in Johnson et al. 2008b). In these cases, investigators rely on speech synthesizers like Klatt (1976) to create stimuli with precisely defined time-varying and sustained features.*

In the case of the F_0, programs such as STRAIGHT (Kawahara 2009) and Praat can be used to remove aperiodicities, raise or lower the F_0, or apply a particular time-varying pitch contour (Wong et al. 2007; Russo et al. 2008b). To generate stimuli with pitch contours, hybrid stimuli can be made by manipulating the F_0 of a natural speech token or by combining two natural speech tokens using the PSOLA method (Moulines & Charpentier 1990) in a program like Praat (Boersma & Weenink 2009). See Wong et al. (2007) (supplement) and Russo et al. (2008) for more details.

*Klatt, which can function as both a cascade and parallel synthesizer (Holmes 2001), facilitates the manipulation of dozens of features including duration, output sampling rate, the amplitude of frication, the number of formants, and the frequency and bandwidth of each formant. In our experience, the process of creating a synthetic speech sound with the desired percept requires patience and a lot of trial and error. In addition, although you do have control over many parameters, the output may deviate from the input because of the complex interaction among parameters. To confirm that the stimulus meets the desired specifications, the synthetic sound should be acoustically analyzed in a program such as Praat or Adobe Audition (Adobe Systems, Inc., San Jose, CA).

Copyright © Lippincott Williams & Wilkins. Unauthorized reproduction of this article is prohibited.

Music • Because of the increased prevalence of computer-made music, a large number of tools are currently available to generate music stimuli. The choice of the right tool depends on the desired trade-off between acoustic control and sound naturalness. Strict acoustic control of the stimuli can be best achieved through additive synthesis in programming environments such as MATLAB (The Mathworks, Natick, MA). Acoustic samples of real instruments, which can be found in some music software packages (e.g., Garritan Personnal Orchestra in Finale software; MakeMusic, Inc), are another source for music stimuli. An intermediate solution is to use synthesizers, many of which are available as plugins for music software such as Cubase Studio (Steinburg Media Technologies).†

Other sounds • It can be difficult to construct synthetic sounds with strong affective quality. Thus, natural recordings such as those available from the Center for the Study of Emotions and Attention (University of Florida, Gainesville, FL) can be used to study paralinguistic aspects of sounds (Strait et al. 2009b). Similarly, for environmental sounds, we suggest selecting a stimulus from a corpus of natural sounds (e.g., Series 6000 General Sound Effect Library, a royalty-free CD of environmental sounds; Sound Ideas, Richmond Hill, Ontario, Canada).

STIMULUS PRESENTATION

This section covers topics relating to stimulus presentation, including stimulus intensity, monaural and binaural stimulation, left and right ear stimulation, stimulus polarity, stimulation rate, transducers (i.e., earphones and loudspeakers), jitter in the stimulus presentation, and multiple stimulus conditions. A summary is provided in Table 2.

Intensity

Speech, music, and other complex sounds are typically delivered suprathreshold within the "conversational" range of 60 to 85 dB SPL. Similar to the familiar click-ABR, cABRs are also intensity dependent. This necessitates that the intensity be stable across subjects and recording sessions. Before each test session, the output intensity should be calibrated using a sound-level meter with a coupler that enables the output to be measured directly from the insert earphones (see below).

The effects of increasing intensity have been examined in the cABR literature (Krishnan 2002; Akhoun et al. 2008a). Using a /bɑ/ syllable, Akhoun et al. explored how the timing of the speech-evoked onset response and FFR (elicited by the same stimulus) varied as a function of intensity (0 to 60 dB SL, in 10 dB increments). Consistent with the literature on clicks and tones, both response components showed systematic latency shifts with increasing intensity. However, the FFR peaks showed a steeper latency-intensity function than the onset response, suggesting that the onset response and speech-evoked FFR reflect distinct neural processes (Hoorman et al. 1992). Using a similar design, Krishnan recorded cABRs to steady-state vowels between 55 and 85 dB nHL (in 10 dB increments) and found that the harmonics in the formant range were clearly represented for each intensity. Although the amplitudes of the individual harmonics tended to increase with increasing intensity, the trajectory was not identical for all harmonics, nor was the increase always linear. Taken together, this work suggests that different components of the cABR are distinctively impacted by intensity level.

Monaural and binaural stimulation

It is well established that when a sound is heard with both ears that it is perceived to be louder than when the same sound is presented at the same intensity to just one ear (binaural loudness summation is estimated to be 6 dB). Because the auditory brain stem plays an integral role in binaural processing (reviewed in Moore 1991), binaural interaction effects have been widely studied in the click-ABR and tone-FFR literature (Dobie & Berlin 1979; Ballachanda et al. 1994; Krishnan & McDaniel 1998; Ballachanda & Moushegian 2000). Although similar parametric experiments have not been conducted for complex stimuli, the same principles are assumed to apply. For practical reasons, binaural stimulation is preferable when testing adults not only because it leads to larger and more robust responses but also because it is more realistic in that we usually listen with both ears. However, monaural stimulation is used for individuals with asymmetric hearing thresholds, children and other populations who have difficulty sitting still during testing, or when the subject must attend to another sound.

Left and right ear stimulation

Left and right-ear stimulation produce similar but not identical ABRs (Akhoun et al. 2008b). In fact, the well established right-ear advantage for speech is evident for discrete components of the cABR (Hornickel et al. 2009a). For a review of the click-ABR and tone-FFR literature relating to left vs. right ear stimulation, see Hornickel et al. (2009a).

Stimulus polarity

Periodic sound waves traveling through air consist of alternating regions of compression (i.e., condensation) and decompression (i.e., rarefaction) of air molecules. In a time-amplitude plot of a sound wave, condensation and rarefaction manifest themselves as positive or negative deflections (respectively) from the baseline. Because clicks consist of a single positive or negative deflection, they are defined as either having condensation or rarefaction polarity. However, because periodic sounds oscillate between condensation and rarefaction states, the same terminology is not used. In Figures 6 and 8, we have adopted "A" and "B" to refer to the two different polarities. To convert a stimulus from one polarity to another, the waveform is shifted by 180 degrees (i.e., multiplied by -1).

When collecting cABRs, two different approaches can be followed: (1) record the response to a single-stimulus polarity (Krishnan 2007; Aiken & Picton 2008) or (2) record responses to both polarities and either add (Russo et al. 2004; Akhoun et al. 2008a) or subtract responses (Greenberg 1980; Greenberg et al. 1987; Wile & Balaban 2007; Krishnan 2002) to the two

†Unfortunately, these synthesizers are often black boxes. Although they offer control over certain acoustic features, the sound they provide is not as good as samples of real instruments. For all methods of stimulus generation, the acoustic properties of the stimulus should be checked with a sound analyzer before proceeding with the experiment. Acoustic analyzer software dedicated to music sounds includes the MIR toolbox for MATLAB (http://www.jyu.fi/hum/laitokset/musiikki/en/research/coe/materials/mirtoolbox), the IPEM toolbox for MATLAB (http://www.ipem.ugent.be/Toolbox/), and PsySound3 (http://psysound.wikidot.com).

Copyright © Lippincott Williams & Wilkins. Unauthorized reproduction of this article is prohibited.

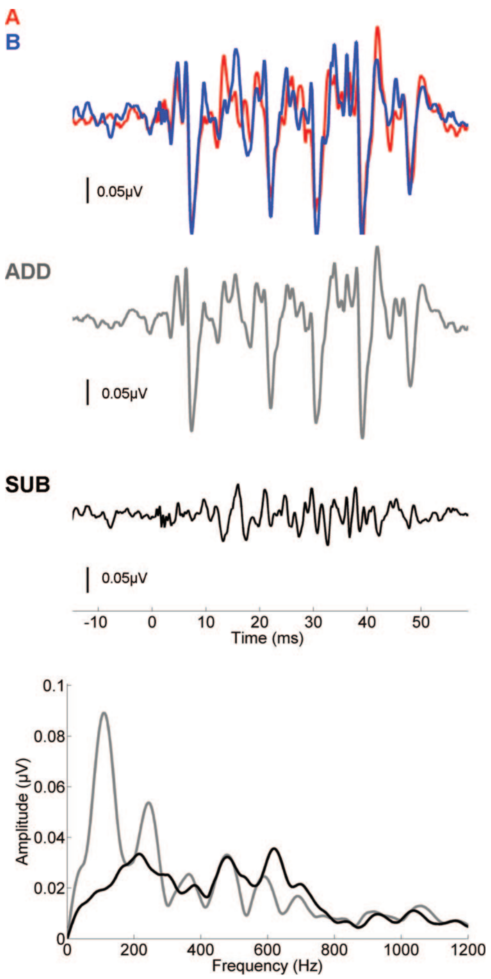

Fig. 6. Stimulus polarities. Responses to the two polarities of the /dɑ/ stimulus from Figure 1. For shorthand, they are referred to as polarity A (red) and B (blue). The cABRs to A and B are quite similar, especially for the prominent negative going peaks corresponding to the onset, offset and F_0 (top). By adding or subtracting A and B, envelope and spectral components of the response, respectively, can be separated (see footnote on page 10). Adding (gray) accentuates the lower frequency components of the response, including the temporal envelope, and minimizes stimulus artifact and the cochlear microphonic (see Fig. 8 and Data Analysis section for a discussion of artifacts). Subtracting (black) emphasizes the higher frequency components by maximizing the spectral response; however, this process can also maximize artifact contamination. In the bottom panel, the ADD and SUB responses are plotted in the frequency domain. In contrast to the ADD response, which has peaks occurring at F_0 (~100 Hz) and the harmonics of the F_0, the SUB response has well-defined peaks in the 200 to 700 Hz range. This range corresponds to the first formant trajectory of this stimulus. In this figure, ADD = (A + B)/2; SUB = (A − B)/2.

stimulus polarities. The process of adding will accentuate the lower-frequency components of the response including phase locking to the amplitude envelope and minimize stimulus artifact and the CM (see Data Analysis section for a further discussion of artifacts). Subtracting will bias the higher-frequency components by maximizing the spectral response, although this process can also maximize artifact contamination. It should be noted that while we use the addition method for many of our published analyses, our results have been internally replicated with single-polarity stimuli. For more advanced considerations of stimulus polarity see Figure 6, footnote ‡, Don et al. (1996), and Aiken and Picton (2008).

Presentation rate

Presentation rate depends on the length of the stimulus and the ISI (defined as the period of silence between the offset of one stimulus and the onset of the next). A second way to express the presentation interval is by defining stimulus onset asynchrony (SOA), which is measured from the onset of one stimulus to the onset of the next. The two measures are essentially the same for click stimulation, as a click virtually has no duration, but ISI and SOA are different for cABRs. In surveying the cABR literature, the ISI has varied from ~30% of the stimulus length to more than double the length.

When choosing an ISI the following considerations should be made. First, changing the ISI can alter the perception of a complex sound. Second, if the ISI is not sufficiently long, the response to one stimulus may not fully conclude before the next stimulus is presented. Thus, the ISI and the duration of the averaging window should be long enough to allow for the response to return to baseline. The ISI should also allow for an ample sample of the baseline (i.e., nonstimulus related) activity so that signal to noise ratios (SNRs) can be evaluated (see Data Analysis section). Third, latencies and amplitudes, particularly of onset responses, are affected by the rate of presentation (Hall 2006). In contrast, it appears that FFR latencies in adults are less susceptible to rate changes than onset responses (Parthasarathy & Moushegian 1993; Krizman et al, Reference

‡Phase locking to spectral energy in the stimulus follows the phase of the stimulus; thus, it inverts when the stimulus polarity is inverted. In Figure 6, this inversion is evident in the regions between the large peaks of the A and B responses. Because of this inversion, adding A and B will theoretically cancel out the spectral response. Subtraction, in contrast, enhances the spectral response (including formant frequencies and temporal fine structure) and attenuates the envelope response. Phase locking to the amplitude envelope is independent of phase because the energy of the envelope is not present in the speech signal but is introduced into the auditory pathway during nonlinear cochlear processing. Thus, phase locking to the amplitude envelope does not invert between A and B and it is, therefore, maximized when the responses to A and B are added. Although the two polarities of an auditory stimulus do not sound different, the two polarities do not elicit identical responses, especially in cases where the stimulus waveform is asymmetric (Greenberg 1980). An advantage to using the addition and subtraction methods is that they represent the average of two polarities, thus relieving the necessity to choose which polarity to use. One criticism of the addition method is that although it will minimize artifacts, it will also create a response at twice the frequency of the actual response (Aiken & Picton 2008). Although this is the case for simple sinusoids, the polarity effects are more complicated for time-varying spectrally complex stimuli. For a complex stimulus, involving multiple frequency components, a polarity reversal will theoretically affect the response latency to a given frequency. This is because the 180-degree inversion will shift the phase of the frequency component by one half-period, resulting in different single unit responses for each polarity. However, because of the complex interaction of temporally dynamic frequency components within a population-wide response, the doubling of frequency for any particular frequency (such as the F_0) is not evident in the cABR, and frequency-dependent latency shifts may be obscured or washed out (Don et al. 1996). Consequently, although the responses to the two polarities are not strictly identical, the differences are not always as extensive as might be predicted. Moreover, although the effects of stimulus polarity have been extensively explored to simple stimuli, the observations based on click stimuli do not generalize to low-frequency tone bursts (Don et al. 1996) nor do the polarity effects for simple stimuli generalize to complex sounds. For a more in-depth description and discussion of these cochlear processes and the effect of stimulus polarity on the click-evoked response, see Hall (2006) and Starr and Don (1988).

Copyright © Lippincott Williams & Wilkins. Unauthorized reproduction of this article is prohibited.

Note 2). Using a speech stimulus, Krizman and colleagues also found that the magnitude of the higher frequency components of the response diminished with increasing rate, but the F_0 did not. Fourth, to avoid contamination from the AC line frequency (60 Hz in North America, 50 Hz elsewhere), a presentation rate should be chosen such that the line frequency divided by the rate is not an integer (e.g., for both 50 and 60 Hz line noise, 10/sec is a bad choice, but 10.3 or 11 is okay). Alternatively, a variable ISI might be used. Fifth, when conducting simultaneous cABR-cortical EP recordings (see Data Analysis section) longer ISIs may be required to obtain robust cortical auditory EPs.

An alternative approach is to record cABRs in several blocks of continuous stimulation (i.e., no silence between stimuli) using the same procedure used to record auditory steady-state responses (Dajani et al. 2005; Aiken & Picton 2006, 2008). This technique maximizes spectral resolution at the expense of temporal resolution.

Transducer

Because circumaural headphones increase the chances for stimulus artifact contamination (see Data Analysis section), we strongly advise against using them and instead recommend electromagnetically shielded insert earphones (e.g., E•A•RTONE3A [Aearo Technologies, Minneapolis, MN], ER-3a [Etymotic Research, Elk Grove Village, IL], Bio-logic insert-earphones [Bio-logic Systems Corp., Mundelein, IL]). When testing persons with hearing aids or other populations not suited for inserts (e.g., cochlear implant wearers), loudspeakers can be used to deliver the stimulus. However, sound field delivery causes the latency of the response to be more variable because the sound intensity changes subtly with head movements. To minimize head movements, we have the subject focus on a movie or another visual image positioned directly in front of him or her. Also, because the intensity is dependent on the distance between the loudspeakers and the subject, we carefully measure and mark the location of the chair and speakers, and position the left and right speakers equidistantly.

Detecting stimulus jitter

One of the defining characteristics of the ABR is that it reflects extremely fast neural activity synchronized across populations of neurons, with minute disruptions in neural precision being indicative of brain stem pathologies (Hood 1998; Hall 2006). For this reason, the delivery and recording units must be precisely time locked to each other.§ Even a small amount of jitter in this synchronization can ruin an ABR recording. If the timing of the stimulus does not always occur at the same time with respect to the triggering of the recording system, the response is canceled, or at the very least distorted, when trials are averaged. Thus, when a new recording system is acquired, it is important to confirm that the delivery system is properly calibrated (see below) to ensure that there is not an unexpected stimulus delay or jitter. A system that has been optimized for collecting cortical responses should also undergo testing before it can be cleared for brain stem testing. Because of timing and duration differences between brain stem and cortical responses, jitter may only be evident when recording brain stem responses.

To determine whether the stimulus presentation is jittered, couple the output of the delivery system into the electrode box, as if recording cABRs from a subject. Next, play a click stimulus and record the output into the recording system in continuous (non-averaging) mode. Adjust the output intensity if the waveform is clipped in the recording. It is important to record a sizeable number of sweeps (100+) to ensure that the jitter does not creep in over time. After the recording is complete, check that each click occurs at the same time relative to the trigger across the recording. For a properly functioning system, the deviation should not exceed 0.1 msec. This is also an opportunity to determine whether the stimulus is actually simultaneous with the trigger or whether there is a delay that needs to be taken into account when processing and analyzing cABRs.¶

Multiple stimulus conditions

When an experiment includes multiple stimulus conditions, a block or interleaved paradigm can be used. In a block paradigm, each condition is presented separately (i.e., block 1: P P P; block 2: Q Q Q) (Johnson et al. 2008b), and in an interleaved paradigm, the stimulus conditions are intermixed (i.e., P Q P Q P Q or P Q P P Q P) (Wong et al. 2007). In the block design, state (i.e., alertness) or expectancy effects may confound comparisons across stimulus conditions. However, if the delivery system is not designed to play multiple stimulus tokens, interleaving stimulus conditions may not be possible. Although the presentation software might limit the number of stimuli that can be interleaved, there does not seem to be a corresponding neurophysiologic limit (e.g., in one experiment, we interleave eight different stimulus conditions, two polarities for each, for a total of 16 different sounds). In the case where multiple stimuli are to be directly compared, it may be desirable to normalize the duration and amplitude across the stimulus set. This can be carried out in programs such as Level 16 (Tice and Carrell, University of Nebraska, Lincoln, NE) and Praat (Boersma & Weenink 2009).

Recent work suggests that block and interleaved designs may invoke different on-line subcortical encoding mechanisms. Chandrasekaran et al. (2009) compared the response to /dɑ/ collected in a block condition and the response to the same stimulus when it was presented with a pseudo-random probability within a mix of seven other speech stimuli. The response to the interleaved condition was found to have smaller spectral amplitudes compared with the block condition, which the authors interpret to be an indication of weaker stimulus "tagging" when the stimulus is presented less frequently.

cABR COLLECTION

Issues relating to electrodes, filtering, sampling rate, signal averaging, simultaneous ABR-cortical EP recording, artifact

§Depending on the recording system, the software used for stimulus delivery is either integrated into the collection system or installed on a separate computer from the collection computer. In the latter case, to synchronize the presentation of stimulus and the recording of the response, the delivery computer sends a digital trigger to the recording computer every time a sound is played.

¶Please note that this testing procedure may not be feasible for every recording system. Because the source of the jitter can come from one or a combination of several sources and because each system will have its own peculiarities, we advise contacting the manufacturer before adjusting any settings.

Copyright © Lippincott Williams & Wilkins. Unauthorized reproduction of this article is prohibited.

TABLE 3. Recommended cABR recording parameters

Parameter	Recommendation	Rationale/Comments
Electrode placement	Vertical montage (active: Cz; reference: earlobe(s); ground: forehead)	For rostral brain stem recordings; a horizontal montage is used for recording from more peripheral structures
Sampling rate	6000–20000 Hz	Better temporal precision with higher sampling rates
Filtering	Low-pass cutoff: 2000–3000 Hz	More defined transient peaks
	High-pass cutoff: 30–100 Hz	Depends on spectral characteristics of stimulus
	If possible, collect cABR with open filters (1–3000 Hz) and band-pass filter off-line using digital filters	Digital filters minimize temporal phase shifts
Signal averaging	2 or more subaverages of 2000–3000 sweeps	Determine response replicability
		Spectral-domain averaging will increase spectral estimates and require fewer sweeps
Averaging window	Begin 10–50 msec before stimulus onset	An adequate sample of the baseline is needed to determine whether a particular response peak is above the noise floor
		For running window analysis, the pre-stimulus time window should be greater than or equal to the duration of the analysis window
	Extend 10–50 msec after stimulus onset	Neural activity should return to baseline
Simultaneous cABR-cortical response recording	Only if large files can be accommodated and longer sessions are appropriate	
Minimizing artifacts	Passive collection protocol	Minimizes myogenic artifacts
	Electromagnetically shielded insert ear phones	Minimize stimulus artifact
	Both stimulus polarities	Enables adding of responses to minimize both stimulus artifact and cochlear microphonic
	Use electrically shielded test booth	Minimizes electrical artifact
	Project movie into test booth	
	Artifact rejection criterion: >20 μV	Exclude trials exceeding typical neural response size; criterion depends on high-pass filter setting

cABRs, auditory brain stem responses to complex sounds.

reduction, and recording conditions are reviewed below and summarized in Table 3.

Electrodes and electrode montage

For cABRs, a vertical one-channel montage is the most common configuration. This configuration requires only three electrodes corresponding to the active (noninverting), reference (inverting), and ground electrodes. In our laboratory, the preferred electrode placements are Cz (active), ipsilateral earlobe (reference), and forehead or contralateral earlobe (ground). We prefer to use the earlobe rather than the mastoid because it is a noncephalic site that causes fewer artifacts from bone vibration (Hall 2006). For researchers who intend to record subcortical and cortical potentials simultaneously (see below) or who wish to collect them within the same session, cABRs can be recorded with an electrode cap.

Filters

Filtering is used to isolate subcortical activity from cortical potentials and to increase the SNR of the response. For cABRs, the band-pass filters match the range of settings used for click-ABRs and typically fall in the range of 100 to 3000 Hz. This frequency range has been found to maximize the detection of the high-frequency transient peaks, such as the click-evoked peaks I-V, which have sharp slopes. For stimuli containing frequencies below 100 Hz (or which produce distortion products below 100; Lee et al. 2009; Fig. 4), the high-pass cutoff should be lowered to ensure that these lower-frequency features are lost. Another approach is to record with more open filters such as 30 to 3000 Hz (Galbraith & Doan 1995; Galbraith et al. 2004).‖

Sampling rate

Sampling rate (Fs), also referred to as the digitization rate, determines how many times per second the neural signal is digitally sampled by the recording system. In cases where only low-frequency components of the response are of interest, a low Fs (1000 to 2000 Hz) may be appropriate (Dajani et al. 2005; Aiken & Picton 2006). However, many researchers opt to over-sample cABR recordings (rates range from 7 to 50 kHz) by sampling well above the Nyquist frequency (i.e., twice

‖A more advanced recording technique uses a two-channel (or more) montage to either simultaneously record horizontal and vertical montages (Galbraith 1994) or to record multiple recording parameters from a single site (e.g., different filter bandwidths; see below). We commonly record in continuous (i.e., nonaveraged) mode using open filters (e.g., 0.1 to 3000 Hz) and then refilter off-line using more narrowly defined digital band-passes. Analog filters, generally used at the time of data collection, are more likely to introduce distortions (e.g., phase distortions) in the response especially when cutoff frequencies are near the frequency range of the response. In addition, filter choice may be restricted by the recording system to only a handful of preset values. Thus, for recording systems that include the option to record with open filters, subsequent digital filtering is preferred. With off-line digital filtering, you have the capability to set cutoff values more precisely and to optimize the filter settings for a particular stimulus by systematically adjusting the band-pass to be more restrictive or more encompassing. However, open filters, because of their susceptibility to cortical activity and noise contamination, can be unsatisfactory for monitoring the quality of the response during acquisition. A two-channel solution, with a second channel using a more restricted band-pass (e.g., 100 to 2000 Hz) for on-line viewing, solves this problem.

Copyright © Lippincott Williams & Wilkins. Unauthorized reproduction of this article is prohibited.

the highest frequency in the stimulus) (Krishnan et al. 2005; Musacchia et al. 2007; Akhoun et al. 2008a; Banai et al. 2009). In addition to reducing sample errors, a higher Fs, by definition, increases the temporal precision of the recording and allows for finer differentiation of response peaks. Because cABR disruptions and enhancements occur on the order of tenths of milliseconds, fine-grained temporal precision is essential. Although a higher frequency is desirable, the choice may be limited by the particular recording system. For example, some recording systems use a fixed number of sample points. In this case, the Fs is dependent on the duration of the recording window (Fs = sample points/duration).

Signal averaging

Number of sweeps • An age-old question in the EP literature is how many sweeps must be averaged to obtain a robust and reliable response. It is well established that for higher intensity stimuli roughly 1000 to 2000 sweeps are needed to collect click-ABRs and tone-FFRs (Hood 1998; Krishnan 2007; Thornton 2007). For cABRs, a comparable but sometimes greater number of sweeps are obtained (1000 to 6000). However, if analyses are only carried out in the frequency domain, then spectral maxima may be detected (i.e., statistically above the noise floor) with fewer sweeps (Dajani et al. 2005; Aiken & Picton 2006, 2008; Chandrasekaran et al. 2009).

Our laboratory takes a conservative approach by collecting more stimulus trials than less, typically ~2000 to 3000 per polarity (i.e., 4000 to 6000 total sweeps). There are several reasons for this strategy. First, this allows for the creation of subaverages that can be used to determine response repeatability and/or track how the response evolves over time. Second, we are often interested in subtle response characteristics and small group differences that may not be apparent until additional sweeps are collected and/or repeatability is confirmed. A general principle of EP signal averaging is that the SNR is proportional to the square root of the number of sweeps (Hood 1998; Hall 2006; Thornton 2007). Thus, the overall SNR increases quickly at first and then begins to plateau with more sweeps. However, an individual component of the cABR (e.g., a specific peak in the time domain or a spectral peak that is near the phase locking limits of the brain stem) may show its own SNR progression with different response components requiring greater or fewer sweeps. Although it may not be possible to determine the "optimal" number of sweeps for a given stimulus and population before the start of an experiment, the optimal range can be deduced a posteriori using an iterative off-line averaging technique based on a handful of subjects from whom a large number of sweeps have been collected (i.e., compare subaverages of 1000 sweeps, 1500 sweeps, 2000 sweeps, . . . 6000 sweeps). In the future, we envision that better characterization of the cABR will enable the number of sweeps to be reduced while still maintaining spectral and temporal precision.

Averaging window • In the time domain, the averaging window should be long enough to include a prestimulus baseline period, the response period, and a poststimulus period. The length of the poststimulus period needs to account for the stimulus transmission delay and neural conduction time. A poststimulus period between 10 and 50 msec is recommended to ensure that the response returns to baseline. The prestimulus baseline reflects the ambient EEG before the response, thereby assisting in the interpretation of the response. For example, when identifying prominent peaks in the response waveform, peak amplitudes are compared with the amplitude of the prestimulus period. For a given peak, if the amplitude does not exceed the baseline amplitude, it is not considered as a valid (i.e., reliable) peak. The baseline period can also be used to determine the SNR (in the time and frequency domains) (see Data Analysis section). For running window analyses (see Data Analysis section), it is helpful to have a prestimulus period that is long enough to include one full analysis window. Because we typically perform running window analyses on 40 msec bins, we use a prestimulus window of at least 40 msec.

Simultaneous cABR—cortical EP recordings

Although we have had success simultaneously recording cABR and cortical responses (Musacchia et al. 2008), there are a number of practical limitations to this practice that arise from cABRs and cortical EPs having different optimal recording parameters. First, cABRs require a much higher Fs than cortical responses (often a 10-fold or more difference) (Burkard et al. 2007). Second, because cortical responses are optimally obtained using slower stimulation rates than ABRs (Burkard et al. 2007), the presentation rate must be slow for simultaneous recordings. Yet, because cABRs are much smaller in amplitude (typically <1 μV), many more trials must be collected for a robust cABR than for a cortical response, often leading to long recording sessions. These factors aggregate to create extremely large files, especially when high-density electrode caps are used, leading to concerns about both computer processing power and data storage. For these reasons, we usually opt to collect brain stem and cortical-evoked responses in separate recording sessions, optimizing recording lengths, numbers of channels, and sampling rates for each.

Avoiding, detecting, and eliminating artifact

There are four types of artifacts that can distort ABR recordings: external (i.e., nonbiological) electrical noise, myogenic (muscular) artifact, CM, and stimulus artifact. Although artifacts can be minimized, it is best to remove the contamination at its source.

Electrical • When combating electrical artifact such as line noise (60 or 50 Hz), the best tactic is to record within an electrically shielded booth and remove all electrical sources from the booth including televisions, and CRT and LCD computer monitors. Light dimmers are another serious source of noise. If the experimenter wishes to play a movie or another visual stimulus during the experiment, two different approaches can be taken. The cheaper option is to use a portable battery-powered DVD player that is placed on a table in front of the subject. The second option, and the one we use most often, is to use an LCD projector located outside the booth that projects the visual stimulus through a booth window onto a screen inside the booth.

Another type of electrical artifact comes from the electrical trigger pulse that is used to synchronize stimulus presentation and response averaging. This artifact appears at time zero. If a long trigger is used, a second artifact may appear when the trigger turns off. If the duration of the trigger pulse can be manually set within the stimulus presentation software, this type of artifact can be reduced by either shortening the trigger

Copyright © Lippincott Williams & Wilkins. Unauthorized reproduction of this article is prohibited.

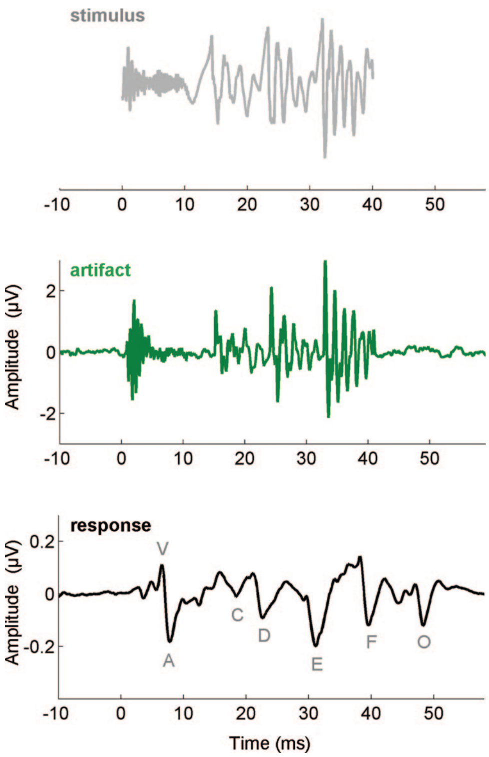

Fig. 7. Detecting stimulus artifact. Stimulus artifacts are easily discernable in the response. Unlike the response (bottom), the artifact (middle) contains frequencies that are higher than the phase-locking capabilities of the brain stem (Moushegian et al. 1973). In contrast to the cABR, which occurs within 6 to 10 msec after the stimulus (top) is played, the stimulus artifact exhibits no delay. In addition, the artifact is often larger than a typical cABR. In this example, the artifact to a 40 msec /dɑ/ (Fig. 1) is 10 times larger than the response. Stimulus artifact can be minimized by using electromagnetically shielded insert earphones and adding the responses to alternating polarities (Fig. 8).

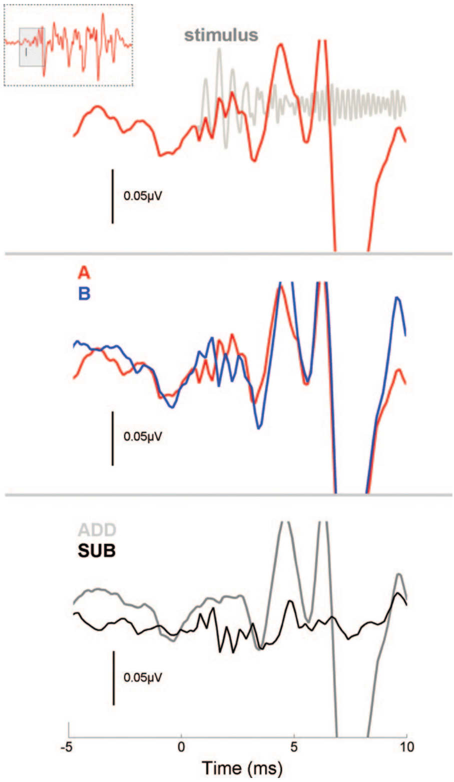

Fig. 8. Adding polarities (A and B) minimizes stimulus artifact and cochlear microphonic. Responses to the A and B polarities of the 40-msec /dɑ/ from Figure 1. (Top) The response to polarity A (inset) is magnified (−5 to 10 msec) to illustrate the stimulus artifact. When the A response (red) is compared with the stimulus (light gray), the two waveforms align in phase for ~4 msec. This is because the stimulus artifact (and CM) follow the temporal pattern of the stimulus waveform. (Middle) The response to polarity B (blue) is inverted with respect to the A response in this region. (Bottom) By adding A and B responses (gray), the artifact is canceled. In contrast, the artifact is accentuated when the two polarities are subtracted (black). Thus, although the analysis of the subtracted waveform or the single polarity response can be complicated by unwanted artifacts, the added response ensures a response of neurogenic origins (Aiken et al. 2008). In this figure, ADD = (A + B)/2; SUB = (A − B)/2.

pulse so it occurs before the onset of the response (e.g., <5 msec), or by making it longer than the stimulus.

Myogenic • Given that cABRs are typically recorded with wide band-pass filters (see above), myogenic artifacts (e.g., neck tension, smiling) are often not filtered out. Because myogenic artifacts produce potentials that can be many times larger than the brain stem response, trials for which the amplitude exceeds a specific threshold should be excluded from the final average (either on-line or off-line). In the cABR literature, this threshold ranges from ±20 to ±75 μV (Galbraith & Doan 1995; Akhoun et al. 2008b). Although this technique removes large artifacts, it does not completely expunge all myogenic contamination from the recording. For this reason, it is important to keep the subject relaxed and still during the recording session.

Cochlear microphonic • The CM is a potential generated by the cochlear hair cells that, similar to the FFR, mimics the temporal waveform of the acoustic stimulus. Because of its similarity to the neural response, care must be taken to prevent or remove the CM from the recordings. The CM can be distinguished from the brain stem response in a number of ways. Unlike the cABR, which occurs at ~6 to 10 msec poststimulus onset, the onset of the CM is nearly coincident with the stimulus. The CM and cABR are also differentially affected by rate, intensity, and noise. For example, although cABRs break down with increases in presentation rate and simultaneous masking intensity, the CM remains unaffected. Furthermore, in contrast to cABR amplitude, which plateaus at suprathreshold levels, the size of the CM usually increases linearly with moderate increases in intensity. For more information on the CM and how it can be isolated from the ABR, we refer the reader to the following studies: Starr and Don (1988), Aiken and Picton (2008), and Chandrasekaran and Kraus (2009).

Stimulus artifact • Given that the cABR occurs within a matter of milliseconds after stimulation, and the fact that the cABR closely mimics the stimulating waveform, stimulus artifact is a major concern. Fortunately, this type of artifact is easy to detect (Fig. 7) and can be minimized with the right recording techniques (Fig. 8).

In most modern EP collection systems, the stimulus waveform is sent as an electrical signal to a transducer where it is converted to an acoustic signal. If the transducer is not properly shielded, the electrical signal can "leak" and get picked up by the electrodes and get recorded by the EP system along with the

Copyright © Lippincott Williams & Wilkins. Unauthorized reproduction of this article is prohibited.

response (Fig. 8). In addition to using electromagnetically shielded earphones (Akhoun et al. 2008a,b), it is good practice to double check that the electrode leads and transducer cables are not touching and to position the electrodes and transducer as far apart as possible. This can be achieved with insert earphones that use a plastic tube to separate the transducer and foam earplug. Using longer tubes or positioning the transducer outside the test booth can further minimize stimulus artifact. For an in-depth discussion of stimulus artifacts in cABR recordings, refer to Aiken and Picton (2008) and Akhoun et al. (2008a,b).

Minimizing the CM and stimulus artifact • Given that both artifacts follow the phase of the stimulus exactly, stimulus artifact and CM can be minimized from the response by adding responses to alternating stimulus polarities (Fig. 8, see Stimulus Presentation section).

Active versus passive test conditions

Because ABRs are not greatly affected by sleep, click-ABRs are often collected while the patient is asleep or sedated. Similarly, to reduce myogenic artifact, many cABR researchers allow or even encourage their subjects to fall asleep on a cot or to recline comfortably in a chair (Dajani et al. 2005; Krishnan et al. 2005; Aiken & Picton 2008). However, to rule out differences in state as a potential confound, our subjects stay awake during testing. To promote relaxation and stillness, subjects watch a movie or read a book. For monaural stimulation, the movie soundtrack is played at a low level (~40 dB SPL), so that it can be heard in the nontest ear without masking the auditory stimulation. Subtitles are displayed for binaural recordings.

cABRs can also be recorded under active test conditions in which the subject performs a task (e.g., detecting/counting oddball stimulus tokens; Musacchia et al. 2007). For example, using an audiovisual paradigm, Musacchia et al. revealed that active multisensory integration can shape how subcortical sensory processes respond to speech and music (Musacchia et al. 2006, 2007). Although there is some disagreement in the literature as to whether attention modulates the click-ABR (Picton & Hillyard 1974; reviewed by Rinne et al. 2008), Galbraith and others demonstrated that attentional state can govern the FFR to tones and speech (Galbraith & Arroyo 1993; Galbraith & Kane 1993; Galbraith & Doan 1995; Galbraith et al. 1998, 2003; Hoormann et al. 2004). This is consistent with recent functional MRI work showing that selective auditory attention tasks can modulate the activation of subcortical structures (Rinne et al. 2008).

Notably, to study the dynamic nature of auditory processing, the subject need not be performing an active task during data collection. A growing body of research supports the use of passive recording conditions to study how brain stem function is fine tuned by experience. Although the subject is not actively processing the sounds evoking the response, cABRs tap into how previous active engagement with sound that has occurred during the course of lifelong or short-term auditory experiences has shaped brain stem processes. This refinement of the sensory system likely results from an interplay between subcortical structures and high-order cognitive processes via the corticofugal system (Banai et al. 2005, 2009; Krishnan et al. 2005; Russo et al. 2005; Musacchia et al. 2007; Wong et al. 2007; Song et al. 2008; Lee et al. 2009; Strait et al. 2009b; reviewed by Kraus et al. 2009; Tzounopoulos & Kraus 2009).

DATA ANALYSIS

To analyze the transient and sustained aspects of cABRs, our laboratory uses a battery of measures to appraise the timing and magnitude of neural synchrony, as well as the strength and precision of phase locking. Because cABRs are rich in temporal and spectral information, the use of multiple measures allows us to (1) dissect individual components of the response and how they reflect distinct aspects of processing and (2) describe brain stem encoding in a holistic manner. Because of the transparency between the temporal and spectral features of the stimulus and the response, our analyses are largely stimulus driven. That is to say, we base our analyses and interpretation on the acoustic make-up of the stimulus. Because of this stimulus-response fidelity, commonly used digital signal-processing tools such as cross-correlation and Fourier analysis can be used to analyze both the stimulus and response. Each of these techniques comes in many variants and each belongs to a large family of analysis methods; however, we generally use each in its most basic form. For more information on digital signal processing, we refer the reader to van Drongelen (2007), Wallisch (2009), and Porat (1997).

This section includes an overview and illustration of the most common signal-processing techniques used to evaluate cABRs, namely peak latency and amplitude measurements, root mean square (RMS) amplitude, cross-correlation, and Fourier analysis. A summary is provided in Table 4. The analyses described below are typically performed off-line on the averaged time-domain response or subaverages. Although some of these measurements can be made directly by the EP collection system, others require the use of computational software packages such as MATLAB. For researchers and clinicians who are not in a position to code their own algorithms, we have developed an open source MATLAB-based toolbox (The Brainstem Toolbox) that is available for free upon request under the GNU General Public License (contact eeskoe@northwestern.edu for more information).

Analyzing transient responses

Peak latency and amplitude • To characterize the transient features of the response, individual peaks relating to major acoustic landmarks in the stimulus are identified (Fig. 1). For each peak, latency (time relative to stimulus onset) and amplitude measurements are obtained. Interpeak measurements are also calculated; these include interpeak amplitude, duration, slope, and area (Russo et al. 2004). In general, transient peaks occur within 6 to 10 msec after the corresponding stimulus landmark. Automated peak-picking algorithms can be used to objectively identify maxima (peaks) or minima (troughs) known to occur within a given latency range. To be considered a reliable peak, the absolute amplitude must be larger than the baseline activity recorded before the onset of the stimulus. Confidence in selection of ambiguous peaks is aided by referring to subaverages. Once the peaks have been identified, they are visually confirmed or rejected by multiple raters who are blind to subject group or stimulus contrasts. When the raters disagree, the selection is determined by the most experienced rater. However, bear in mind that agreement among raters may reflect common training in peak identification. Consequently, if peaks cannot be identified by objective

Copyright © Lippincott Williams & Wilkins. Unauthorized reproduction of this article is prohibited.

TABLE 4. Methods for analyzing cABRs

Method	Description	Rationale/Comments
Transient features		
Peak latency and amplitude	Delineation of transient response peaks	
Sustained features		
RMS amplitude	Global measure of magnitude	Used to calculate SNRs
Fourier analysis	Frequency domain representation	Used to measure the precision and magnitude of neural phase locking at specific frequencies and frequency ranges<br>Amplitude and phase are recorded
Cross-Correlation	Compares the timing and morphology of two signals	Signal 1 is shifted in time relative to signal 2 to find the shift that produces the strongest correlation<br>Examples: stimulus-to-response and quiet-to-noise cross-correlation<br>If the correlation coefficient (r) = 1, the signals are identical. If r = 0, the signals are completely dissimilar
Autocorrelation	A signal is cross-correlated with itself	Used to find (1) repeating patterns in signals such as phase-locked activity to the F_0 and the amplitude envelope and (2) the strength of phase locking
Sliding window analyses	Small time bins (i.e., windows) of the signal are analyzed in succession to create a three-dimensional representation of the response (e.g., spectrograms and autocorrelograms)	Used to evaluate and visualize how cABRs changes over time

cABRs, auditory brain stem responses to complex sounds; RMS, root mean square; SNRs, signal to noise ratios.

methods, an external rater should also be consulted whenever feasible.

A number of techniques have been developed to aid in the identification of difficult to identify/low-amplitude peaks. These include wavelet denoising (Quian Quiroga et al. 2001; Russo et al. 2004) and high-pass filtering (Johnson et al. 2008b; Hornickel et al. 2009b). When determining which peaks to pick in the cABR to a novel stimulus, start by generating a grand average response of all subjects, and then compare the grand average with the stimulus waveform to determine where the two waveforms align. Once this has been performed, the individual waveforms should be reviewed to determine which peaks have high replicability across subjects (i.e., <1 msec deviation across subjects/groups).

Differences in latency over time • When multiple stimulus conditions are compared, a more advanced technique involves calculating how the latency changes between/among conditions as a function of time. For example, recent work from our laboratory (Johnson et al. 2008b; Hornickel et al. 2009b) showed that the formant frequency differences differentiating the stop consonants /bɑ/, /dɑ/, and /gɑ/ are represented by systematic and progressive latency differences in the cABR with /gɑ/ responses occurring first, followed by /dɑ/, and then by /bɑ/ (i.e., higher stimulus frequencies yield earlier response latencies). These latency differences can be visualized using a latency-tracking plot (Fig. 9).

Analyzing sustained responses

Static and sliding window analyses • The response to periodic features (e.g., steady-state vowels, formant transitions, pitch contours, steady-state musical notes, and glissandos) can be analyzed using RMS, cross-correlation, and Fourier analysis. Each of the analysis techniques described below can be used to perform "static" window or "sliding" window (also called running window) analyses. A single region of the time-amplitude waveform is evaluated in a static window

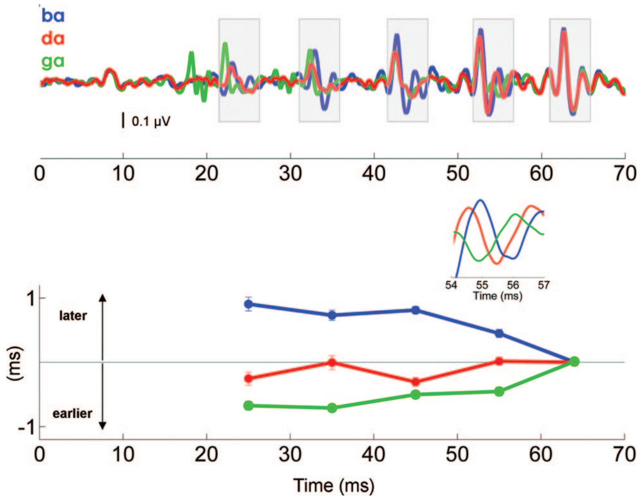

Fig. 9. Tracking latency differences over time. The frequency trajectories that differentiate the consonant-vowel stop syllables /bɑ/, /dɑ/, and /gɑ/ are represented in the responses by latency differences, with /gɑ/ responses occurring first, followed by /dɑ/ and then /bɑ/ (i.e., higher frequencies yield earlier peak latencies than lower frequencies). In the stimulus, the frequency differences diminish during the course of the 50-msec formant transition. (Top) This pattern is reflected in the timing of the cABR (/gɑ/ < /dɑ/ < /bɑ/) and is most apparent at five discrete response peaks. Peaks ~55 msec are magnified in the inset. (Bottom) The normalized latency difference between responses is plotted as a function of time (see Johnson et al. 2008b; Hornickel et al. 2009b, for details).

Copyright © Lippincott Williams & Wilkins. Unauthorized reproduction of this article is prohibited.

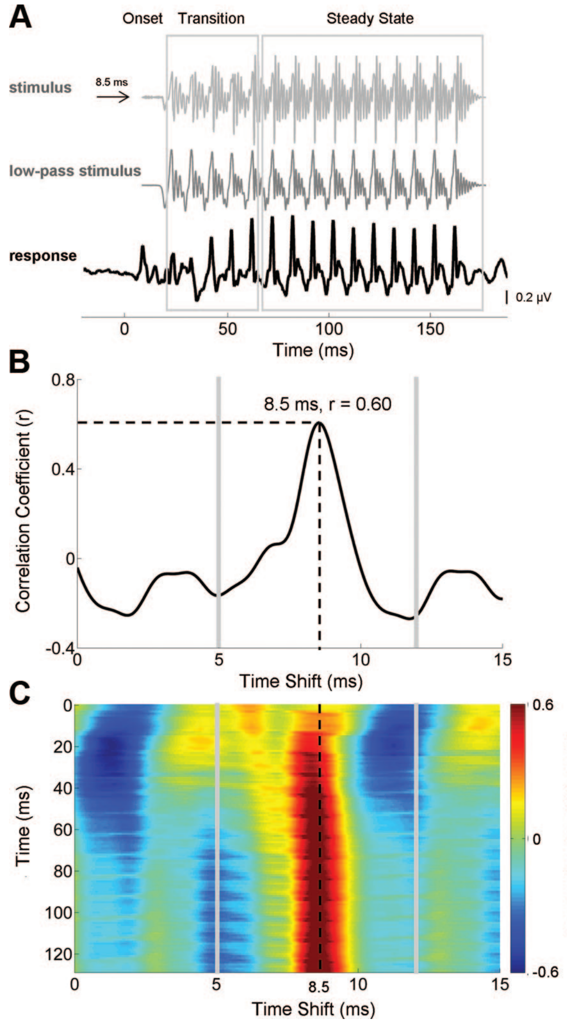

Fig. 10. Stimulus to response cross-correlation. Cross-correlation is used to compare the timing and morphology of two signals (A). The response (black, bottom) to a 170-msec /dɑ/ (gray, top) is compared with a low-pass filtered version (dark gray, middle) of the evoking stimulus. The stimulus consists of an onset stop burst, consonant-vowel formant transition, and a steady-state (i.e., unchanging) vowel. (B) This plot represents the degree to which the low-pass stimulus and response are correlated as a function of the time shift. The maximal correlation is reached at an 8.5-msec time displacement, an indication of the neural transmission delay (r_{max} = 0.60; r_{max} = 0.32 for the unfiltered stimulus [correlogram not shown]). An alternative approach is to cross-correlate the response with the stimulus envelope (Akhoun et al. 2008a), which can often lead to higher correlation values. (C) Running-window analyses can be used to visualize and quantify the similarity of two signals across time. In this example, when the same low-pass stimulus and response are compared in this manner (40 msec windows), the two signals are more similar during the steady-state region, although the delay is consistent across time. (To listen to the stimulus, go to Audio file, Supplemental Digital Content 7, http://links.lww.com/EANDH/A7; to listen to the response, go to Audio file, Supplemental Digital Content 8, http://links.lww.com/EANDH/A8.)

analysis. For sliding-window analyses, small time bins (i.e., windows) of the signal are analyzed in succession. This technique captures how the signal changes over time and it is often used to create a three-dimensional representation of the signal, such as spectrograms (Figs. 5 and 13) and correlograms (Figs. 10 and 11). For time-frequency varying stimuli, such as Mandarin pitch contours, diphthongs, and glissandos, frequency-tracking plots are generated using sliding window analysis (Krishnan et al. 2005; Wong et al. 2007) to capture how the changing F_0 or harmonic is tracked in the response over time.

RMS amplitude • For cABRs, RMS amplitude represents the magnitude of neural activation over a given time period (Russo et al. 2004; Akhoun et al. 2008a). RMS is calculated by (1) squaring each point, (2) finding the mean of the squared values, and then (3) taking the square root of the mean. The quotient of response RMS amplitude (i.e., signal) and prestimulus baseline RMS amplitude (i.e., noise) can serve as a measure of SNR (Russo et al. 2004). If the SNR is <1, the prestimulus activity is larger than the "response" activity. In cases where the SNR of the cABR is <1.5, we recollect the cABR or exclude the subject. A typical cABR has an SNR in the range of 2.5 to 3, although SNRs as high as 6 are not uncommon.

Cross-correlation • Correlation is a useful tool for comparing the overall morphology and timing of two signals (e.g., stimulus versus response; Fig. 10; Russo et al. 2004; Akhoun et al. 2008a). In general terms, cross-correlation determines the extent to which two signals are correlated, as a function of the time shift between them. At a given time displacement, if two signals are identical, the cross-correlation coefficient (r) is 1. If the signals are identical but 180 degrees out of phase, it is -1. However, if the signals are completely dissimilar, $r = 0$. In addition to using cross-correlation to determine the degree of similarity, it can also be used to quantify the time delay between the two signals (i.e., time displacement that produces the greatest r value). The onset of the response can be objectively determined in this manner (Akhoun et al. 2008a) by correlating the stimulus and the response. In addition, two responses can be cross-correlated to determine how much the response has been degraded in noise (Russo et al. 2004, 2005) or how the response changes for different stimulus conditions, for example, left versus right ear stimulation (Hornickel et al. 2009a). When performing stimulus-to-response correlations, the stimulus is low-pass filtered to remove the high frequencies that are not present in the response (Russo et al. 2004; Akhoun et al. 2008a) and then resampled, if necessary, to match the sample rate of the response (see Stimulus Presentation section).

Autocorrelation • Cross-correlation can also be used to find repeating patterns (i.e., periodicities) within a signal, such as the fundamental periodicity (Krishnan et al. 2005; Wong et al. 2007) and the temporal envelope (Lee et al. 2009). This class of cross-correlations is called autocorrelation because a signal is correlated with itself. Autocorrelations are performed by making a copy of a signal and then shifting the copy forward in time (Fig. 11).

The fundamental frequency is represented in the stimulus and cABR by peaks occurring at the period of the F_0 (period = 1/frequency). The interpeak interval (period) can be found by calculating the time shift at which the signal best correlates with itself. Thus, autocorrelation is an objective way for determining interpeak intervals and it can be used to estimate the F_0 of the response (calculated as $1/d$, where d is the time shift needed to obtain the maximum autocorrelation). The strength of phase locking to the F_0 can also be estimated by this maximal correlation coefficient value. In addition, autocorrelation functions that have broader morphology reflect less robust phase-locked responses, and steeper functions reflect sharper and more robust phase locking (Krishnan et al. 2005; Lee et al. 2009).

Copyright © Lippincott Williams & Wilkins. Unauthorized reproduction of this article is prohibited.

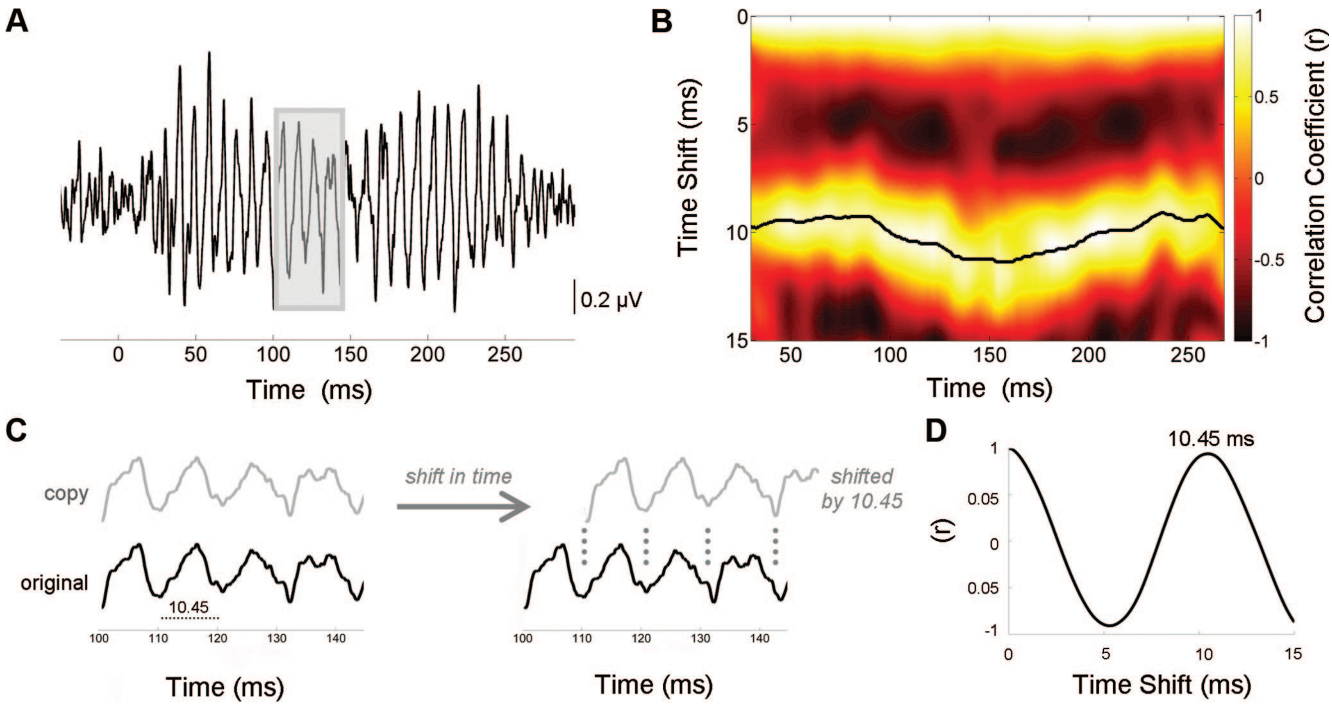

Fig. 11. An illustration of frequency tracking by autocorrelation. By cross-correlating a response waveform with itself, the time interval between peaks can be determined. The frequency of the F_0 and other periodic aspects of the response, including the temporal envelope (Krishnan et al. 2004; Lee et al. 2009), can be derived from an autocorrelogram. This technique can also be used to calculate the strength of phase locking to these features. (A) In this example, the response to a syllable /mi/ with a dipping F_0 contour (Mandarin Tone 3; black line in B) is plotted. (B) By applying the autocorrelation technique on 40-msec sliding windows, a frequency contour can be tracked over time. Colors represent the strength of correlation; white is highest. (C and D) An illustration of cross-correlation performed on a single time window (100 to 140 msec; demarcated in A). When a copy of this window is shifted by 10.45 msec, the first peak of the copy lines up with the second peak of the original (C). A correlogram (D) represents the degree of correlation as a function of the time shift. The highest correlation occurs at 10.45 msec; thus, the fundamental periodicity of this window is 1/10.45 msec or 96 Hz. The strength of the correlation at 10.45 msec is $r = 0.98$, indicating strong phase locking to 96 Hz in this time window.

An autocorrelogram, created via sliding window analysis, is a visual representation of how well the signal correlates with itself across time. In the three-dimensional graph (Fig. 11B), the degree of correlation is represented in color, with the vertical axis representing the time shift and the horizontal axis representing time. Autocorrelograms can be used to evaluate frequency tracking to the F_0 and amplitude envelope. Phase locking can then be described in terms of consistency (i.e., how much the maximum *r* value deviates over time) and strength over time (i.e., the average maximum *r* value over time; Fig. 11; Krishnan et al. 2005; Wong et al. 2007; Lee et al. 2009).

Fourier analysis • A frequency domain representation of the cABR can be generated using Fourier analysis. This method can be used to measure the precision and magnitude of neural phase locking at specific frequencies or frequency ranges. Fourier analyses can be used to generate a frequency-domain average. An alternative, less computationally demanding technique, is to perform an FFT on the time-amplitude average.

One of the basic properties of periodic waveforms is that when two or more waves interact, the resulting waveform is the sum of the individual components (assuming a linear system). This is the principle underlying Fourier analysis. Using Fourier analysis, a complex waveform consisting of many frequency components is decomposed into a set of sine waves. The magnitude of each sine wave corresponds to the amount of energy contained in the complex waveform at that frequency. The spectral composition of a complex wave can then be represented by plotting the frequency of the sine wave on the x axis and the magnitude on the y axis (Figs. 4 and 12). The fast Fourier transform (FFT) (Cooley & Tukey 1965) is the most common algorithm for performing spectral analysis although other Fourier-based methods have been used by cABR researchers (Dajani et al. 2005; Aiken & Picton 2008). The FFT is most efficient (i.e., faster) when the signal N (defined as the

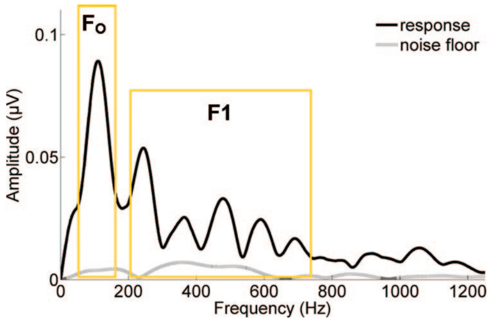

Fig. 12. Fast Fourier analysis of a complex signal with time-varying features. This response was evoked by a 40-msec /dɑ/ sound, comprising an onset stop-burst followed by a consonant-vowel formant transition period. A frequency-domain representation of the frequency following response was generated using the fast Fourier transform (FFT). As a measure of phase locking, spectral amplitudes are calculated over a range of frequencies corresponding to the F_0 (103 to 125 Hz) and the first formant (F1; 220 to 720 Hz). The noise floor is plotted in gray. The time-domain representation of this response is plotted in Fig. 1.

Copyright © Lippincott Williams & Wilkins. Unauthorized reproduction of this article is prohibited.

number of points) is a power of 2. However, software such as MATLAB and Mathematica (Wolfram Research, Inc., Champaign, IL) do not require the input to be a set length.

When dealing with finite signals, such as cABRs, the frequency resolution is dependent on the duration of the sample being analyzed (resolution = $1/T$, where T = duration in seconds). For a 50-msec signal, the frequency resolution is 1/0.05 or 20 Hz. The resulting frequency spectrum contains information only at integer multiples of 20 Hz (i.e., 0, 20, 40, 60. . . Nyquist frequency; 0 Hz = DC component). If the signal contains a frequency component at 130 Hz, the amplitude of the 130 Hz component, which is not an integer multiple of the sampling period, "leaks" into the neighboring components (i.e., 120, 140). This leakage can be reduced by increasing T. As a general rule of thumb, T should be long enough to include a minimum of two to four cycles of the lowest frequency of interest. For example, if you wish to characterize a 100-Hz frequency component, the duration of the signal should be at least 20 msec (i.e., [1/100] × 2). One trick for "increasing" T, without actually taking a longer sample of the signal, is to add a series of zeros to the end of the original sample (often called zero padding; Dajani et al. 2005; Wong et al. 2007). For example, if the 50 msec sample has a 20-kHz sample rate (1000 point sample), to increase the resolution from 20 to 1 Hz, 19,000 zeros are added onto the end of the sample before performing the FFT.

Another thing to consider when performing Fourier analyses is that the FFT treats the sample as if it were a continuous loop in which the first and last samples are contiguous. Thus, if the starting and ending amplitudes are the not same, the amplitude difference gets reflected in the FFT output. When the discontinuity is large, it creates a click-like feature in the response. Because clicks are broadband, this discontinuity results in frequency splattering that contaminates the accuracy of the spectral analysis. To prevent this splatter, a common countermeasure is to multiply the signal by a windowing function, which tapers the amplitudes on both ends so that the sample begins and ends at zero with zero amplitude. Although window functions come in many different shapes, we typically use a Hanning window, which has a bell-shaped function.

For cABRs, frequency spectra are analyzed with respect to the frequency composition of the stimulus. Because stimulus and response amplitudes occur on different scales, the amplitudes must be normalized to plot the two spectra on the same plot. This can be achieved by converting both spectra to decibels (Aiken & Picton 2008) or by dividing each spectral amplitude by the corresponding spectral maximum (Fig. 4; Lee et al. 2009). When analyzing the response in the frequency domain, spectral maxima corresponding to the stimulus F_0 and its harmonics are identified, and the phase and amplitude (modulus of the FFT) of the maxima are recorded. Fourier analysis is also useful for calculating the amplitude over a range of frequencies, especially in cases when the stimulus has time-varying features such as formant transitions (Fig. 12; Banai et al. 2009). By performing an FFT on the prestimulus time window, the spectral noise floor can be estimated (Fig. 12) and used to calculate spectral SNRs.

If performed as part of a sliding-window analysis, the FFT can be used to generate a spectrogram, a three-dimensional graph of the frequency spectrum as a function of time. This type of analysis is often referred to as short-term Fourier transform. In these plots, the horizontal axis (x axis) represents time, the vertical axis (y axis) represents frequency, and the third dimension represents the amplitude at a given time-frequency point. The third dimension is usually represented using a color continuum. Frequency tracks can be derived from short-term Fourier transforms of the response (Fig. 13; Musacchia et al. 2007; Song et al. 2008). Wavelets provide an alternative method for performing time-frequency analyses (see Addison et al. 2009 for an overview of the emerging role of wavelets in biological signal analysis).

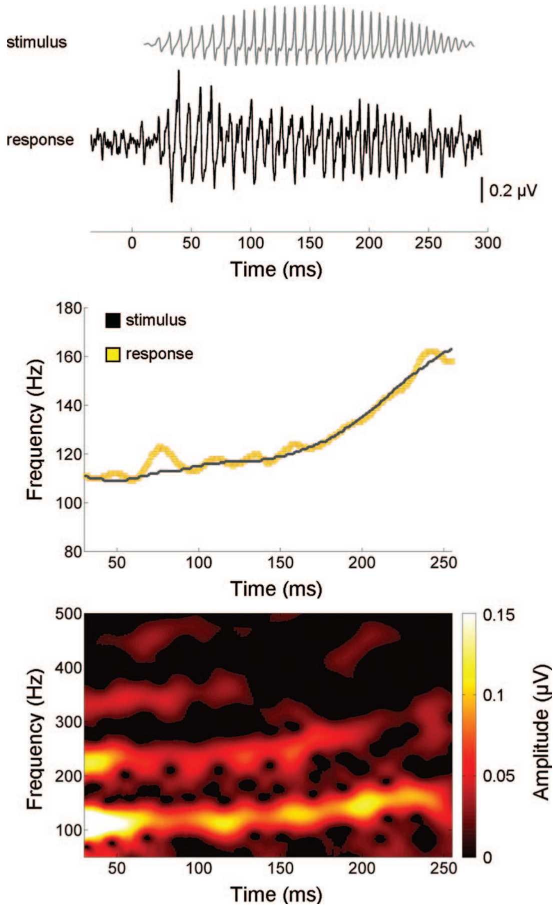

Fig. 13. An illustration of frequency tracking by short-term Fourier transform (STFT) method. STFT is a method for examining frequency tracking, which enables the tracking of the stimulus F_0 and harmonics. (Top) The response (black) to a /mi/ syllable (gray), with a rising pitch contour (Mandarin Tone 2). The rising pitch is evident in the increasingly smaller interpeak intervals in the stimulus and response over time. (Middle) The estimated response F_0 (yellow) contour is plotted against the known F_0 of the stimulus (black). Each point represents the spectral maximum within a single 40-msec window of a sliding-window STFT analysis. The precision of phase locking can be measured by calculating the frequency error between the stimulus and response trajectories (Wong et al. 2007; Russo et al. 2008). (Bottom) Plotting the resulting spectrogram of the STFT procedure enables a visualization of the response's tracking of F_0 and its harmonics. (To listen to the stimulus, go to Audio file, Supplemental Digital Content 9, http://links.lww.com/EANDH/A9; to listen to the response, go to Audio file, Supplemental Digital Content 10, http://links.lww.com/EANDH/A10.)

SUMMARY

1. ABRs provide an objective and noninvasive means for examining how behaviorally relevant sounds such as

Copyright © Lippincott Williams & Wilkins. Unauthorized reproduction of this article is prohibited.

speech and music are transcribed into neural code. The brain stem response is ideal for studying this process because stimulus features are preserved in the response. Notably, this process is not "hard coded." Brain stem encoding of speech and other complex signals is shaped by short-term and lifelong musical and language experience and are thereby tightly coupled with cognitive processes. Aspects of the response are selectively impaired or enhanced in impaired and expert populations (e.g., children with reading impairments and musicians), facilitating the delineation of the underlying neural processes.
2. Similar to click-evoked ABRs, cABRs are well suited for clinical applications because they can be meaningfully applied to individuals.
3. A variety of speech and musical stimuli have been used to evoke ABRs. When choosing a stimulus, the acoustic properties of the stimulus matter. To maximize transient responses, the sound should have sharp onsets or amplitude bursts. A low pitch stimulus (<300 Hz), or a stimulus with its fundamental periodicity in this range, is needed to obtain strong phase-locked (i.e., sustained) responses to the F_0 and its harmonics.
4. cABRs are generally elicited at suprathreshold levels (60 to 80 dB SPL) using monaural or binaural stimulation via electromagnetically shielded insert earphones. If the stimulus presentation is jittered by even a small amount, cABRs are canceled when trials are averaged.
5. cABRs can be recorded using the same data acquisition procedures as click-ABRs and tone-FFRs. Additionally, manipulations of stimulus polarity can be used to enhance different aspects of the response and to minimize stimulus artifacts and the CM.
6. Because of the transparency between the stimulus and response, digital signal processing tools (e.g., cross-correlation, Fourier analysis) can be used to analyze both the stimulus and response. Sliding-window analysis is used to track how the response changes over time.

CONCLUSION

Neural transcription of sound in the auditory brain stem is an objective measure of auditory processing and as such can be applied to research and clinical assessment whenever auditory processing is of interest. This includes the investigation of auditory specialization (e.g., musicians, native language speakers) and the management of auditory disorders (e.g., auditory processing disorders, language-based learning impairments such as dyslexia, specific language impairment, autism, hearing loss, and age-related hearing decline) that often result in pervasive difficulties with speech perception especially in noise. ABRs to complex sounds provide an objective neural metric for determining the effectiveness of remediation strategies, providing the outcome measures that clinicians need to strengthen their role in advocating for auditory training and remediation across the lifespan. Together with converging lines of research (Fritz et al. 2007; Weinberger 2007; Luo et al. 2008; Atiani et al. 2009), the cABR has reinforced the notion that a contemporary view of the auditory system must include its cognitive and sensory functions. That is, subcortical function inherently reflects a confluence of sensory and cognitive processes that likely operate in a reciprocally interactive manner. This view can help the field of audiology more effectively address socially and clinically meaningful aspects of human communication. It is hoped that the methodological information in this tutorial move forward our knowledge and clinical management of auditory processing.

ACKNOWLEDGMENTS

The authors thank Mary Ann Cheatham, Trent Nicol, Bharath Chandrasekaran, and the members of the Auditory Neuroscience Laboratory for their careful review of this manuscript. They extend their gratitude to Alexandra Parbery-Clark and Frederic Marmel for their contributions to the music-related aspects of the Stimulus Selection and Creation section.

This work was supported by NSF 0842376 and NIH R01 DC01510, F32 DC008052.

Co-author Nina Kraus currently serves as consultant to Natus Corp. for BioMARK, a clinical measure of auditory function, and receives royalties through Northwestern University, Evanston, IL.

Address for correspondence: Erika Skoe, Northwestern University, 2240 Campus Drive, Evanston, IL 60208. E-mail: eeskoe@northwestern.edu.

Received June 19, 2009; accepted November 13, 2009.

REFERENCES

Abel, C., & Kossl, M. (2009). Sensitive response to low-frequency cochlear distortion products in the auditory midbrain. *J Neurophysiol*, *101*, 1560–1574.
Addison, P., Walker, J., Guido, R. (2009). Time-frequency analysis of biosignals. *IEEE Eng Med Biol Mag*, *28*, 14–29.
Aiken, S. J., & Picton, T. W. (2006). Envelope following responses to natural vowels. *Audiol Neurootol*, *11*, 213–232.
Aiken, S. J., & Picton, T. W. (2008). Envelope and spectral frequency-following responses to vowel sounds. *Hear Res*, *245*, 35–47.
Akhoun, I., Gallego, S., Moulin, A., et al. (2008a). The temporal relationship between speech auditory brainstem responses and the acoustic pattern of the phoneme /ba/ in normal-hearing adults. *Clin Neurophysiol*, *119*, 922–933.
Akhoun, I., Moulin, A., Jeanvoine, A., et al. (2008b). Speech auditory brainstem response (speech ABR) characteristics depending on recording conditions, and hearing status: An experimental parametric study. *J Neurosci Methods*, *175*, 196–205.
Anderson, S. & Kraus, N. (2010). *Neural Signatures of Speech-in-Noise Perception in Older Adults*. Anaheim, CA: Mid-Winter Meeting for the Association for Research in Otolaryngology.
Atiani, S., Elhilali, M., David, S. V., et al. (2009). Task difficulty and performance induce diverse adaptive patterns in gain and shape of primary auditory cortical receptive fields. *Neuron*, *61*, 467–480.
Ballachanda, B. B., & Moushegian, G. (2000). Frequency-following response: Effects of interaural time and intensity differences. *J Am Acad Audiol*, *11*, 1–11.
Ballachanda, B. B., Rupert, A., Moushegian, G. (1994). Asymmetric frequency-following responses. *J Am Acad Audiol*, *5*, 133–137.
Banai, K., & Kraus, N. (2008). *The Dynamic Brainstem: Implications for APD.* San Diego, CA: Plural Publishing Inc.
Banai, K., Abrams, D., Kraus, N. (2007). Sensory-based learning disability: Insights from brainstem processing of speech sounds. *Int J Audiol*, *46*, 524–532.
Banai, K., Nicol, T., Zecker, S. G., et al. (2005). Brainstem timing: Implications for cortical processing and literacy. *J Neurosci*, *25*, 9850–9857.
Banai, K., Hornickel, J., Skoe, E., et al. (2009). Reading and subcortical auditory function. *Cereb Cortex*, *19*, 2699–2707.
Bidelman, G., & Krishnan, K. (2009). Neural correlates of consonance, dissonance, and the hierarchy of musical pitch in the human brainstem. *J Neurosci, 29*, 13165–13171.
Bidelman, G. M., Gandour, J. T. & Krishnan, A. (2009). Cross-domain effects of music and language experience on the representation of pitch

Copyright © Lippincott Williams & Wilkins. Unauthorized reproduction of this article is prohibited.

in the human auditory brainstem. *J Cogn Neurosci*, DOI:10.1162/jocn.2009.21362.

Boersma, P., & Weenink, D. (2009). Praat: Doing phonetics by computer (version 5.1.05).

Bradlow, A. R., Kraus, N., Hayes, E. (2003). Speaking clearly for children with learning disabilities: Sentence perception in noise. *J Speech Lang Hear Res*, *46*, 80–97.

Brugge, J. F., Anderson, D. J., Hind, J. E., et al. (1969). Time structure of discharges in single auditory nerve fibers of the squirrel monkey in response to complex periodic sounds. *J Neurophysiol*, *32*, 386–401.

Burkard, R. F., Eggermont, J. J., & Don, M. (Eds). (2007). *Auditory Evoked Potentials: Basic Principles and Clinical Application.* Philadelphia, PA: Lippincott Williams and Wilkins.

Burns, K., Soll, A., Vander Werff, K. (2009). Brainstem responses to speech in younger and older adults, American Auditory Society Annual Meeting Scottsdale, AZ, March 2009.

Chandrasekaran, B., & Kraus, N. (2009). The scalp-recorded brainstem response to speech: Neural origins and plasticity. *Psychophysiology.*

Chandrasekaran, B., Gandour, J. T., Krishnan, A. (2007). Neuroplasticity in the processing of pitch dimensions: A multidimensional scaling analysis of the mismatch negativity. *Restor Neurol Neurosci*, *25*, 195–210.

Chandrasekaran, B., Hornickel, J., Skoe, E., et al. (2009). Context-dependent encoding in the human auditory brainstem relates to hearing speech in noise: Implications for developmental dyslexia. *Neuron, 64*, 311–319.

Cooley, J. W., & Tukey, J. W. (1965). An algorithm for the machine calculation of complex fourier series. *Math Comput*, *19*, 297–301.

Cunningham, J., Nicol, T., Zecker, S. G., et al. (2001). Neurobiologic responses to speech in noise in children with learning problems: Deficits and strategies for improvement. *Clin Neurophysiol*, *112*, 758–767.

Dajani, H. R., Purcell, D., Wong, W., et al. (2005). Recording human evoked potentials that follow the pitch contour of a natural vowel. *IEEE Trans Biomed Eng*, *52*, 1614–1618.

Dhar, S., Abel, R., Hornickel, J., et al. (2009). Exploring the relationship between physiological measures of cochlear and brainstem function. *Clin Neurophysiol*, *120*, 959–966.

Dobie, R. A., & Berlin, C. I. (1979). Binaural interaction in brainstem-evoked responses. *Arch Otolaryngol*, *105*, 391–398.

Don, M., Vermiglio, A. J., Ponton, C. W., et al. (1996). Variable effects of click polarity on auditory brain-stem response latencies: Analyses of narrow-band ABRs suggest possible explanations. *J Acoust Soc Am*, *100*, 458–472.

Dubno, J. R., Dirks, D. D., Morgan, D. E. (1984). Effects of age and mild hearing loss on speech recognition in noise. *J Acoust Soc Am*, *76*, 87–96.

Elberling, C., & Don, M. (2007). Detecting and Assessing Synchronous Neural Activity in the Temporal Domain (SNR, Response Detection). In R. F. Burkard, J. J. Eggermont, M. Don (Eds). *Auditory Evoked Potentials: Basic Principles and Clinical Application* (pp, 102–123). Philadelphia, PA: Lippincott Williams & Wilkins.

Elsisy, H., & Krishnan, A. (2008). Comparison of the acoustic and neural distortion product at 2f1–f2 in normal-hearing adults. *Int J Audiol*, *47*, 431–438.

Everest, F. A., & Ebrary Inc. (2001). *The Master Handbook of Acoustics* (4th ed., pp, xix, 615). New York: McGraw-Hill.

Fletcher, N. H., & Rossing, T. D. (1991). *The Physics of Musical Instruments.* New York: Springer-Verlag.

Fritz, J. B., Elhilali, M., David, S. V., et al. (2007). Auditory attention–focusing the searchlight on sound. *Curr Opin Neurobiol*, *17*, 437–455.

Galbraith, G. C. (1994). Two-channel brain-stem frequency-following responses to pure tone and missing fundamental stimuli. *Electroencephalogr Clin Neurophysiol*, *92*, 321–330.

Galbraith, G. C., & Arroyo, C. (1993). Selective attention and brainstem frequency-following responses. *Biol Psychol*, *37*, 3–22.

Galbraith, G. C., & Kane, J. M. (1993). Brainstem frequency-following responses and cortical event-related potentials during attention. *Percept Mot Skills*, *76*, 1231–1241.

Galbraith, G. C., & Doan, B. Q. (1995). Brainstem frequency-following and behavioral responses during selective attention to pure tone and missing fundamental stimuli. *Int J Psychophysiol*, *19*, 203–214.

Galbraith, G. C., Olfman, D. M., Huffman, T. M. (2003). Selective attention affects human brain stem frequency-following response. *Neuroreport*, *14*, 735–738.

Galbraith, G. C., Arbagey, P. W., Branski, R., et al. (1995). Intelligible speech encoded in the human brain stem frequency-following response. *Neuroreport*, *6*, 2363–2367.

Galbraith, G. C., Bhuta, S. M., Choate, A. K., et al. (1998). Brain stem frequency-following response to dichotic vowels during attention. *Neuroreport*, *9*, 1889–1893.

Galbraith, G. C., Amaya, E. M., de Rivera, J. M., et al. (2004). Brain stem evoked response to forward and reversed speech in humans. *Neuroreport*, *15*, 2057–2060.

Gray, G. W. (1942). Phonemic microtomy: The minimum duration of perceptible speech sounds. *Speech Monographs*, *9*, 75.

Greenberg, S. (1980). Wpp, no. 52: Temporal neural coding of pitch and vowel quality. *Working Papers in Phonetics.* Department of Linguistics, UCLA.

Greenberg, S., Marsh, J. T., Brown, W. S., et al. (1987). Neural temporal coding of low pitch. I. Human frequency-following responses to complex tones. *Hear Res*, *25*, 91–114.

Grey, J. M. (1977). Multidimensional perceptual scaling of musical timbres. *J Acoust Soc Am*, *61*, 1270–1277.

Hall, J. W. (2006). *New Handbook of Auditory Evoked Responses.* Boston: Allyn and Bacon.

Hall, J. W., III. (1979). Auditory brainstem frequency following responses to waveform envelope periodicity. *Science*, *205*, 1297–1299.

Hayes, E. A., Warrier, C. M., Nicol, T. G., et al. (2003). Neural plasticity following auditory training in children with learning problems. *Clin Neurophysiol*, *114*, 673–684.

Holmes, J. (2001). *Speech synthesis and recognition*. Danvers, MA: Frances & Taylor Press.

Holmes, J., & Holmes, W. (2002). *Speech Synthesis and Recognition.* Philadelphia, PA: Taylor Francis, Inc.

Hood, L. J. (1998). *Clinical Applications of the Auditory Brainstem Response*. San Diego, CA: Singular Publication Group.

Hoormann, J., Falkenstein, M., Hohnsbein, J., Blanke, L. (1992). The human frequency-following response (FFR): normal variability and relation to the click-evoked brainstem response. *Hear Res,* 59, 179–188.

Hoormann, J., Falkenstein, M., Hohnsbein, J. (2004). Effects of spatial attention on the brain stem frequency-following potential. *Neuroreport*, *15*, 1539–1542.

Hornickel, J., Skoe, E., Kraus, N. (2009a). Subcortical laterality of speech encoding. *Audiol Neurootol*, *14*, 198–207.

Hornickel, J., Skoe, E., Nicol, T., et al. (2009b). Subcortical differentiation of stop consonants relates to reading and speech-in-noise perception. *Proc Natl Acad Sci USA*, *106*, 13022–13027.

Howard, D. M., & Angus, J. A. S. (2001). *Acoustics and Psychoacoustics* (2nd ed.). Oxford, Boston: Focal Press.

Jewett, D. L., & Williston, J. S. (1971). Auditory-evoked far fields averaged from the scalp of humans. *Brain*, *94*, 681–696.

Jewett, D. L., Romano, M. N., Williston, J. S. (1970). Human auditory evoked potentials: Possible brain stem components detected on the scalp. *Science*, *167*, 1517–1518.

Johnson, K. L., Nicol, T. G., Zecker, S. G., et al. (2007). Auditory brainstem correlates of perceptual timing deficits. *J Cogn Neurosci*, *19*, 376–385.

Johnson, K. L., Nicol, T., Zecker, S. G., et al. (2008a). Developmental plasticity in the human auditory brainstem. *J Neurosci*, *28*, 4000–4007.

Johnson, K. L., Nicol, T., Zecker, S. G., et al. (2008b). Brainstem encoding of voiced consonant–vowel stop syllables. *Clin Neurophysiol*, *119*, 2623–2635.

Kawahara, H. (2009). STRAIGHT. Retrieved from http://www.Wakayama-u.Ac.Jp/~kawahara/straighttrial/.

King, C., Warrier, C. M., Hayes, E., et al. (2002). Deficits in auditory brainstem pathway encoding of speech sounds in children with learning problems. *Neurosci Lett*, *319*, 111–115.

Klatt, D. (1976). Software for cascade/parallel formant synthesizer. *J Acoust Soc Am*, *67*, 971–975.

Kraus, N., & Nicol, T. (2005). Brainstem origins for cortical 'what' and 'where' pathways in the auditory system. *Trends Neurosci*, *28*, 176–181.

Kraus, N., & Banai, K. (2007). Auditory processing malleability: Focus on language and music. *Curr Dir Psychol Sci*, *16*, 105–109.

Kraus, N., Skoe, E., Parbery-Clark, A., et al. (2009). Experience-induced malleability in neural encoding of pitch, timbre, and timing. *Ann N Y Acad Sci*, *1169*, 543–557.

Copyright © Lippincott Williams & Wilkins. Unauthorized reproduction of this article is prohibited.

Kraus, N., McGee, T. J., Carrell, T. D., et al. (1996). Auditory neurophysiologic responses and discrimination deficits in children with learning problems. *Science*, *273*, 971–973.

Krishnan, A. (2002). Human frequency-following responses: Representation of steady-state synthetic vowels. *Hear Res*, *166*, 192–201.

Krishnan, A. (2007). Frequency-Following Response. In R. F. Burkard, J. J. Eggermont, M. Don (Eds.). *Auditory Evoked Potentials: Basic Principles and Clinical Application* (pp, 313–335). Philadelphia, PA: Lippincott Williams & Wilkins.

Krishnan, A., & McDaniel, S. S. (1998). Binaural interaction in the human frequency-following response: Effects of interaural intensity difference. *Audiol Neurootol*, *3*, 291–299.

Krishnan, A., & Gandour, J. T. (2009). The role of the auditory brainstem in processing linguistically-relevant pitch patterns. *Brain Lang, 110*, 135–148.

Krishnan, A., Swaminathan, J., Gandour, J. T. (2009a). Experience-dependent enhancement of linguistic pitch representation in the brainstem is not specific to a speech context. *J Cogn Neurosci*, *21*, 1092–1105.

Krishnan, A., Xu, Y., Gandour, J. T., et al. (2004). Human frequency-following response: Representation of pitch contours in Chinese tones. *Hear Res*, *189*, 1–12.

Krishnan, A., Xu, Y., Gandour, J., et al. (2005). Encoding of pitch in the human brainstem is sensitive to language experience. *Brain Res Cogn Brain Res*, *25*, 161–168.

Krishnan, A., Gandour, J. T., Bidelman, G. M., et al. (2009b). Experience-dependent neural representation of dynamic pitch in the brainstem. *Neuroreport*, *20*, 408–413.

Langner, G., & Schreiner, C. E. (1988). Periodicity coding in the inferior colliculus of the cat. I. Neuronal mechanisms. *J Neurophysiol*, *60*, 1799–1822.

Lee, K. M., Skoe, E., Kraus, N., et al. (2009). Selective subcortical enhancement of musical intervals in musicians. *J Neurosci*, *29*, 5832–5840.

Liberman, A. M. (1954). *The Role of Consonant-Vowel Transitions in the Perception of the Stop and Nasal Consonants*. Washington, DC: American Psychological Association.

Lins, O. G., & Picton, T. W. (1995). Auditory steady-state responses to multiple simultaneous stimuli. *Electroencephalogr Clin Neurophysiol*, *96*, 420–432.

Luo, F., Wang, Q., Kashani, A., et al. (2008). Corticofugal modulation of initial sound processing in the brain. *J Neurosci*, *28*, 11615–11621.

Maddieson, I. (1984). *Patterns of Sounds.* Cambridge [Cambridgeshire], NY: Cambridge University Press.

Moore, D. R. (1991). Anatomy and physiology of binaural hearing. *Audiology*, *30*, 125–134.

Moulines, E., & Charpentier, F. (1990). Pitch-synchronous waveform processing techniques for text-to-speech synthesis using diphones. *Speech Commun*, *9*, 453–467.

Moushegian, G., Rupert, A. L., Stillman, R. D. (1973). Laboratory note. Scalp-recorded early responses in man to frequencies in the speech range. *Electroencephalogr Clin Neurophysiol*, *35*, 665–667.

Musacchia, G., Strait, D., Kraus, N. (2008). Relationships between behavior, brainstem and cortical encoding of seen and heard speech in musicians and non-musicians. *Hear Res*, *241*, 34–42.

Musacchia, G., Sams, M., Nicol, T., et al. (2006). Seeing speech affects acoustic information processing in the human brainstem. *Exp Brain Res*, *168*, 1–10.

Musacchia, G., Sams, M., Skoe, E., et al. (2007). Musicians have enhanced subcortical auditory and audiovisual processing of speech and music. *Proc Natl Acad Sci USA*, *104*, 15894–15898.

Nicol, T., Abrams, D., Kraus, N. (2009). *Low-frequency phase coherence in near- and far-field midbrain responses reveals high frequency formant structure in consonant-vowel syllables*. Baltimore, MD: Association for Research in Otolaryngology Symposium.

Nunez, P. L., & Srinivasan, R. (2006). *Electric Fields of the Brain: The Neurophysics of EEG* (2nd ed.). Oxford, NY: Oxford University Press.

Palmer, A., & Shamma, S. (2004). Physiological Representations of Speech. In S. Greenberg, W. A. Ainsworth, A. N. Popper, R. R. Fay (Eds). *Speech Processing in the Auditory System: Springer Handbook of Auditory Research* (Vol, 18, pp, xiv, 476). New York: Springer.

Parbery-Clark, A., Skoe, E., Kraus, N (2009a). Musical experience limits the degradative effects of background noise on the neural processing of sound. *J Neurosci, 29*, 14100–14107.

Parbery-Clark, A., Skoe, E., Lam, C., et al. (2009b). Musician enhancement for speech-in-noise. *Ear Hear, 30*, 653–661.

Parthasarathy, T., & Moushegian, G. (1993). Rate, frequency, and intensity effects on early auditory evoked potentials and binaural interaction component in humans. *J Am Acad Audiol, 4,* 229–37.

Pichora-Fuller, M. K., Schneider, B. A., Daneman, M. (1995). How young and old adults listen to and remember speech in noise. *J Acoust Soc Am*, *97*, 593–608.

Picton, T. W., & Hillyard, S. A. (1974). Human auditory evoked potentials. II. Effects of attention. *Electroencephalogr Clin Neurophysiol*, *36*, 191–199.

Plyler, P. N., & Ananthanarayan, A. K. (2001). Human frequency-following responses: Representation of second formant transitions in normal-hearing and hearing-impaired listeners. *J Am Acad Audiol*, *12*, 523–533.

Porat, B. (1997). *A Course in Digital Signal Processing.* New York: John Wiley.

Quian Quiroga, R., Sakowitz, O. W., Basar, E., et al. (2001). Wavelet transform in the analysis of the frequency composition of evoked potentials. *Brain Res Brain Res Protoc*, *8*, 16–24.

Regan, D., & Regan, M. P. (1988). The transducer characteristic of hair cells in the human ear: A possible objective measure. *Brain Res*, *438*, 363–365.

Rinne, T., Balk, M. H., Koistinen, S., et al. (2008). Auditory selective attention modulates activation of human inferior colliculus. *J Neurophysiol*, *100*, 3323–3327.

Robinson, K. (1995). The duration required to identify the instrument, the octave, or the pitch chroma of a musical note. *Music Perception, 13*, 1–15.

Robinson, K., & Patterson, R. D. (1995). The stimulus duration required to identify vowels, their octave, and their pitch chroma. *J Acoust Soc Am*, *98*, 1858–1865.

Rosen, S. (1992). Temporal information in speech: Acoustic, auditory and linguistic aspects. *Philos Trans R Soc Lond B Biol Sci*, *336*, 367–373.

Russo, N., Nicol, T., Musacchia, G., et al. (2004). Brainstem responses to speech syllables. *Clin Neurophysiol*, *115*, 2021–2030.

Russo, N., Nicol, T., Trommer, B., et al. (2009). Brainstem transcription of speech is disrupted in children with autism spectrum disorders. *Dev Sci*, *12*, 557–567.

Russo, N., Nicol, T., Zecker, S., et al. (2005). Auditory training improves neural timing in the human brainstem. *Behav Brain Res*, *156*, 95–103.

Russo, N., Skoe, E., Trommer, B., et al. (2008). Deficient brainstem encoding of pitch in children with autism spectrum disorders. *Clin Neurophysiol*, *119*, 1720–1731.

Shannon, R. V. (2005). Speech and music have different requirements for spectral resolution. *Int Rev Neurobiol*, 121–134.

Sininger, Y. S. (1993). Auditory brain stem response for objective measures of hearing. *Ear Hear*, *14*, 23–30.

Skoe, E., Nicol, T., Kraus, N. Phase coherence and the human brainstem response to consonant-vowel syllables. Baltimore, MD: Association for Research in Otolaryngology Symposium.

Skoe, E., & Kraus, N. (2009). *The Effects of Musical Training on Subcortical Processing of a Missing Fundamental Piano Melody.* Chicago, IL: Society for Neuroscience.

Song, J. H., Banai, K., Russo, N. M., et al. (2006). On the relationship between speech- and nonspeech-evoked auditory brainstem responses. *Audiol Neurootol*, *11*, 233–241.

Song, J. H., Skoe, E., Wong, P. C., et al. (2008). Plasticity in the adult human auditory brainstem following short-term linguistic training. *J Cogn Neurosci*, *20*, 1892–1902.

Starr, A., & Don, M. (1988). Brain Potentials Evoked by Acoustic Stimuli. In T. W. Picton (Ed.). *Handbook of Electroencephalography and Clinical Neurophysiology (Revised Volume 3). Human Event-Related Potentials* (pp, 97–157). Amsterdam: Elsevier.

Starr, A., Picton, T. W., Sininger, Y., et al. (1996). Auditory neuropathy. *Brain, 119* (Pt 3), 741–753.

Strait, D., Kraus, N., Parbery-Clark, A., et al. (2009a). Musical experience shapes top-down mechanisms: evidence from masking and auditory attention performance. [Published ahead of print December 21, 2009.] *Hear Res*, doi:10.1016/j.heares.2009.12.021.

Strait, D., Kraus, N., Skoe, E., et al. (2009b). Musical experience and neural efficiency: Effects of training on subcortical processing of vocal expressions of emotion. *Eur J Neurosci*, *29*, 661–668.

Copyright © Lippincott Williams & Wilkins. Unauthorized reproduction of this article is prohibited.

Swaminathan, J., Krishnan, A., Gandour, J. T., et al. (2008). Applications of static and dynamic iterated rippled noise to evaluate pitch encoding in the human auditory brainstem. *IEEE Trans Biomed Eng*, *55*, 281–287.

Tallal, P., & Stark, R. E. (1981). Speech acoustic-cue discrimination abilities of normally developing and language-impaired children. *J Acoust Soc Am*, *69*, 568–574.

Thornton, A. R. D. (2007). Instrumentation and Recording Parameters. In R. F. Burkard, J. J. Eggermont, M. Don (Eds). *Auditory Evoked Potentials: Basic Principles and Clinical Application* (pp, 42–72). Philadelphia, PA: Lippincott Williams & Wilkins.

Turner, C. W., Fabry, D. A., Barrett, S., et al. (1992). Detection and recognition of stop consonants by normal hearing and hearing impaired listeners. *J Speech Hear Res*, *35*, 942–949.

Tzounopoulos, T., & Kraus, N. (2009). Learning to encode timing: Mechanisms of plasticity in the auditory brainstem. *Neuron*, *62*, 463–469.

van Drongelen, W. (2007). *Signal Processing for Neuroscientists: Introduction to the Analysis of Physiological Signals*. Burlington, MA: Academic Press.

Wallisch, P. (2009). *Matlab for Neuroscientists: An Introduction to Scientific Computing in Matlab*. Amsterdam, Boston: Elsevier/Academic Press.

Wang, J., Nicol, T., Skoe, E., Sams, M., Kraus, N. (2010). Emotion and brainstem responses to speech. *Neuroscience Letters.* doi:10.1016/j.neulet.2009.12.018.

Weinberger, N. M. (2007). Associative representational plasticity in the auditory cortex: A synthesis of two disciplines. *Learn Mem*, *14*, 1–16.

Wible, B., Nicol, T., Kraus, N. (2004). Atypical brainstem representation of onset and formant structure of speech sounds in children with language-based learning problems. *Biol Psychol*, *67*, 299–317.

Wible, B., Nicol, T., Kraus, N. (2005). Correlation between brainstem and cortical auditory processes in normal and language-impaired children. *Brain*, *128*, 417–423.

Wile, D., & Balaban E. (2007). An auditory neural correlate suggests a mechanism underlying holistic pitch perception. *PLoS One, 2*, e369.

Wong, P. C., Skoe, E., Russo, N. M., et al. (2007). Musical experience shapes human brainstem encoding of linguistic pitch patterns. *Nat Neurosci*, *10*, 420–422.

Xu, Y., Krishnan, A., Gandour, J. T. (2006). Specificity of experience-dependent pitch representation in the brainstem. *Neuroreport*, *17*, 1601–1605.

Young, E. D., & Sachs, M. B. (1979). Representation of steady-state vowels in the temporal aspects of the discharge patterns of populations of auditory-nerve fibers. *J Acoust Soc Am*, *66*, 1381–1403.

Zatorre, R. J., Belin, P., Penhune, V. B. (2002). Structure and function of auditory cortex: Music and speech. *Trends Cogn Sci*, *6*, 37–46.

Ziegler, J., Pech-Georgel, C., Florence, G., et al. (2009). Speech-perception-in-noise deficits in dyslexia. *Dev Sci, 12*, 732–745

REFERENCE NOTES

1. Chandrasekaran, B., & Kraus, N., (in press). Music and noise exclusion: Implications for individuals with learning disabilities. *Music Perception*.
2. Krizman, J., Skoe, E., Kraus, N. (in press). Stimulus rate and subcortical auditory processing of speech. *Audiol Neurotol.*

Copyright © Lippincott Williams & Wilkins. Unauthorized reproduction of this article is prohibited.
