# Supplementary material for: Neural Processing of Speech Sounds in ASD and First-Degree Relatives
Source: J Autism Dev Disord. 2022 Jun 7;53(8):3257–71. doi: 10.1007/s10803-022-05562-7 (PMC10019095; doi:10.1007/s10803-022-05562-7)
Supplement: Supplementary file 63 — Supplementary file63 (PDF 326 kb) [file 10803_2022_5562_MOESM63_ESM.pdf]

bt_gui_BioMARK_multi: the BioMARK module of the Brainstem toolbox for performing time and frequency domain analyses on BioMARK Navigator Pro AEP files

Before you begin
The first step is to export the brainstem response from the Navigator Pro AEP program and save it as a text file. Once they have been exported they can be analyzed using bt_gui_BioMARK_multi. “Multi” refers to the fact that multiple files can be interativiely proccessed.

The utility for exporting from AEP is called “AEP2ASCII” and it is available upon request by contacting Travis White-Schwoch (tws@u.northwestern.edu).

See appendix I (below) for information on how to add peak latencies to the exported response file.

How to use bt_gui_BioMARK_multi
1. Launch Matlab and browse to the folder containing the Toolbox functions.
2. At the command prompt, type **bt_gui_BioMARK_multi** and hit enter.
   You will see the “Brainstem Toolbox” [BioMARK] interface.
   See the directions below for assistance in filling in the appropriate fields.

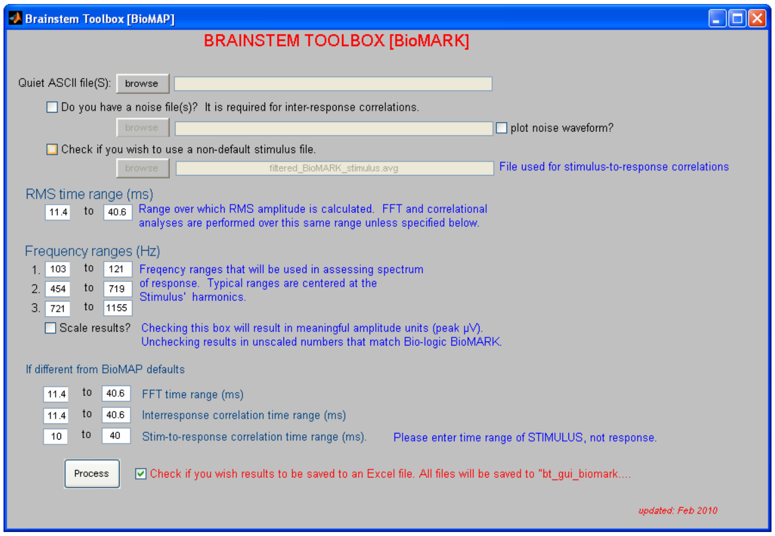

3. click on the “browse” button to select data files to be analyzed. Windows Explorer browser will open allowing you to select the appropriate files. **Only files with a .txt extension may be used**. You can select more than one file by holding down the CNTRL button.

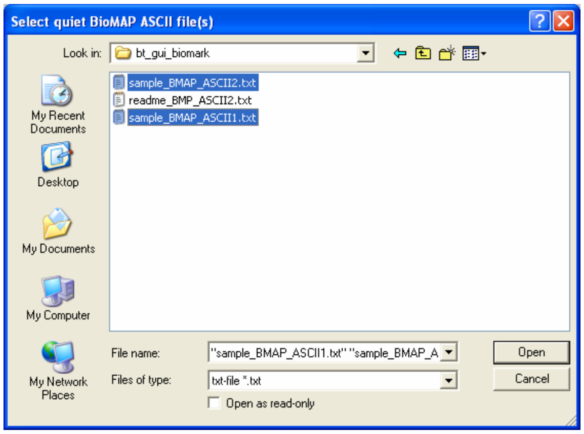

4. If you wish to correlate a noise file, check the box to the left of "Do you have a noise file?". The "browse" button will become active, allowing you to use Windows Explorer to select the noise files. This also must be in text format. If you are analyzing more than one file, you will need to select the noise files in the same order as you selected the files in step 3.

5. Check the box to the left of "plot noise waveform?" if you wish to have both the quiet and noise waveforms plotted on the resultant time domain and frequency domain graphs.

6. The default stimulus is a filtered /da/. If your quiet and noise files were collected with a non-default stimulus, you must select "Check if you wish to use a non-default stimulus file" and browse to the appropriate .avg stimulus file. (Note: if your stimulus file is in wave format, you must first convert it into an avg file. To do this, you can use wav2avg. Please see the appendices regarding this mfile.)

9. If you do not wish to use the default values, you may enter your own directly into the appropriate text boxes in the GUI.

10. If you wish to have the resultant analysis information saved to an Excel file (bt_biomark.xls), The Excel file that is created will be saved in a subfolder within the Brainstem Toolbox. This subfolder is called “outputFiles”. Each time you process a file, it will be appended into the next available row in the Excel file.

The Excel file contains all of the values calculated by the GUI. Values shown in Matlab as NaN (not a number) appear in Excel as 65535 and -999 and should be ignored.

RESULTS SCREEN

Depending on whether or not "plot noise waveform" was selected, the Brainstem Toolbox results screen will look similar to one of the following:

Without noise waveform displayed:

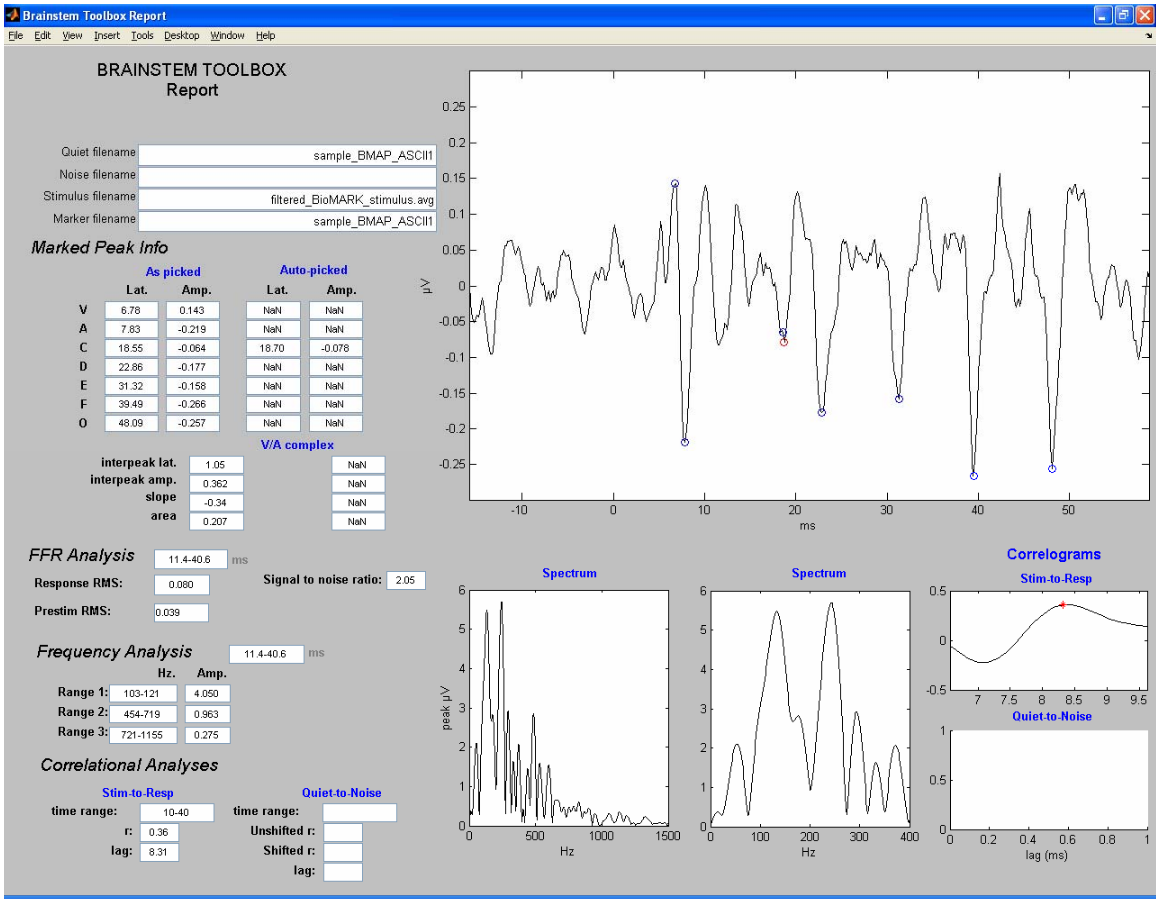

With noise waveform displayed:

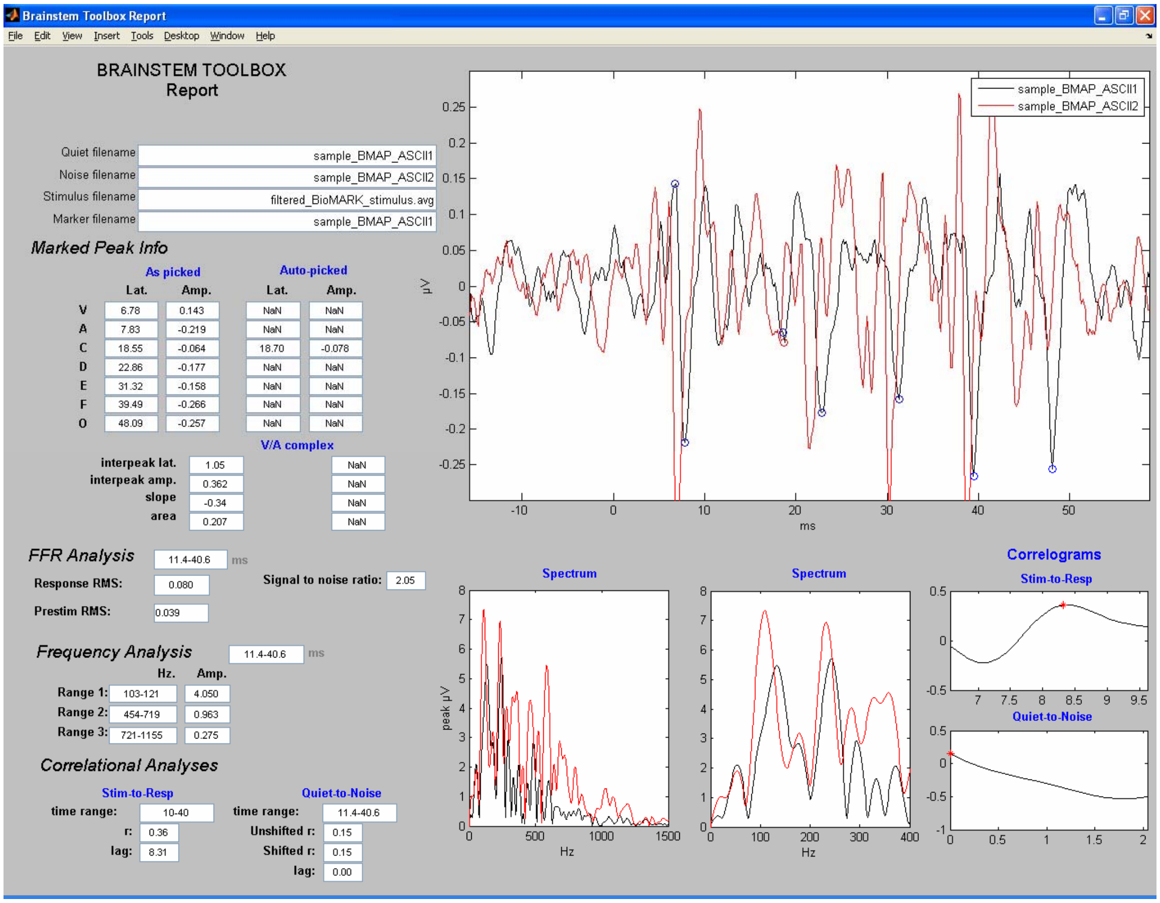

1. MARKED PEAK INFO

"Picked" peaks are indicated on the quiet waveform by blue circles at the location picked, and their latency values and amplitudes are displayed to the left of the waveform under Marked Peak Info "As Picked." If Brainstem Toolbox agreed with the picked peak, the "Auto-picked" latency and amplitude values will display simply as "NaN." If Brainstem Toolbox auto picked a different peak, the latency and amplitude values will be displayed and these points will be illustrated by red circles in the figure. This occurs when the picked peak is not precisely on the maximum or minimum amplitude of the peak and is more than +/- 2 points away from the maximum or minimum.

2. V/A COMPLEX

This section gives the interpeak latency, interpeak amplitude, slope and area specific to the peaks V and A picked automatically or specified by the user.

3. FFR ANALYSIS

This information is specific to the peaks following the V/A complex, and gives the root mean squared (RMS) amplitude of both the response and the prestimulus period, and

calculates the signal to noise ratio. These values are calculated over the specified time interval (11.4-40.6 by default), which can be changed on the main GUI interface screen.

4. FREQUENCY ANALYSIS
This function calculates the mean amplitudes over ranges of frequencies, specifically the F0, F1, and HF ranges. These range values are given by default as 103-121, 454-719, and 721-1155, respectively, and may be changed on the main GUI interface screen. The amplitude values listed are given in BioMARK "units" unless the "scale results" option was checked. If the scale results option was checked, the values listed are in microvolts (uV).

5. CORRELATIONAL ANALYSES
This section gives both stimulus-to-response and quiet-to-noise relationships (if noise file selected) as determined by the Brainstem Toolbox. The time ranges used here are the default values unless specified differently in the main GUI interface screen.

6. CORRELOGRAMS
These are graphical representations of the information summarized by the Frequency and Correlational Analyses.

7. PRINTING
Select File → Print from the toolbar to print the report.

Appendices

Appendix I. Marking Peaks

The Bio-logic BioMARK software will only allow you to mark peaks V and A. However, for our lab-internal analysis, we pick 7 peaks (V, A, C, D, E, F,O). If you want to analyze additional peaks, you will need to do the following:

(1) Before exporting the brainstem waveform using AEP to ASCII, pull up the calculated waveform in Navigator Pro and use the cursor to find the latency of the peaks you want to analyze. **Write down the latencies.**

(2) Export data using AEP to ASCII

(3) Open up ascii file using a text editor such as Notepad and enter in the latencies you wrote down.

a. Find the line starting with "Chan1 Bank1". (The numbers inside " " are the latencies of the peaks).

```
"Chan 1 Bank 1"    "6.81"   "7.69"   ""   ""   ""   ""   ""   ""   ""   ""
```

b. Enter in the latencies you wrote down (example below)

```
"Chan 1 Bank 1"    "6.96"   "7.98"   "18.77"   "23.0"   "31.32"   "41.09"   "48.39"   ""   ""   ""
```

For an example, see "sample_BMAP_ASCII1.txt" in the sample_files folder

Appendix II. Convert .wav to .avg

wav2avg utility. Resamples a standard ".wav" file specified sampling rate and converts to ".avg" format. This included command-line utility will prepare a sound file for analysis by the Pitch Tracking module. From the Matlab command line, type wav2avg('filename.wav',fs), where fs is the desired sampling rate. "filename.avg' will be created. If the .wav file is stereo, only the left channel will be converted. If this does not suit your needs, use your favorite sound edit or to reformat the .wav file to be monaural or to have the signal of interest in the left channel.

wav2avg.m can be found in the programFiles folder.
