# Supplementary material for: Neural Processing of Speech Sounds in ASD and First-Degree Relatives
Source: J Autism Dev Disord. 2022 Jun 7;53(8):3257–71. doi: 10.1007/s10803-022-05562-7 (PMC10019095; doi:10.1007/s10803-022-05562-7)
Supplement: Supplementary file 64 — Supplementary file64 (PDF 366 kb) [file 10803_2022_5562_MOESM64_ESM.pdf]

bt_gui: the “main module” of the Brainstem toolbox for performing time and frequency domain analyses

How to use bt_gui:

1. Launch Matlab and browse to the folder containing the Toolbox functions.
2. At the command prompt, type bt_gui and hit enter.

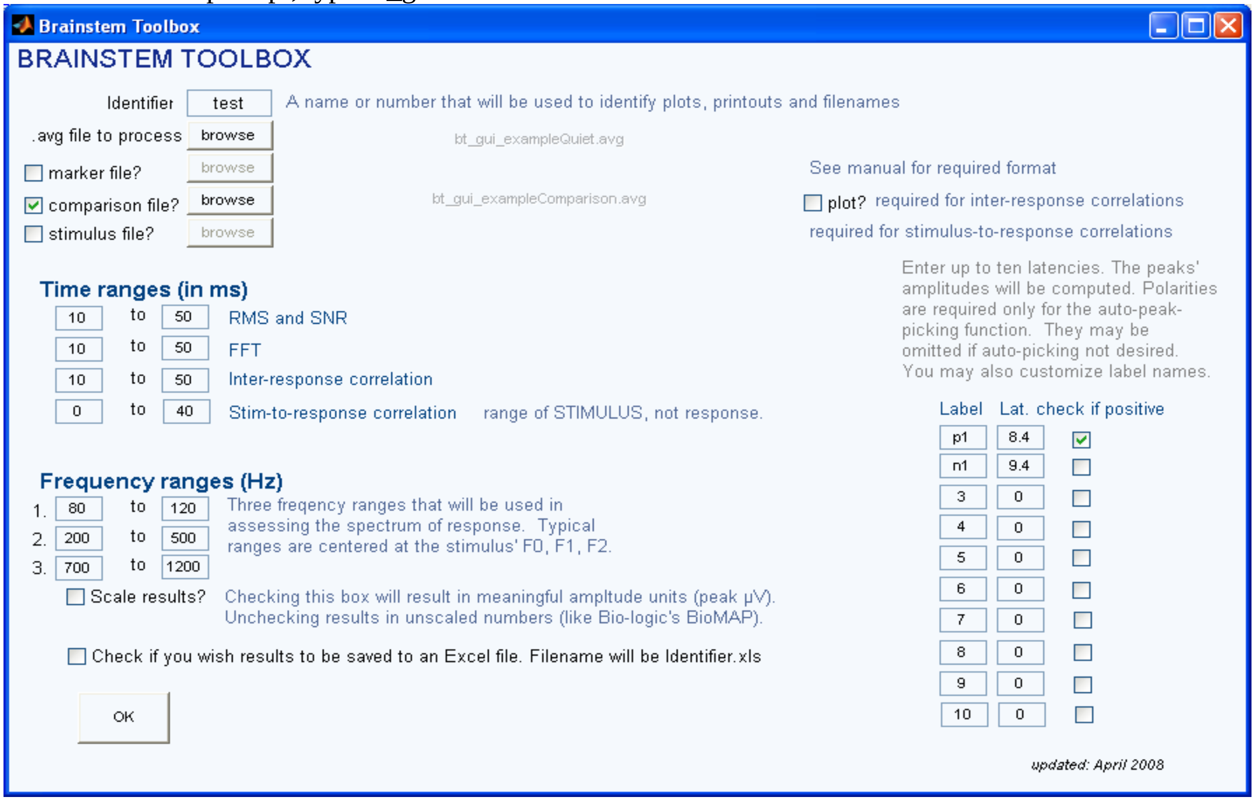

3. You will see the “Brainstem Toolbox” interface. See the directions below for assistance in filling in the appropriate fields. Specific examples illustrated in the picture are highlighted in red.
   - Type in an identifier. This can be anything, but should contain only characters that are valid for filenames because it will become the name of the Excel file that can optionally by generated. **In the example test is the identifier.**
   - Browse for the response file by clicking on the uppermost browse button under the Identifier. File must be in Neuroscan’s .avg format. For information on converting text files to this format, refer to the appendix. **In the example the response file is bt_gui_exampleQuiet.avg**
     If you have multi-channel recordings, you will be prompted to select the channel to process. See appendix section for more information on multi-channel recordings and details regarding inter-response correlations.
   - **(optional)** If you have a text file containing marked latency information, check the appropriate box and browse for this file. This is not required; however, if selected, the marked latencies and corresponding amplitudes will appear on the output screen. They will also be organized into the Excel file if it is generated. You may also hand-enter latencies directly into the Brainstem Toolbox interface (see below). For information on the required format for this text file, please refer to the appropriate section of the appendix.

- **(optional)** If you have a second file (e.g., a response to the same stimulus collected in background noise), check the 'comparison file?' box and browse for this .avg file. This comparison file will be correlated with the "primary" .avg file as part of the analysis. No other analyses will be performed on this file. Even if you have a second file, you may prefer to only display the primary file on the output screen. The "plot?" checkbox allows you to select whether or not you would like the second file to be plotted with the first in the report. **In the example, bt_gui_exampleComparison.avg, has been selected but is not being plotted**
- **(optional)** If you would like to analyze your file with respect to the stimulus, check the appropriate box and browse to the stimulus file, which must also be in .avg format. Again, for information on converting .wav files to .avg format, see this section in the appendix. See appendix section for more details regarding inter-response correlations
- "Time Ranges" is a set of latencies over which analyses are run. All of these have default values that may be set by the user by modifying bt_gui.m directly. Please contact Travis White-Schwoch (tws@u.northwestern.edu) if you need more information on how to change default values.
    - *RMS and SNR.* An RMS calculation will be performed over a specified time range. This same range will be used to compute the SNR, comparing the RMS of the response to the RMS of the prestimulus period. **In this example, the time range is 50 to 150, as this is the meat of the FFR region of the response.**
    - *FFT.* A frequency-domain analysis is performed over this time range. **50 to 150 ms in this example.**
    - *Interresponse correlation.* This only applies if a 'comparison file' is specified. A Pearson's correlation is performed between the two responses over this time range. See appendix section for more details regarding inter-response correlations. **50 to 150 ms**
    - *Stim-to-Response correlation.* This only applies if a 'stimulus file' is specified. Enter the time range of the stimulus. This is a cross-correlation technique that slides the stimulus and response back and forth in time with respect to one another. The given range of the stimulus is slid until the maximum correlation with the response is found. This will occur at some milliseconds' delay due to the neural conduction time of the auditory system. Note: this analysis is applicable only if there is a periodic element of the stimulus that evokes a frequency-following response. See appendix section for more details regarding stimulus-to-response correlations **This analysis is not being done, so we are leaving the default values.**
- "Frequency Ranges." You may specify up to three ranges over which to compute an average amplitude in the frequency domain. For example, if your stimulus is a two-tone complex at 400 and 600 Hz, you may want to specify ranges encompassing 400, 600 and 200 (F2-F1) Hz. **80-120, 180-220, 280-320 Hz are used in the example. These values span the fundamental frequency and the next 2 harmonics.**
- "Scale results." This checkbox determines whether frequency-domain results are expressed in peak microvolts, or unscaled sampling units. **In the example, the results will be scaled.**
- Latencies. You may enter latencies for up to 10 peaks. Enter a label and the latency and use the checkbox to toggle whether it is a positive-going or negative-going peak. Default is negative. Corresponding amplitudes will be computed. This assumes that latencies were already chosen using other software. **One prominent positive peak and one prominent negative peak are named and latencies are entered.**

4. Tick the "save to Excel" box to generate an Excel file with the analysis results. (see appendix)
5. Click the "OK" button and after a few moments a results screen will appear. See examples 1 and 2 below.

Results Screen, Example 1 (Peak Analysis performed)

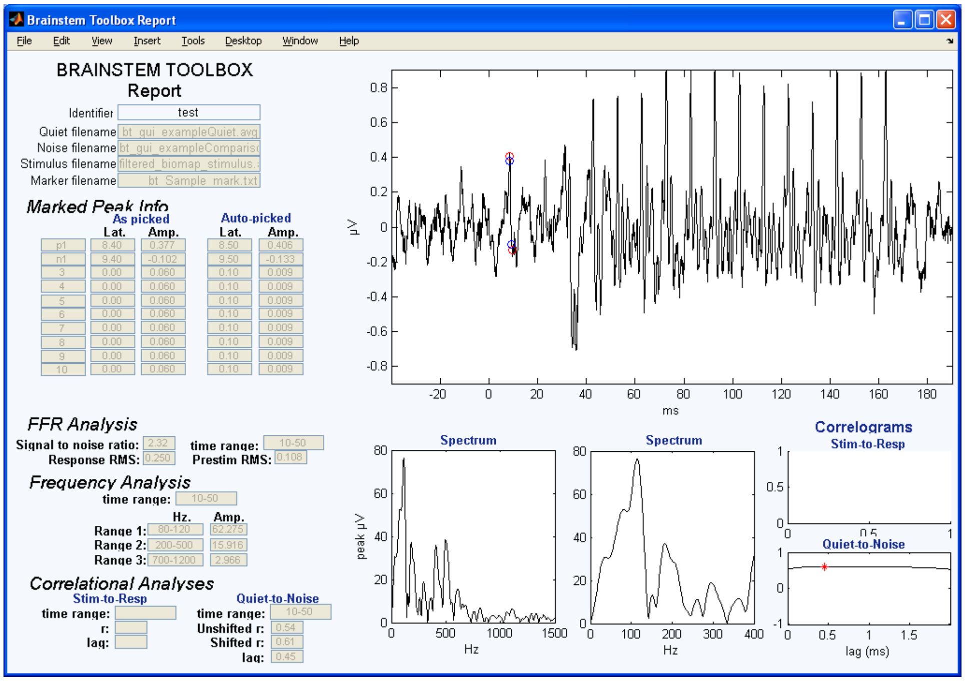

Results Screen, Example 2 (Noise file was plotted, Stim-to-Response Correlation was performed)

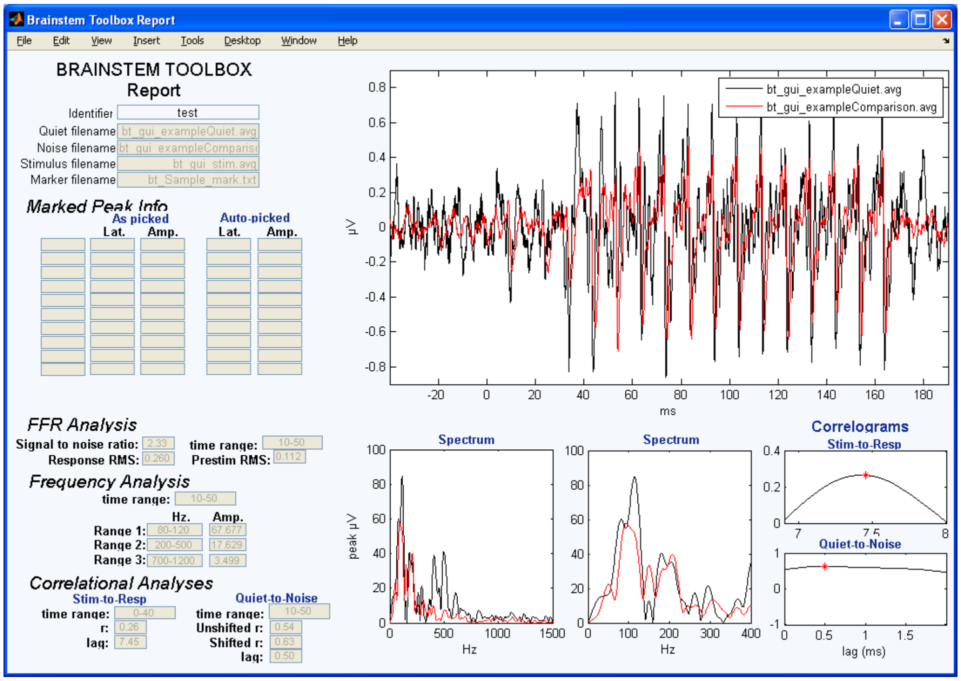

DESCRIPTION OF RESULTS SCREEN

- The identifier and relevant filenames are listed at the top.
- The response waveform is plotted on the top right with the chosen peaks marked in blue and red (see next item). It you have chosen that the comparison waveform be plotted, it appears in red.
- The latencies and corresponding amplitudes of the peaks that were entered are listed in the *as picked* column of the "Marked Peak Info" table, and plotted as blue circles on the waveform. The *auto-picked* column contains the actual maximum or minimum amplitude within two (+/-2) sample points of the *as picked* latency if different. These are plotted as red circles — the middle of the circle indicates the precise latency of the peak . **In example 1, the p1 and n1 *as picked* and *auto-picked* values are not identical. The Toolbox found that the latencies entered above (8.4 and 9.4, respectively) were not the actual local minima. It found the true minima and returned those latencies (8.5 and 9.5, respectively) and corresponding amplitudes.** The unused "marked peaks" can be disregarded they are reported as 0s (zeros).
- *FFR Analysis.* The RMS amplitudes over the specified time range and over the prestimulus period are computed. These two values are used to compute the signal-to-noise ratio.
- *Frequency Analysis.* The results of a fast Fourier analysis over the specified time region are given. The mean amplitudes (in peak µV or sampling units depending on chosen scaling option) over the specified frequency ranges are given. The spectrum, up to 1500 Hz and up to 400 Hz, is plotted. (Technical note: the sample is zero-padded to the sampling rate to result in a frequency resolution of 1 Hz.) It you have chosen that the comparison waveform be plotted, it appears in red.
- *Correlational Analyses.* If secondary (e.g. "noise") and stimulus waveforms are provided, two cross correlations are performed. Both result in "lag" values: the amount (in ms) that the waveforms were shifted to obtain the maximum correlation. The "straight" quiet-to-noise correlation is also reported (0 lag). Correlograms (lag *x* r) are plotted to the right with a star at the maximum. Note: Although the comparison file may be anything, it is assumed that the typical response-to-response correlation will be a quiet to noise comparison, so that is how the output screen is labeled. See Appendix VI for more information.
- *Print.* Select File → Print from the toolbar to print the results screen that has been generated.

Appendices

Appendix I. Convert text to .avg
This process can be skipped if the brainstem responses are collected using Neuroscan Acquire. Please see special instructions for Intelligent Hearing Systems (IHS) data, which can be found within the "converting IHS files" folder of the "Tutorials" folder distributed with the toolbox.

This is the first step before any BT functions are usable.   This step will convert the txt file to a Neuroscan-format .avg file. Note: it is not necessary to have Neuroscan software to use the BT functions. This is simply a convenient file format.

From command prompt in Matlab, type

```
>> bt_txt2avg('filename.txt', 6855, -15.8, 58.89);
```

where filename.txt is the exported brainstem data file, 6855 is the sampling rate, -15.8 corresponds to the start time (ms) of the response epoch and 58.89 corresponds to the time at the end of the response epoch. (In this example, the recording window is 74.69 ms)

bt_txt2avg.m can be found in the programFiles folder.

Appendix II. Format of marker file.
Save peak information in a text file with no header, one peak below the other (use a text editing program such as notepad) The contents of the file should look like this:

```
A       5       1
B       10      1
C       15      0
D       20      0
E       25      0
F       30      1
G       35      0
H       40      1
```

Column 1 is the Peak Label, Column 2 is the Peak Latency, Column 3 indicates whether the peak is positive (1) or negative (0).
See "sample_marker_file.txt" in the sample_files folder.

Appendix III. Convert .wav to .avg
wav2avg utility. Resamples a standard ".wav" file specified sampling rate and converts to ".avg" format. This included command-line utility will prepare a sound file for analysis by the Pitch Tracking module. From the Matlab command line, type wav2avg('filename.wav',fs), where fs is the desired sampling rate. "filename.avg' will be created. If the .wav file is stereo, only the left channel will be converted. If this does not suit your needs, use your favorite sound editor to reformat the .wav file to be monaural or to have the signal of interest in the left channel.

wav2avg.m can be found in the programFiles folder.

Appendix IV. Description of Excel file

The Excel file that is generated consists of five lines of descriptive text, followed by a row of description labels, followed by the results. Not applicable results result in -999. If you process several subjects and wish to rapidly compile their data into a single place, the range of interest is A7:BK7.

The first five columns of an Excel file corresponding to our example subject 1006:

1006				
Primary file: 1006_daBase.avg				
Comparison file: 1006_B6.avg				
Stimulus File:				
Marker File:				
FFRTimeStart	FFRTimeStop	ResponseRMS	PrestimRMS	SNR
50	150	0.361072	0.127366	2.834906

"ptgui_sample_Excel_outputfile.xls" is included in the sample_files folder if you wish to examine the file structure in more detail.

Appendix V. Multi-channel recordings.

The first time you browse for a response file within a single session, you will be prompted to select the channel to process. The program will remember your selection and continue to process that channel for each subsequent file selected. You are only prompted once per session – as soon as you close Matlab this information is "forgotten". If you want to reset the ranges without first closing Matlab, type 'clear all' into the Matlab command prompt or close the brainstem toolbox interface.

You will be prompted separately for the "primary" file and "comparison" file".

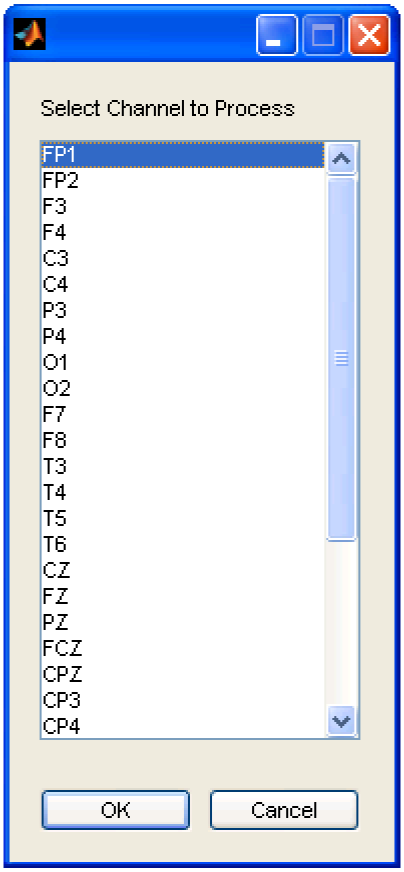

Appendix VI. Stimulus-to-Response and Inter-response correlations.

The first time you select a stimulus file within a single Matlab session, you will be prompted to enter the lag range for the correlation analysis. The program will remember your selection and continue to use that range for each subsequent comparison file/stimulus selected. You are only prompted once per session – as soon as you close Matlab this information is "forgotten". If you want to reset the ranges without first closing Matlab, type 'clear all' into the Matlab command prompt, or close the brainstem toolbox interface.

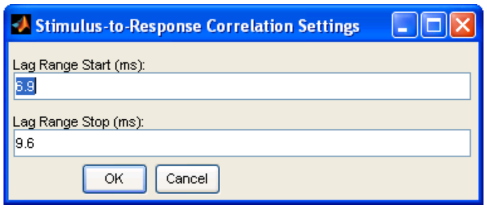

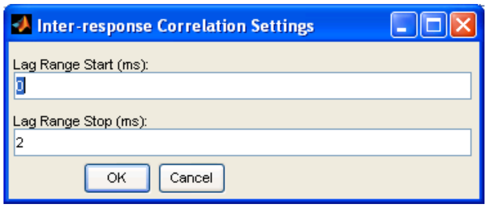

Stimulus-to-Response Correlations
The stimulus-to-response correlation procedure involves a series of Pearson's correlations between a fixed portion of the stimulus and a non-fixed portion of the response. Let's say for example that on the main interface you entered a stimulus time range of 0 to 40 ms. If you then

entered 6.9 to 9.6 for your lag ranges (see above), the first correlation would be between 10-40ms of the stimulus and 16.9-46.9 ms of the response, the last correlation would be performed between 10-40ms of the stimulus and 19.6 to 49.6 ms of the stimulus. On the results screen, you will see the r-value for each correlation (lag is plotted on x axis, r-value is plotted on y axis). A red asterisks appears at correlation maximum.

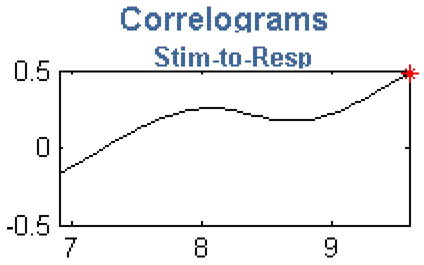

In the above example, the maximum occurs at the very edge of the correlational range. This suggests that a different lag range must be defined.

Important Note: Given the brainstem responses exhibit low-filter characteristics, we suggest that you low-pass filter your stimulus file to maximize stimulus-to-response correlational values.

Inter-Response Correlations

The Inter-responsecorrelation procedure involves a series of Pearson's correlations between a fixed portion of the primary file and a non-fixed portion of the comparison file. Let's say, for example, that on the main interface you entered 20 to 40 ms for the inter-response time range.   If you then entered 0 to 2 for your lag ranges (see below), the first correlation would be between 20-40 ms of the primary file and 20-40 ms of the secondary file (straight correlation), the last correlation would be performed between 20-40ms of the primary file and 22-42 ms of the secondary On the results screen, you will see the r-value for each correlation (lag is plotted on x axis, r-value is plotted on y axis). A red asterisks appears at correlation maximum
