# Supplementary material for: Neural Processing of Speech Sounds in ASD and First-Degree Relatives
Source: J Autism Dev Disord. 2022 Jun 7;53(8):3257–71. doi: 10.1007/s10803-022-05562-7 (PMC10019095; doi:10.1007/s10803-022-05562-7)
Supplement: Supplementary file 65 — Supplementary file65 (PDF 292 kb) [file 10803_2022_5562_MOESM65_ESM.pdf]

bt_ptgui: the "pitch tracking" (pt) module

Introduction

The pitch tracking module of the Brainstem Toolbox enables you to determine how well the frequency-following response follows the periodicity of the evoking stimulus. It analyzes a response, both in its own right, and with respect to the stimulus to provide metrics of the degree to which the response "locks" to the stimulus.

<u>Suggested Reading:</u>

Song JH, Skoe E, Wong PCM, Kraus N. (2008) Plasticity in the adult human auditory brainstem following short-term linguistic training. *J Cogn Neurosci*.

Wong PCM, Skoe E, Russo NM, Dees T, Kraus N. (2007) Musical experience shapes human brainstem encoding of linguistic pitch patterns. *Nature Neurosci* 10:420-422.

Krishnan A, Xu Y, Gandour J, Cariani P. (2005) Encoding of pitch in the human brainstem is sensitive to language experience. *Cogn Brain Res* 25(1):161-8.

Description of the pitch tracking module

Before describing how to use the pitch tracking module, we will first look at the result of the analysis. This should make the description of how to use it easier to understand.

A picture of the output screen is below, descriptions of the elements follow:

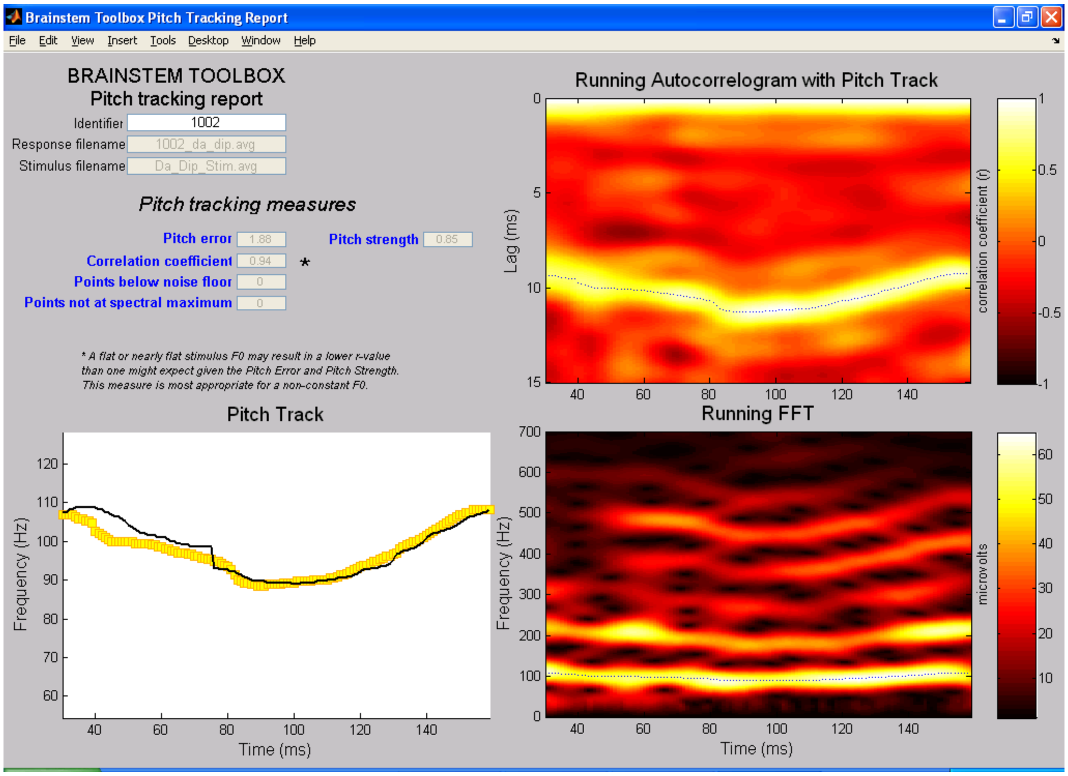

- First, look at the **Pitch Track** plot on the lower left, and the left-column values listed under Pitch Tracking measures. The black line is the "pitch," or fundamental periodicity of the stimulus, as determined by the frequency estimation method (either autocorrelation or FFT, see appendix IV for more information). In this case, the stimulus was the spoken syllable "da" with a dipping pitch (fundamental frequency) contour ranging from about 90-110 Hz. The yellow boxes are the response's periodicity. The **Pitch Error** value of "2.07" indicates that, on average, the response differed from the stimulus' pitch by 2 Hz. This number is an absolute value: it does not matter if the response is "sharp" or "flat with respect to the stimulus." "**Correlation coefficient**" is the Pearson's r between the stimulus-track and the response-track lines. Given the nature of Pearson's correlations, the measure is most appropriate for a non-constant F0. A flat or nearly flat F0 may result in a lower r-value that one might expect given the Pitch Error and Pitch Strength.
- The small dots above the Pitch track plot signify regions that are below the noise floor (red) and where the response frequency plotted was not the maximum frequency in the specified spectrum range (blue). These numbers (4 and 24, respectively) are listed above. Because you can not determine instantaneous pitch of a point in time; each point on the graph denotes the midpoint of a time chunk (in this case 40 ms). The number on the x-axis signifies the midpoint of this chunk. Thus, in this example, the first point, at time 20, signifies a chunk from 0 to 40 ms. To account for neural conduction time, the stimulus is "shifted back" in time by an amount provided by the user so that the two contours are maximally aligned in the figure.
- Next, look at the **Running Autocorrelogram with Pitch Track** plot at upper right. This analysis also uses a short-time running autocorrelation technique, but provides a fuller picture of the response's phase locking. The response, again chopped into 40-ms chunks, is successively time-shifted with a delayed ("lagged") version of itself (in 1 ms steps) and a Pearson's r is calculated at each 1-ms interval. The highest possible periodicity would result in an r of +1. An r of -1 indicates fully out-of-phase. Thus, high, positive r's indicate periodicity in the signal. Time (midpoint of 40-ms time chunk) is on the x-axis, "lag" is on the y-axis, and r-value is depicted by color. Lag is 1/frequency; or, relevant to the plot which is expressed in milliseconds, 1000/frequency. They y-axis is inverted (0 at the top) so that the curve of the plot matches that of the Pitch Track plot. The brightest colors are the highest correlations, and appear at lags corresponding to the fundamental periodicity of the response (the same frequencies as the Pitch Track plot). **Pitch Strength** is the mean of the r's at the thin black line: those of the response's pitch. Technical note: R-values converted to Fischer z-transformed before averaging. The average z' prime value is then converted back to an r-value using an inverse Fisher transform.
- The **Running FFT** plot, is the result of a short-time Fourier transform (STFT) This is a spectrogram of the response with a thin black line signifying the response's pitch overlaying it. Brighter colors are higher energy (in arbitrary units). The frequency axis goes up to 700 Hz in order to visualize the tracking to the harmonics of the fundamental periodicity.

How to use the Pitch Tracking module of the Brainstem Toolbox.

- Launch Matlab and browse to the folder containing the Toolbox functions.
- At the command line, type "bt_ptgui." The following window will appear:

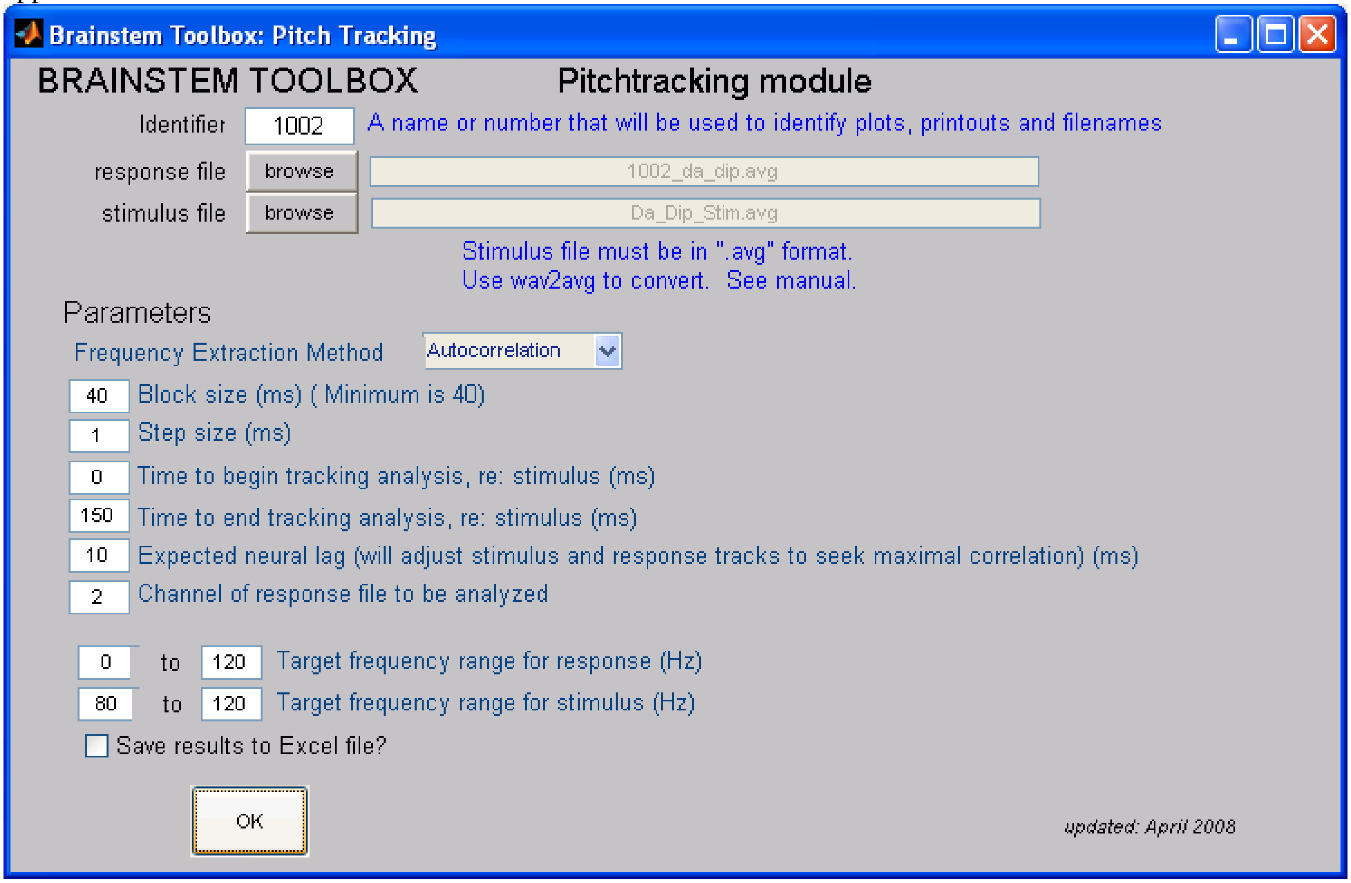

- Type in an identifier. This can be anything, but should contain only characters that are valid for filenames because it will become the name of the Excel file that (optionally) is generated.
- Browse for the response and stimulus files to be analyzed. Both must be in ".avg" format *and must have the same sampling rate*. See main Brainstem Toolbox documentation for information on how to convert text files into .avg format. See Appendix below for use of the included wav2avg utility that is used to resample and convert sound files (.wav) to .avg format.

- **Parameters**

    - **Frequency estimation method:** There are two options (1) autocorrelation (2) STFT for estimating the Pitch Track (or Harmonics Track) of the response (see appendix item IV for more information). Depending on the method you select, you will get slightly different values for the Pitch Error, Correlation Coefficient, and Pitch Track plot.
    - **Block size.** This is the size of the "chunk" used in the sliding-window STFT and autocorrelation analyses. 40 ms is the minimum, and is recommended.
    - **Step size.** This is the time resolution of the analysis. There will be a frequency estimate every *x* ms.
    - **Time to begin tracking analysis.** This is the time *in the stimulus* that the analysis will begin.

- **Time to end tracking analysis.** This is the time *in the stimulus* that the analysis will end
- **Expected neural lag.** Essentially, this is the latency of the first FFR peak. This primarily affects the pitch-error calculation. The response lags behind the stimulus by some milliseconds. The pitch error will be inflated unless the neural lag is taken into account, particularly if the pitch of the stimulus is changing rapidly. For example, if the *expected neural lag* is 10 ms, and the *time to begin tracking analysis* is 5 milliseconds, then the first 40-ms response time bin analyzed will encompass 15-55 ms. In the Pitch Track plot that gets generated, the stimulus Pitch Track is shifted back in time by the value corresponding to the *expected neural lag*. In other words, the 15-55 ms response segment will be plotted against the 5-45 ms stimulus segment.
- **Channel of response.** If the .avg file of the response has more than one channel. IMPORTANT NOTE: There is no way to specify which channel of the stimulus is to be analyzed. If the original *stimulus* .wav file has more than one channel (i.e. a stereo signal), only channel 1 (left) is used in its conversion to .avg. Make sure that the stimulus file is formatted appropriately.
- **Target frequency ranges (stimulus/response).** This guides the algorithm to look in restricted windows based on the known/expected frequency.
- **Save to Excel file**. If checked, the five values on the output screen are saved to a file. The Excel is saved as “Identifier_pt.xls” in the “outputFiles” folder.
- **Click “OK”** button and after a few moments a results screen will appear. (Be patient!)

Appendices

Appendix I. Convert text to .avg

This process can be skipped if the brainstem responses are collected using Neuroscan Acquire. Please see special instructions for Intelligent Hearing Systems (IHS) data, which can be found within the "converting IHS files" folder of the "Tutorials" folder distributed with the toolbox.

This is the first step before any BT functions are usable. This step will convert the txt file to a Neuroscan-format .avg file. Note: it is not necessary to have Neuroscan software to use the BT functions. This is simply a convenient file format.
From command prompt in Matlab, type

```
>> bt_txt2avg('filename.txt', 6855, -15.8, 58.89);
```

where filename.txt is the exported brainstem data file, 6855 is the sampling rate, -15.8 corresponds to the start time (ms) of the response epoch and 58.89 corresponds to the time at the end the response epoch. (In this example, the recording window is 74.69 ms)

Appendix II. Convert .wav to .avg

wav2avg utility. Resamples a standard ".wav" file specified sampling rate and converts to ".avg" format. This included command-line utility will prepare a sound file for analysis by the Pitch Tracking module. From the Matlab command line, type wav2avg('filename.wav',fs), where fs is the desired sampling rate. "filename.avg' will be created. If the .wav file is stereo, only the left channel will be converted. If this does not suit your needs, use your favorite sound editor to reformat the .wav file to be monaural or to have the signal of interest in the left channel.

Appendix III. Description of Excel file

The Excel file that is generated consists of four lines of descriptive text, followed by a row of description labels, followed by the results. If you process several subjects and wish to rapidly compile their data into a single place, the range of interest is A6:E7.

The first five columns of an Excel file corresponding to our example subject 1002:

	1002			
Response: 1002_da_dip.avg				
Stimulus: Da_Dip_Stim.avg				
Number of time chunks: 130				
Pitch Error	Pitch Strength	Correlation	# below Noise Floor	# Not Spectral Max
1.884517423	0.811585104	0.939020406	0	0

Appendix IV. Frequency Estimation Method

We are giving you the option to estimate frequency-tracking using two different methods: (1) autocorrelation (2) STFT. Autocorrelation, estimates the pitch, by finding the fundamental period and taking its inverse (fo = 1/period). The fundamental period is calculated as the lag time that achieves the highest self-correlation. STFT, estimates the response frequency, by finding the largest spectral peak within a defined frequency range. STFT is the preferable method for tracking the harmonics of the fundamental frequency. Due to inherent methodological differences, you will get slightly different values for the Pitch Error, Correlation Coefficient, and the Pitch Track plot depending on which method you select.
