# Supplementary material for: Neural Processing of Speech Sounds in ASD and First-Degree Relatives
Source: J Autism Dev Disord. 2022 Jun 7;53(8):3257–71. doi: 10.1007/s10803-022-05562-7 (PMC10019095; doi:10.1007/s10803-022-05562-7)
Supplement: Supplementary file 66 — Supplementary file66 (DOCX 15 kb) [file 10803_2022_5562_MOESM66_ESM.docx]

**CONVERTING IHS ASCII FILES TO *.AVG FILE FORMAT:**

To convert IHS text files to *.avg files that can be used by the Brainstem Toolbox, you will need two files:

“ihsascii2avg.m”

IHS-template.avg

Both files reside in the programFiles folder of the Brainstem Toolbox (version 2013).

At the MATLAB command prompt type:

>> ihsascii2avg('c:\full\pathname\FilenameOftxtFile.txt','c:\full\pathname\IHS-template.avg');

The resultant *.avg file will be named: 'c:\full\pathname\FilenameOftxtFile.avg' and will get saved in the active directory.

Notes:

1. The second argument can be omitted if IHS-template.avg resides in active folder: ihsascii2avg('c:\full\pathname\FilenameOftxtFile.txt');
2. arguments like sampling rate and time window are NOT needed because this information is stored in the txt file's header.
3. The *.avg files that result from IHS-ascii files always will have four channels, A, B, Added, Subtracted*.

How the four channels are interpreted depends on the stimulus polarity.
--If you stimulate with a single polarity, condensation or rarefaction, A and B represent the two IHS buffers, which, in effect are every-other sweep. Added is the sum of the two buffers and Subtracted is the difference. In practice, in this case, the only logical one to analyze is "Added" unless you want to look at A and B buffers for questions of internal response-consistency.

--If you stimulate with alternating polarity, A and B are responses to the two separate polarities. Added is the sum of the two polarities and Subtracted is the difference. In this case, any or all of the four channels might be of interest depending on your needs.

1. note, the fourth channel is actually "Subtracted" due to a ten character limit for channel names in avg files.
