# Supplementary material for: Neural Processing of Speech Sounds in ASD and First-Degree Relatives
Source: J Autism Dev Disord. 2022 Jun 7;53(8):3257–71. doi: 10.1007/s10803-022-05562-7 (PMC10019095; doi:10.1007/s10803-022-05562-7)
Supplement: Supplementary file 67 — Supplementary file67 (PDF 96 kb) [file 10803_2022_5562_MOESM67_ESM.pdf]

The Brainstem Toolbox 2013

The Brainstem Toolbox is free software developed and distributed by the Auditory Neuroscience Laboratory at Northwestern University (http://www.brainvolts.northwestern.edu)

The brainstem toolbox is distributed in the hope that it will be useful, but WITHOUT ANY WARRANTY; without even the implied warranty of MERCHANTABILITY or FITNESS FOR A PARTICULAR PURPOSE.

You can redistribute it and/or modify it under the terms of the GNU General Public License as published by the Free Software Foundation. See the GNU General Public License for more details <http://www.gnu.org/licenses/>.

If you publish or present results derived in part from using this toolbox, we ask that you cite it as follows:

> Skoe E, Nicol T & Kraus N (2013). The Brainstem Toolbox. Version 2013 (www.brainvolts.northwestern.edu)

If the journal does not allow you to cite a web site, then try:

> Skoe E, Kraus N. (2010) Auditory brainstem response to complex sounds: a tutorial. *Ear and Hearing* 31(3): 302-324.

Introduction

The Brainstem Toolbox (bt) includes three graphical user-interface (GUI) modules for performing a number of analyses on evoked responses.

(1) bt_gui — the "main module" for performing time & frequency domain analyses

(2) bt_gui_biomark_multi (*New in version 2010)*
designed specifically for users of the BioMARK software
It is a paired down version of bt_gui. Multiple files can be processed iteratively.

(3) bt_ptgui — the "pitch tracking" (pt) module for assessing how well the frequency-following response follows the periodicity of the evoking stimulus.

This toolbox presents the results of these analyses in a single report for each file. This was designed with speech-evoked brainstem response in mind, so it emphasizes analyses appropriate to periodic signals, but is generalizable to responses evoked by any stimulus.

Requirements

Matlab is required. The Toolbox was developed in version 7.3 (2006b) on a PC. It has been tested on other 7.X versions. Other versions and platforms have not been verified.

What is it?

The Brainstem Toolbox is a collection of MATLAB functions (m-files). Most can be invoked from the command line, but to take fullest advantage of the Toolbox, the GUI should be used. (See help for command-line usage of individual functions.) The m-files that compose the "guts" of the program, reside in the programFiles folder.

Acknowledgments

The Toolbox consists of many of our own functions (all those prefixed with "bt_" and a few others), but also contains several functions and utilities borrowed from other sources. Please see individual m-files for author and copyright information.

New to MATLAB

The Mathworks site contains a comprehensive list of MATLAB tutorials.

http://www.mathworks.com/academia/student_center/tutorials/launchpad.html

Errors and bugs

We would like to be informed about any bugs you might encounter. Please email Travis White-Schwoch, tws@u.northwestern.edu.

Brainstem Toolbox 2013

Suggested Reading
(available for download: http://www.soc.northwestern.edu/brainvolts/publications.php)

Skoe E, Kraus N. (2010) Auditory brainstem response to complex sounds: a tutorial. *Ear and Hearing* 31(3): 302-324.

Pitch Tracking
Song JH, Skoe E, Wong PCM, Kraus N. (2008) Plasticity in the adult human auditory brainstem following short-term linguistic training. *J Cogn Neurosci.*

Wong PCM, Skoe E, Russo NM, Dees T, Kraus N. (2007) Musical experience shapes human brainstem encoding of linguistic pitch patterns. *Nature Neurosci* 10:420-422.

How to use it

1. Installation. "Unzip" the distribution file into a new folder.
2. Launch Matlab an/d browse to the folder containing the Toolbox functions. The folder contains the following subfolders and files.

Name	Size	Type	Date Modified
documentation		File Folder	5/2/2008 4:14 PM
outputFiles		File Folder	5/2/2008 4:14 PM
programFiles		File Folder	5/2/2008 4:14 PM
sample_files		File Folder	4/28/2008 7:37 AM
bt_gui.m	33 KB	MATLAB M-file	4/25/2008 3:31 PM
bt_gui_biomap.m	20 KB	MATLAB M-file	4/4/2008 9:12 AM
bt_ptgui.m	15 KB	MATLAB M-file	4/7/2008 12:29 PM

Description of subfolders:

Documentation	Tutorials and suggested readings
outputFiles	All files generated by bt get saved here.
programFiles	The "inner guts" of the bt program (m-files and fig files).
sample_files	Sample avg, txt, mrk, xls files

3. At the command prompt, type the name of the module you want to run
   Example: >> bt_gui
4. For specifics on how to use each module, refer to the respective user tutorials in the documentation folder.
