# Supplementary figures and images for: Neural Processing of Speech Sounds in ASD and First-Degree Relatives
Source: J Autism Dev Disord. 2022 Jun 7;53(8):3257–71. doi: 10.1007/s10803-022-05562-7 (PMC10019095; doi:10.1007/s10803-022-05562-7)

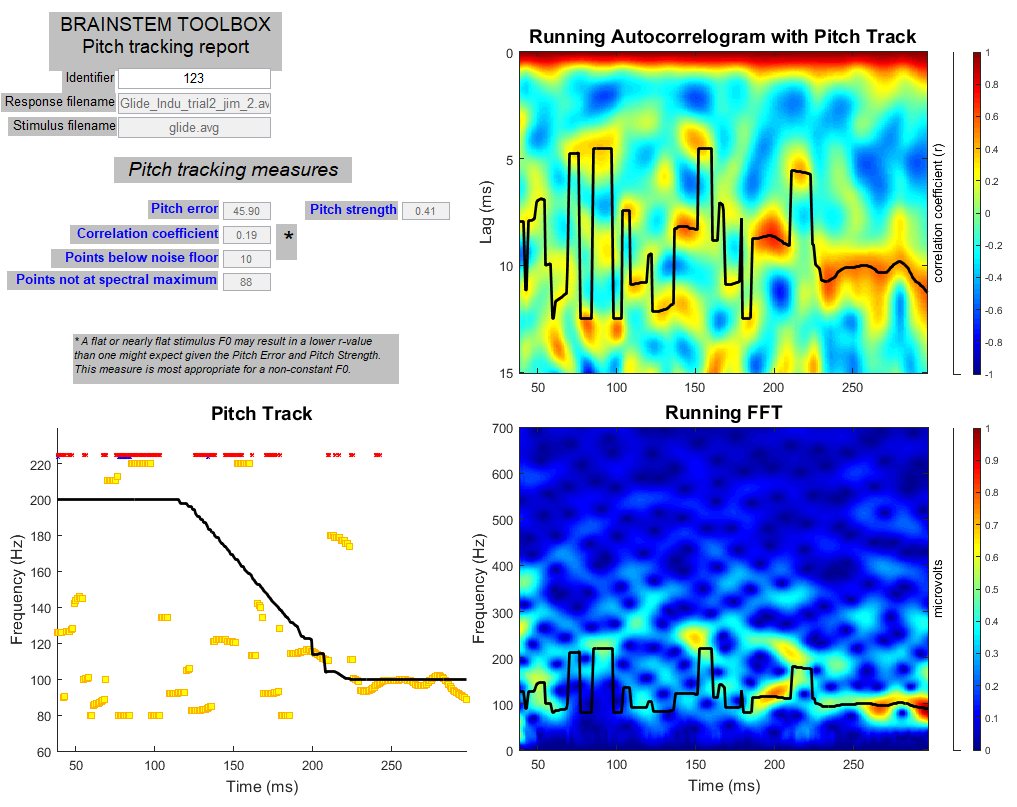

Supplement: Supplementary file 70 — Supplementary file70 (PNG 154 kb) [file 10803_2022_5562_MOESM70_ESM.png]
