# Supplementary material for: Neural Processing of Speech Sounds in ASD and First-Degree Relatives
Source: J Autism Dev Disord. 2022 Jun 7;53(8):3257–71. doi: 10.1007/s10803-022-05562-7 (PMC10019095; doi:10.1007/s10803-022-05562-7)
Supplement: Supplementary file 76 — Supplementary file76 (DOCX 15 kb) [file 10803_2022_5562_MOESM76_ESM.docx]

The Matlab functions and scripts that are used to produce the various measurements reported in the paper are homebrew and were developed and run on Matlab v. 2015a. Some functions use accessory functions that are downloadable from the Matlab file exchange and/or are included in the Brainstem Toolbox. These are described in the documentation within the code. We have noted markmatfile.m does not work as intended in some later versions due to a change in how Matlab handles datatips in figures.

A Windows utility from Natus (https://natus.com) “AEP2ASCII.exe” converts Navigator Prof files to text files. Once in text format, they are submitted to the following processing steps in Matlab.

**Processing the /d/ and click files.**

1. Organize the click and da waveforms and metadata into a Matfile with **bioascii2mat.m**. Example:
   > bioascii2mat('myDaAsciiFile.txt', 'myClkAsciiFile.txt');
   This creates a matfile with subject number and date of test as filename.
   Example: 1234_2021-01-01.mat
2. Mark peaks using a function **markmatfile.m** Example:
   > markmatfile('1234_2021-01-01.mat')
   This generates a gui with the ability to place markers on desired peaks in both the click and the da mean average. Latencies and amplitudes are saved into the mat-file.
3. Use script **extractdata.m** to compute the various measurements reported in the paper. Running this script produces a file browse dialog so that multiple mat-files can be processed together. The resulting measurements are outputted to an Excel file for further analysis elsewhere. The Excel file’s naming convention is the word “Biomark” followed by today’s date (i.e., the date the files are processed.

**Processing the /ja/ files.**

/ja/ files are processed with the “Brainstem Toolbox.” This Toolbox expects data to be in Compumedic Neuroscan’s “.avg” format.

1. Convert text files to .avg format using **convertavg.m**. (Requires writeavg.m) Example:
   > convertavg('myJaAsciiFile.txt');
   Resulting file will have same filename prefix with “.avg” suffix
2. After extracting the “Brainstem Toolbox” brose to that folder and run a gui called **bt_ptgui.m.** This requires many parameter fields to be filled in. Here are the parameters we used for this paper:

Identifier = [a unique subject ID]

Response file = Browse to .avg file created in step A

Stimulus file = Browse to ja_up3202.avg

Freq Extraction Method = chose “FFT”

Block size: 40

Step size: 1

Time to begin...: 0

Time to end...: 230

Expected neural lag: 10

Channel of response: 3

Target frequency range for response: 120 | 230

Target frequency range for response: 120 | 230

Save to excel: as desired
